# Different hydrogen isotope fractionations during lipid formation in higher plants: Implications for paleohydrology reconstruction at a global scale

**DOI:** 10.1038/srep19711

**Published:** 2016-01-25

**Authors:** Jinzhao Liu, Weiguo Liu, Zhisheng An, Hong Yang

**Affiliations:** 1 School of Human Settlements and Civil Engineering, Xi’an Jiaotong University, Xi’an 710049, China; 2 State Key Laboratory of Loess and Quaternary Geology, IEE, CAS, Xi’an 710075, China; 3 Laboratory for Terrestrial Environments, College of Arts and Sciences, Bryant University, Smithfield, RI 02917, USA

## Abstract

Leaf wax δD_*n*-alkane_ values have shown to differ significantly among plant life forms (e.g., among grasses, shrubs, and trees) in higher plants. However, the underlying causes for the differences in leaf wax δD_*n*-alkane_ values among different plant life forms remain poorly understood. In this study, we observed that leaf wax δD_*n*-alkane_ values between major high plant lineages (eudicots versus monocots) differed significantly under the same environmental conditions. Such a difference primarily inherited from different hydrogen biosynthetic fractionations (ε_wax-lw_). Based upon a reanalysis of the available leaf wax δD_*n*-alkane_ dataset from modern plants in the Northern Hemisphere, we discovered that the apparent hydrogen fractionation factor (ε_wax-p_) between leaf wax δD_*n*-alkane_ values of major angiosperm lineages and precipitation δD values exhibited distinguishable distribution patterns at a global scale, with an average of −140‰ for monocotyledonous species, −107‰ for dicotyledonous species. Additionally, variations of leaf wax δD_*n*-alkane_ values and the ε_wax-p_ values in gymnosperms are similar to those of dicotyledonous species. Therefore, the data let us believe that biological factors inherited from plant taxonomies have a significant effect on controlling leaf wax δD_*n*-alkane_ values in higher plants.

Analytical advances in compound-specific stable hydrogen isotopic analysis have facilitated the use of *n*-alkyl lipids as the biomolecule of choice for the reconstruction of paleoenvironment. Long chain *n*-alkanes, major components of terrestrial plant leaf waxes^1^, are resistant to microbial degradation^2^, thus their molecular integrity and original isotopic compositions are well preserved over the geological timescale^3^. Leaf wax *n*-alkanes are also widely distributed in both terrestrial and marine sediments^4^,^5^, making them common biomarkers for the reconstruction of paleoclimate and paleohydrology^6^,^7^,^8^,^9^,^10^,^11^.

Previous studies have shown that leaf wax δD_*n*-alkane_ values in modern plants or sediments are closely correlated to precipitation δD^12^,^13^,^14^,^15^,^16^,^17^. However, grown under the same environmental condition with the use of the same precipitation water, different plants exhibit various δD_*n*-alkane_ values in their leaf wax *n*-alkanes. Liu *et al.*^15^ explained the difference as an effect of plant ecology and proposed that plant life forms (e.g., grasses, shrubs and trees) have a major influence on leaf wax δD_*n*-alkane_ values. They reported a global database (n = 233) of modern leaf wax δD_*n*-alkane_ values that displayed distinguished distribution patterns between woody plants (shrubs and trees) and herbaceous plants (including grasses, herbs, forbs etc.), with herbaceous plants having D-depleted by 30–50‰ relative to woody plants^16^. Subsequently, similar results among a variety of plant life forms such as trees, shrubs, grasses, herbs, forbs, vines, ferns, and aquatic plants, were observed in specific sites in middle latitudes in North America^18^, at high latitudes^19^, as well as at the global scale based upon a larger database (n = 300)^10^.

However, a critical question remains unanswered: Why the differences in leaf wax δD_*n*-alkane_ values among plant life forms occurred, and more specifically, what is the underlying cause for the observed differences in leaf wax δD_*n*-alkane_ values among different plant life forms? Liu *et al.*^15^ considered that the different source water utilized by plants might result in a greater D-enrichment in woody plants, which have more extensive root systems to tap into deeper D-enriched soil water. Hou *et al.*^18^ suggested that different abilities of evapotranspiration might be an important factor leading to less negative leaf wax δD_*n*-alkane_ values in woody plants relative to grasses. Other studies attributed the difference to other environment factors such as temperature, relative humidity, and light etc.^14^,^18^,^20^,^21^. Nevertheless, it has been proposed that the variability in lipid δD values is controlled by both environmental and biological factors^22^. Yakir and DeNiro^23^ implied that biosynthetic fractionation during the lipid formation remained to be constant for specific species, and similar conclusion was also reached across different plant groups^24^,^25^,^26^. However, Kahmen *et al.*^27^,^28^ recently found that biosynthetic fractionation may vary up to 60‰ among different species.

Therefore, to date it is not clear whether leaf wax δD_*n*-alkane_ values from modern plants were really related to plant taxonomy or simply affected by different environmental factors surrounding these individual plants. Answers to these above questions are not only critical to better understanding hydrogen fractionations in higher plants, but also have broader implications for the applications of leaf wax δD_*n*-alkane_ values as a proxy for studies of paleoclimate and paleohydrology.

The majority of previous studies have demonstrated that leaf water δ^18^O values exhibited spatial isotopic gradients inside leaf blades^29^,^30^,^31^,^32^,^33^,^34^, although few study observed spatial gradients of leaf water δD values. Moreover, previous studies on δD_*n*-alkane_ of leaf waxes have all been conducted on whole leaf samples^10^,^15^,^16^,^18^, with only one exception to focus on spatial variation of leaf wax δD_*n*-alkane_ values of *Miscanthus sinensis*, a monocotyledonous species^35^. Here we sampled modern higher plants in Northwestern China to further investigate the effect of different plant taxonomies on leaf wax δD_*n*-alkane_ values. In order to explore the difference in leaf wax δD_*n*-alkane_ values among different plant taxonomic lineages, we analyzed the relationship between leaf water δD values and corresponding leaf wax δD_*n*-alkane_ values both at the whole leaf and the segmented leaf scales.

Various plants including different major plant taxonomic lineages (e.g., eudicots, monocots, gymnosperms etc.) were sampled at each site under the same environmental condition, thus with the same hydrogen source. We investigated dicotyledonous and monocotyledonous leaf blades based on their veinal structures, with monocotyledonous leaves having parallel veins whereas dicotyledonous leaves primarily having reticulate veins^29^. We also recompiled and reanalyzed published modern leaf wax δD_*n*-alkane_ data to date from various geographic locations in the Northern Hemisphere to investigate different patterns of hydrogen isotope fractionations during leaf wax biosynthesis among plant taxonomic lineages in higher plants.

## Results

**Figure 1:**
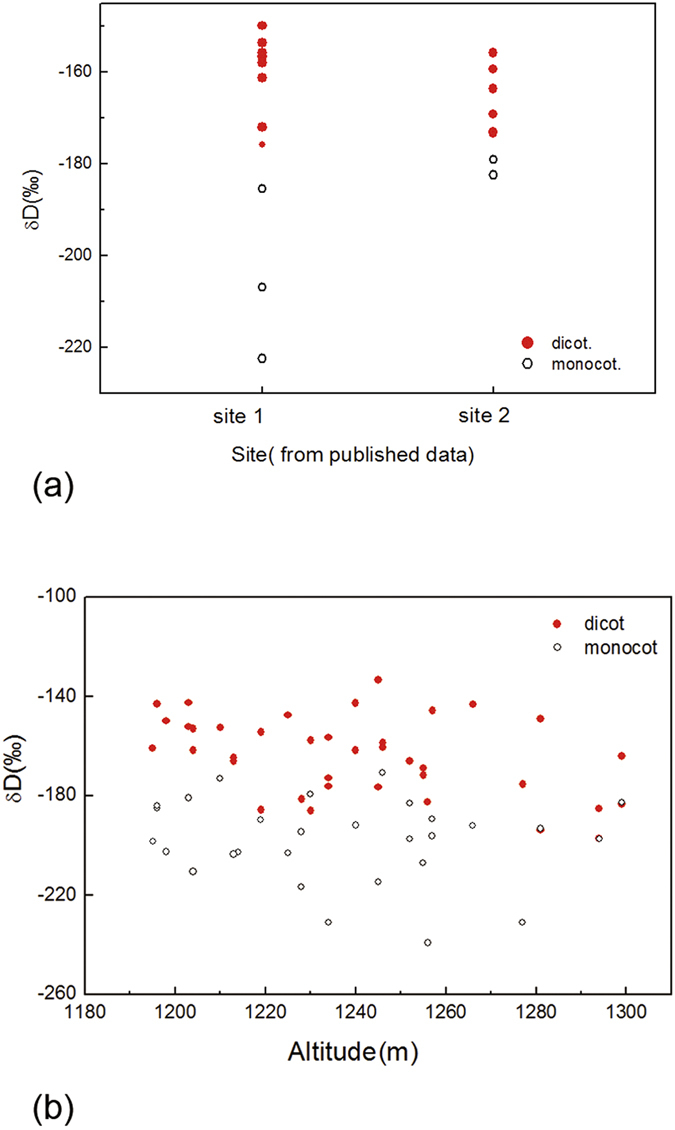
Whole leaf hydrogen isotope compositions from various sites in Northwestern China showing distinct difference in leaf wax δD_*n*-alkane_ values between dicotyledonous species and monocotyledonous species. Samples were from Lantian (site 1) and Xi’an (site 2) and from the Heshui County (b).

In general, leaf wax δD_*n*-alkane_ values in woody plants were more D-enriched than those in herbaceous plants (e.g., grasses, herbs, forbs, etc.), consistent with previous results^15^,^16^. There were relatively significant differences in leaf wax δD_*n*-alkane_ values between woody plants (shrubs and trees) and herbaceous plants (e.g., grasses, herbs, forbs, etc.) in Lantian and Xi’an^36^. However, some herbs and forbs obviously possessed higher δD_*n*-alkane_ values in leaf wax, reaching values similar to those of woody plants in the Heshui County (Supplementary Fig. S2). If we regrouped these plants according to two major plant taxonomic lineages, eudicots and monocots, we discovered that there were more distinct differences in leaf wax δD_*n*-alkane_ values between dicotyledonous and monocotyledonous species, with monocotyledonous D-depleted relative to dicotyledonous species in above three sites (Fig. 1). Leaf wax δD_*n*-alkane_ values of monocotyledonous species ranged from −171‰ to −239‰ with an average of −198‰, whereas those of dicotyledonous species exhibited a range from −133‰ to −197‰, with an average of −164% in the Heshui County (Supplementary Table S1), similar results of an average of −195‰ (monocots) and −163% (eudicots) in Xi’an and Lantian^36^.

Previous studies have implied non-woody plants (grasses, herbs, forbs, vines and some aquatic plants etc.) as grasses^10^,^15^,^16^. In this study, based on our expanded database (n = 503), we regrouped the available modern leaf wax δD_*n*-alkane_ data based upon dicotyledonous (including all woody plants of both shrubs and trees, as well as some herbs and forbs) and monocotyledonous species (grasses). Interestingly, dicotyledonous species apparently possessed more positive δD_*n*-alkane_ values than monocotyledonous grasses. One-way ANOVA test showed that the average δD_*n*-alkane_ values of leaf wax differed significantly between these two groups (*P* < 0.001), and that the average δD_*n*-alkane_ values of monocotyledonous grasses differ significantly from those of eudicot woody plants (*P* < 0.001; Table 1). However, we found no significant difference in leaf wax δD_*n*-alkane_ values between dicotyledonous species and woody plants (*P* = 0.317; Table 1) nor between shrubs and trees (*P* = 0.897)^15^. Moreover, leaf wax δD_*n*-alkane_ values of monocotyledonous species ranged from −148‰ to −301‰, with an average of −202‰, whereas leaf wax δD_*n*−alkane_ values of dicotyledonous species varied from −94‰ to −251‰, with an average of −165‰ (Table 1). Furthermore, leaf wax δD_*n*−alkane_ values in gymnosperms varied from −103‰ to −201‰, with an average of −152‰. Importantly, the ε_wax-p_ values ranged from −39‰ to −158‰ (mean value of −107‰) for dicotyledonous species and from −80‰ to −200‰ (mean value of −140‰) for monocotyledonous species. The mean ε_wax-p_ value for gymnosperms is −106‰.

## Discussion

### Plant taxonomy and the alternative hypothesis

Liu *et al.*^15^ proposed that leaf wax δD_*n*-alkane_ values were correlated with plant life forms (i.e., trees, shrubs, and grasses). Subsequently, Liu and Yang^16^ compiled and analyzed leaf wax δD_*n*-alkane_ values from modern plants across the Northern Hemisphere, and observed distinguishable distribution patterns of δD_*n*-alkane_ between woody plants and herbaceous plants (grasses, herbs, forbs, etc.), with the herbaceous plants D-depleted by ca. 40‰. Surprisingly, based on their database we observed that the captured range of leaf wax δD_*n*-alkane_ values in the herbaceous plants was obviously larger than that in woody plants, with some herbaceous plants (forbs and herbs) obviously possessing higher δD_*n*-alkane_ values of leaf wax, reaching δD values similar to those of woody plants despite the fact that they grew under the same environmental condition in the same region and utilized the same hydrogen source (Supplementary Table S2). When we regrouped these plants according to their major taxonomic lineages (e.g., eudicots, monocots and gymnosperms), we discovered that the plant taxonomy underpins such correlations at a global scale. Therefore, plant taxonomic lineages have a significant effect on leaf wax δD_*n*-alkane_ values.

**Figure 2:**
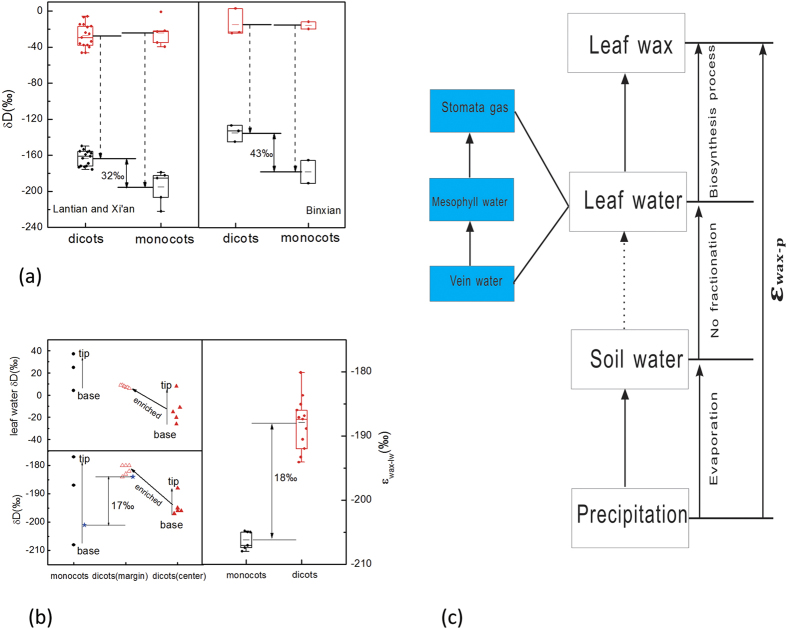
Variations in leaf water and corresponding leaf wax δD_*n*-alkane_ values. Variations of leaf water and corresponding leaf wax δD_*n*-alkane_ values on whole leaves from Xi’an, Lantian, and Binxian (**a**). Treatment on segmented leaf sections conducted from base to tip in monocotyledonous leaves and in base-to-tip and center-to-margin directions in dicotyledonous leaves, resulting difference in ε_wax-lw_ between dicotyledonous and monocotyledonous species. The difference in ε_wax-lw_ values derived from the quality-weighted mean ε_wax-lw_ values from segmented leaves was responsible for the difference in leaf wax δD_*n*-alkane_ values of whole leaf (**b**). Conceptual diagram illustrating a model of hydrogen isotope transformation in terrestrial plants. The transformation comprises two processes: D-enrichment by evapotranspiration and D-depletion by biosynthetic processes^10^. The veinal structures inside the leaves (veinal water pathways, green box) control the isotopic gradients of leaf water and corresponding leaf wax (**c**).

In order to further explore the difference in leaf wax δD_*n*-alkane_ values between dicotyledonous and monocotyledonous species, we analyzed leaf water δD values and corresponding leaf wax δD_*n*-alkane_ values both at the whole-leaf scale and at the segmented-leaf scale from representatives of these two major plant groups (Fig. 2a,b). At the whole leaf scale, no significant difference in leaf water δD values was observed between dicotyledonous and monocotyledonous species, while the corresponding leaf wax δD_*n*-alkane_ values differed up to 43‰ (Binxian, Supplementary Table S4) and 32‰ (Xi’an and Lantian, Supplementary Table S5) (Fig. 2a), similar to the result obtained by Liu *et al*^15^. Therefore, the data indicate that the difference in leaf wax δD_*n*-alkane_ values between dicotyledonous and monocotyledonous species dose not result from the D-enriched leaf water by evapotranspiration or other environmental factors (temperature, relative humidity, light, etc.). Grieu *et al.*^37^ observed that soil water δD values increased with depths whereas Kahmen *et al.*^28^ found the opposite results, suggesting that the difference of soil water utilized by plants may not be the main cause for the observed difference in leaf wax δD_*n*-alkane_ values among different plant species.

Hydrogen transformation processes from soil water to leaf wax for modern plants were presented in Fig. 2c. It is commonly accepted that little isotopic fractionation during the uptake of soil water via plant roots occurs^38^. Transpiration drives leaf water toward D-enrichment relative to soil water^39^, resulting leaf water to be utilized for biosynthesis of leaf wax *n*-alkanes with a discrimination of D during the biosynthetic fractionation (Fig. 2c)^40^. Deuterium enrichment in leaf water is a cornerstone to determine leaf wax δD_*n*-alkane_ values because isotope tracer experiments have shown that leaf wax δD_*n*-alkane_ values are directly inherited from that in leaf water pools^41^. Moreover, many studies have demonstrated that leaf water exhibited spatial isotopic gradients inside leaf blades^29^,^30^,^31^,^32^. Therefore, the underlying explanation for the interspecies δD_*n*-alkane_ variation may be correlated with veinal water pathways or biosynthetic processes (biological factors) (Fig. 2c).

At the segmented-leaf scale, leaf wax δD_*n*-alkane_ values in *Hierochloe glabra* (a monocot) showed an increasing trend from base to the tip along the blade by 30‰ (Fig. 2b, Supplementary Table S3), similar to a previous result obtained from three tall grasses (*Miscanthus sinensis*, a monocot)^35^. In contrast, dicotyledonous blades of *Rheum palmatum L.* showed a progressively increase in leaf wax δD_*n*-alkane_ values from base to tip and from center to margins (Fig. 2b, Supplementary Table S3). Compared with spatial variations in leaf wax δD_*n*-alkane_ values, leaf water D-enrichment patterns inside leaf blades are similar to leaf wax spatial variations between dicotyledonous and monocotyledonous species (Fig. 2b). There was a progressive enrichment of leaf water δD values in the base-to-tip direction in monocotyledonous species, and base-to-tip and center-to-margin directions in dicotyledonous species (Fig. 2b). Previous studies have established that the isotope enrichment in ^18^O from the base to tip along the blades in monocotyledonous species^29^,^30^. Likewise, the progressively increasing enrichments in ^18^O or D in both longitudinal (along the leaf midrib) and transversal (perpendicular to the midrib) directions were also observed in dicotyledonous species^31^,^33^. Therefore, the consistent variations in leaf wax δD_*n*-alkane_ values and corresponding leaf water δD values indicate that veinal water pathways are unlikely to be the main cause for the observed difference in leaf wax δD_*n*-alkane_ values between dicotyledonous and monocotyledonous species.

Moreover, leaf wax δD_*n*-alkane_ value of whole leaves *Hierochloe glabra* (a monocot) was −201‰, and that of the whole leaf in *Rheum palmatum L.* (a dicot) was −184‰ (Fig. 2b, Supplementary Table S3), with 17‰ difference in leaf wax δD_*n*-alkane_ values, consistent with observed patterns between the two major taxonomic groups with our above analysis of monocotyledonous species being D-depleted. The quality-weighted mean ε_wax-lw_ values inside the leaf blades between leaf water and corresponding leaf wax in dicotyledonous and monocotyledonous species were showed in Table S3. The biosynthetic fractionation between leaf water and leaf wax (ε_wax-lw_) in monocotyledonous blades were from −205‰ to −208‰ with a quality-weighted average of −206‰, while the ε_wax-lw_ value from base to the tip along the margin of a dicotyledonous leaf was quality-weighted average of −189‰, and that along the center was quality-weighted average of −187‰, with an overall quality-weighted average of −188‰ in dicotyledonous blade (Supplementary Table S3). The difference in average biosynthetic fractionation ε_wax-lw_ values between dicotyledonous and monocotyledonous species was 18‰, consistent with the difference in leaf wax δD_*n*-alkane_ values of whole leaves (Fig. 2b). Therefore, we believe that the different ε_wax-lw_ values between dicotyledonous and monocotyledonous species is the primary cause for the difference in leaf wax δD_*n*-alkane_ values between the two major angiosperm divisions.

The different biosynthetic fractionation factors (ε_wax-lw_) between the major plant lineages (eudicots versus monocots) may derive from different hydrogen sources during lipid synthesis. Three potential sources of isotopic variability in lipid hydrogen have been identified: (1) isotope effects of water (including exchange of organic H with H_2_O) associated with biosynthetic reactions, (2) the isotopic composition of biosynthetic precursors (acetate and acetyl-CoA), and (3) the isotopic composition of added agents such as NADPH during biosynthesis^24^. About 25% of the total hydrogen in *n*-alkanes is obtained directly from water, 25% of that from biosynthetic precursors, and one-half supplied by NADPH^42^. A detailed analysis on the mean δD values of the hydrogen sources was ca. +30‰ for water, ca. −70‰ for carbohydrate, and ca. −250‰ for NADPH^43^. Biosynthetic fractionation ε_wax-lw_ integrates a suite of biochemical fractionations during the biosynthesis of leaf wax *n*-alkanes; in particular, NADPH-derived hydrogen additions seem to be an important process because NADPH-derived hydrogen is strongly depleted relative to biosynthetic water. However, the isotope compositions of water and biosynthetic precursors should be the same when various plants grow at the same site. Therefore, NADPH-derived hydrogen additions may determine biosynthetic fractionation factor, ε_wax-lw_^27^, which lead to different leaf wax δD_*n*-alkane_ distribution patterns between dicotyledonous/gymnosperms and monocotyledonous species. Sternberg *et al.*^44^,^45^ concluded that biological factors appeared to be as important as environmental factors in determining hydrogen isotope ratios of plant cellulose among plants with different photosynthetic pathways (C_3_, C_4_ and CAM).

Furthermore, the different biosynthetic fractionations (ε_wax-lw_) between dicotyledonous and monocotyledonous species may also be due to their different leaf morphologies and different growth strategies^27^,^28^,^46^. In dicotyledonous species, leaf waxes form during the brief period of leaf expansion, with the majority of leaf-wax formation subsiding after leaf expansion^47^. Monocotyledonous leaves, in contrast, grow from a basal intercalary meristem and wax development throughout the day and lifetime of the leaf^47^. Our data at the segmented-leaf scale have shown that spatial variations in leaf water and leaf wax δD values, with monocotyledonous leaf having base-to-tip D-enrichment, whereas dicotyledonous leaf having base-to-tip and center-to-margin D-enrichment. Monocotyledonous leaf develops using a D-depleted source pool from a basal intercalary meristem at the base of the leaf. Nevertheless, in dicots the evaporative D-enrichment of leaf water in developing leaves can be in the same range as in fully matured leves^46^. So the two separate mechanisms for growth strategies may also produce the different biosynthetic fractionations (ε_wax-lw_) between dicotyledonous and monocotyledonous species.

Additionally, the leaf wax δD_*n*-alkane_ distribution pattern in gymnosperms is similar to that in dicotyledonous species (Supplementary Fig. S3). Moreover, the ε_wax-p_ values between leaf wax δD_*n*-alkane_ values and precipitation δD values in gymnosperm are also similar to those of dicotyledonous species. The fact that dicotyledonous species and gymnosperms have different leaf architectures and different vein structures (veinal water pathways) but have similar ε_wax-p_ values would also support the notion that evaporation due to different leaf physiognomy is unlikely the cause for the differences in leaf wax δD_*n*-alkane_ values. Therefore, we hypothesized that the biosynthetic hydrogen isotope fractionation during lipid synthesis in gymnosperm is similar to dicotyledonous species.

### Global plant taxonomic lineages (eudicots versus monocots)

**Figure 3:**
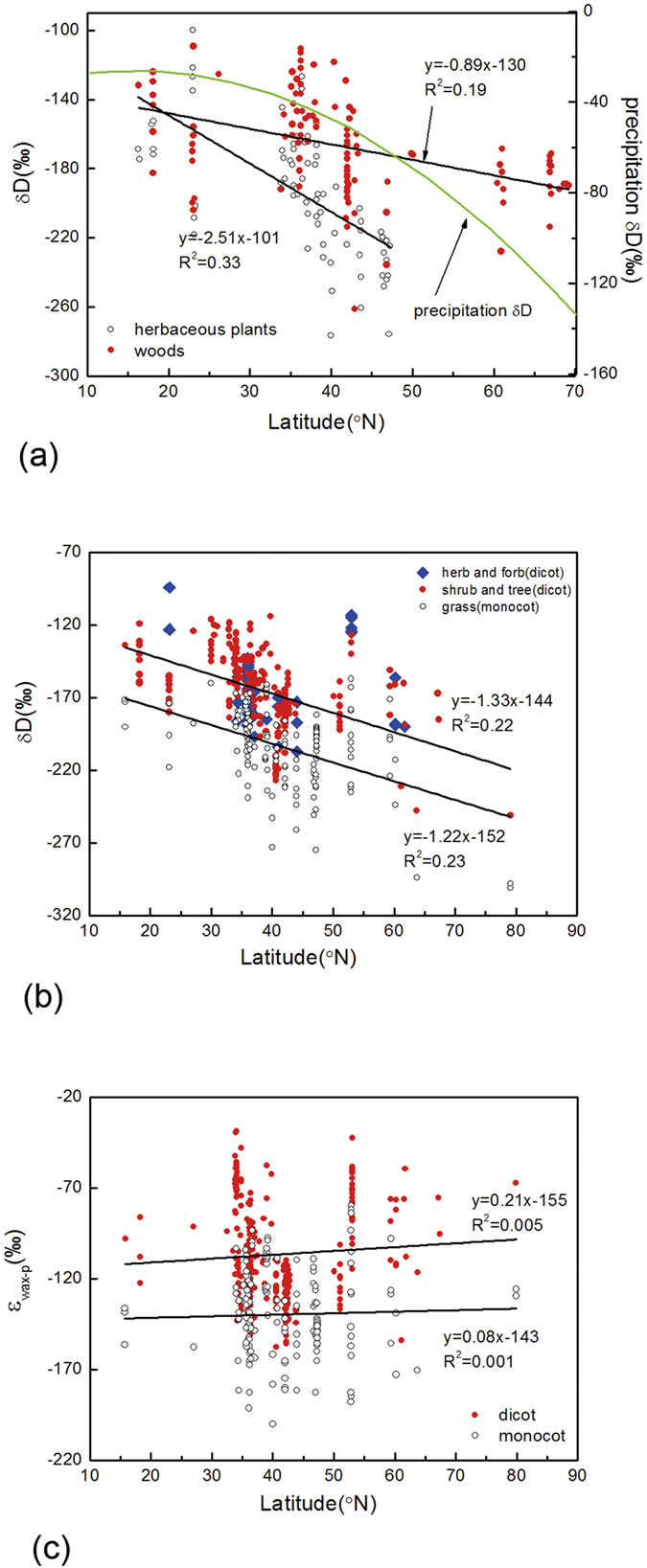
Leaf wax δD_*n*-alkane_ values in living angiosperms along a latitudinal gradient in the North Hemisphere. Comparisons of leaf wax δD_*n*-alkane_ values (n = 233) between herbaceous plants and woody plants, and the precipitation average δD were presented as δD = −0.037 × (latitude)^2^ + 1.1674 × (latitude) −35.423 from Liu and Yang (2008) (**a**). Comparisons between monocotyledonous and dicotyledonous species based on our expanded database (n = 503). Notice, the observed higher δD_*n*-alkane_ values in Stiffkey saltmarsh (Eley *et al.*, 2014) may be due to the fact that samples were obtained from a salt marsh (**b**). The parallel ε_wax-p_ values in dicotyledonous and monocotyledonous species are insensitive to variations of precipitation δD values, suggesting different biosynthetic processes between eudicots and monocots (**c**).

Liu and Yang^16^ analyzed leaf wax δD_*n*-alkane_ values from modern plants across the Northern Hemisphere and observed different distribution patterns between woody plants and herbaceous plants, with herbaceous plants being D-depleted by ca. 40‰ (Fig. 3a). Applying our expanded database, we regrouped plant taxa based upon major angiosperm divisions (eudicots and monocots) and observed distinguishable distribution patterns in leaf wax δD_*n*-alkane_ values between the two groups, with monocotyledonous species having lower δD_*n*-alkane_ values (Fig. 3b). We found that most of the herbaceous plants (mainly forbs and herbs) with higher δD_*n*-alkane_ values in Liu and Yang^16^ database in fact belong to eudicot species. When we regroup these previous outliers based on plant taxonomy rather than plant life forms, the division of leaf wax δD_*n*-alkane_ values between the two major plant groups become more distinguished (Fig. 3a,b; Supplementary Fig. S4). In general, leaf wax δD_*n*-alkane_ values of both dicotyledonous and monocotyledonous species decreased with the increase of latitudes, responding to the latitudinal effect of precipitation δD in the Northern Hemisphere. Interestingly, the rates of decreased variations in leaf wax δD_*n*-alkane_ values of both monocotyledonous and dicotyledonous species followed approximate similar slopes (Fig. 3b). The parallel variation lines would suggest that region-specific environmental factors are not the cause for the observed difference in leaf wax δD_*n*-alkane_ values between the two major taxonomic lineages, the inherent biosynthetic isotope fractionations of plant taxonomy may act as the force driving the differences.

Recently, Kahmen *et al.*^27^ observed that the full extent of leaf water evaporative D-enrichment was recorded in leaf wax δD_*n*-alkane_ values for dicotyledonous species, whereas only 18–68‰ of the D-enrichment in leaf water was recorded in leaf wax δD_*n*-alkane_ values for monocotyledonous species. While Kahmen *et al.*^27^ attributed the difference in evaporative D-enrichment of leaf water as being recorded in leaf wax δD_*n*-alkane_ values, the different biological behaviors between dicotyledonous and monocotyledonous species can also be explained by the underlying different biosynthetic processes. Therefore, our data suggest that the difference in leaf wax δD_*n*-alkane_ values between eudicot and monocot species is primarily driven by different ε_wax-lw_ values correlated with biological factors of the two major plant groups.

### Implications for the reconstruction of global precipitation

Previous studies have demonstrated a strong correlation between leaf wax δD_*n*-alkane_ values in modern plants (or sediments) and precipitation δD values both at regional and global scales^13^,^15^,^16^,^17^. Precipitation δD value is a dominating factor that exercises the first order of control for plant leaf wax δD_*n*-alkane_ values along the latitudes^16^. Our new data confirm that the ε_wax-p_ values between leaf wax δD_*n*-alkane_ values of major plant lineages (eudicots versus monocots) and precipitation δD values exhibited distinguishable distribution patterns at a global scale, with monocotyledonous species having larger ε_wax-p_ values than dicotyledonous species (Fig. 3c). The ε_wax-p_ values vary ranging from −39‰ to −158‰ for dicotyledonous species and from −80‰ to −200‰ for monocotyledonous species. The mean ε_wax-p_ value is −107‰ for dicotyledonous species (−106‰ for gymnosperms) and −140‰ for monocotyledonous species, similar to the results of −113‰ for C_3_ eudicots and −149‰ for C_3_ monocots^13^. However, the parallel ε_wax-p_ patterns along with the latitudes suggest that different biosynthetic processes between dicotyledonous and monocotyledonous species are insensitive to regional variations of precipitation δD values (Fig. 3c), further supporting the notion that the underlying difference in leaf wax δD_*n*-alkane_ values between dicotyledonous and monocotyledonous species is due to different hydrogen fractionations during biosynthetic processes.

As different mean ε_wax-p_ values (−107‰ for eudicots/ gymnosperms; −140‰ for monocots) corresponds to different biosynthetic processes with different biosynthetic fractionation ε_wax-lw_ values in different major plant lineages, plant taxonomy does play an important role in controlling leaf wax δD_*n*-alkane_ values in higher plants. Our data imply that (1) we must distinguish vegetation change signals form environmental variation signals during the reconstruction of paleoenvironment, (2) the previous practice of using a taxonomic blind average ε_wax-p_ value to reconstruct paleo-precipitation δD values without considering the composition of vegetation may lead to large errors.

## Conclusions

Our reanalysis of an expanded database of available leaf wax δD_*n*-alkane_ records from modern higher plants revealed that the differences in leaf wax δD_*n*-alkane_ values among different plant life forms (e.g., grasses, shrubs, trees, herbs, forbs, veins, ferns and aquatic plants etc.) are in fact underpinned by the plant taxonomic division of major systematic lineages, with monocotyledonous species being D-depleted relative to dicotyledonous species. This alternative interpretation explains the previous observed D-enrichment δD_*n*-alkane_ values of leaf wax from some outlier eudicot herbaceous samples in a given site and improved global correlations between leaf wax δD_*n*-alkane_ values and precipitation δD. Our data further suggest that biological factors, such as different hydrogen additions (NADPH-derived) for lipid synthesis, play key roles in determining different hydrogen isotope fractionations under different plant lineages. Additionally, the different growth strategies between dicotyledonous and monocotyledonous leaves may also produce different biosynthetic fractionation factors (ε_wax-lw_). Future studies may focus on detailed biochemical process and molecular mechanism that lead to distinguished hydrogen biosynthetic fractionations among major taxonomic lineages in higher plants. The recognition of various ε_wax-p_ values between different plant taxonomic lineages imply that using average ε_wax-p_ values without the knowledge of vegetation may lead to large errors for the reconstruction of ancient precipitation δD values. Our findings further confirm that it is imperative to take this “plant taxonomy effect” into account when employing δD_*n*-alkane_ values of leaf wax as a paleo-hydrology proxy in areas where vegetation change has been pronounced.

## Materials and Methods

**Figure 4:**
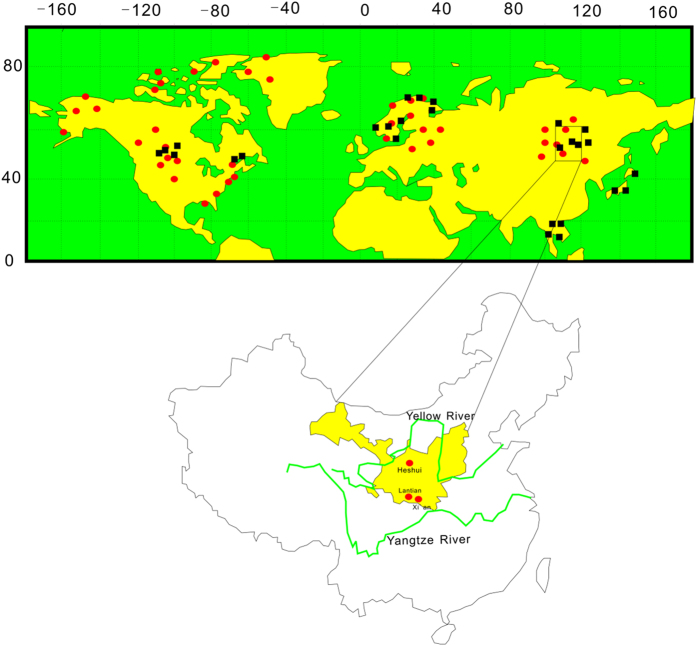
Geographic map showing sample sites in Heshui, Lantian and Xi’an and the distribution of compiled modern leaf wax δD_*n*-alkane_ values of higher plants across the North hemisphere. Black rectangles are data based upon Liu and Yang, (2008); red dots are additional data points. (Fig. 4 was created by CorelDRAW 12; My co-authors and I grant NPG to publish the image under an Open Acess license; We grant NPG to publish the image in all formats i.e. print and digital).

Modern higher plants were sampled in Northwestern China and a larger dataset of available δD_*n*-alkane_ data that was previously published from various regions in the Northern Hemisphere were compiled (Fig. 4; Supplementary Table S1). For the whole leaf studies, we sampled 68 plants from Heshui County in May and September of 2013 and some published δD_*n*-alkane_ data from Xi’an and Lantian were used in this analysis^36^. To ensure a complete representation of the whole leaf signal, we collected intact leaves on the account of likely leaf water isotopic gradients along the leaf blade^29^.

In order to investigate isotopic gradients inside leaf blades, we sampled *Rhleum palmatum L.* (an eudicot) and *Hierochloe glabra* (a monocot) at the same site from Binxian on 19 May, 2014. About 5–7 leaves with similar sizes were collected for each species for experimental analysis. Analyses were conducted for whole leaves as well as for leaf segments. Whole leaves of both species were treated to obtain leaf water δD values and leaf wax δD_*n*-alkane_ values. The dicotyledonous blade of *Rheum palmatum L.* was cut into segments along the main veins and margins of a leaf, and the leaves of species *Hierochloe glabra* were segmented from base to the tip in order to obtain variations of leaf water δD and leaf wax δD_*n*-alkane_ values for various sections of the leaf blade (Supplementary Fig. S1). These plants grew on the Chinese Loess Plateau where they received full sun and natural rainfall without human disturbance. We collected these plant samples between 12 pm to 15 pm to capture the maximum diurnal enrichment in leaf water isotopic composition^48^. Leaf samples were immediately enclosed in 100-ml screw-cap plastic vials with Parafilm^®^ and kept in a dry cooler (ca. 4 °C) in the field until transferred to the laboratory.

Leaf water was extracted using a cryogenic vacuum distillation^49^, and leaf water δD values were determined using a L2130-I isotope water analyzer (Picarro, Sunnyvale, CA, USA) with the Micro-Combustion Module^TM^ (MCM) to remove organic compounds contaminating water samples^50^,^51^. The measurement precision for water δD analysis was 1%. After the extraction of leaf water, the leaf remains were used for leaf wax *n*-alkane extraction and hydrogen isotope measurement as described in Liu *et al.*^15^. The H_3_^+^ factor was determined daily and remained at 2.325 ± 0.008 (n = 6) during sample analysis, ensuring stable ion source conditions. A mixed laboratory standard consisting of six *n*-alkanes (C_21_, C_25_, C_27_, C_29_, C_31_ and C_33_) was analyzed between every four measurements to evaluate the reproducibility and accuracy of the instrument. The standard deviation for the *n*-alkane working standard was 3‰, and each sample was analyzed in duplicate or more, with the standard deviation usually less than 3‰. The accuracy from co-injected primary standards (*n*-alkanes available from A. Schimmelmann, Biogeochemical Laboratories, Indiana University) was ± 3‰ (n = 6). The δD values are reported relative to the Vienna mean standard ocean water (VSMOW).

In order to test the hypothesis at the global scale, we compiled the available modern leaf wax δD_*n*-alkane_ data from various locations in the Northern Hemisphere to form an expanded database (n = 503) for statistical analysis. The database included published δD_*n*-alkane_ data from China^15^,^16^, Japan and Thailand^20^, North America^14^,^18^,^39^,^47^,^52^, and European regions^53^,^54^,^55^,^56^ and some regions in the high latitudes of the Arctic^19^, spanning from 16°06′N to 79°5′N in latitude and from 0°55′E to 142°11′E and from 148°56′W to 71°58′W in longitude, including various plant life forms (Fig. 4; Supplementary Table S2).

Pearson correlations were conducted to investigate various correlations between δD_*n*-alkane_ values of plant taxonomies and various geographic and environmental factors. One-way ANOVA tests were used to identify differences among factors at *P* = 0.001. Student’s *t-tests* were used to identify differences between two factors. The hydrogen isotopic fractionation factor (ε_wax-lw_) between leaf wax *n*-alkane (δD_wax_) and corresponding leaf water (δD_lw_) is calculated as:


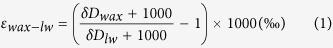


To quantify the influence of precipitation δD values on leaf wax δD_*n*-alkane_ values, the apparent hydrogen fractionation factor (ε_wax-p_) between leaf wax and precipitation is determined as the following:


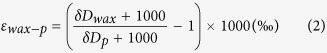


## Additional Information

**How to cite this article**: Liu, J. *et al.* Different hydrogen isotope fractionations during lipid formation in higher plants: Implications for paleohydrology reconstruction at a global scale. *Sci. Rep.* **6**, 19711; doi: 10.1038/srep19711 (2016).

## Supplementary Material

Supplementary Information

## Figures and Tables

**Table 1:** Results of statistical analysis based on our expanded database (n = 503).

Category	Type	Mean(‰)	sd	*P*-values		
	herbaceous plants	−192	32	grasses(monocots)		
plant life forms					P < 0.001	
	shrubs and trees	−168	30			
				forbs and herbs(dicots)		P < 0.001
	dicots	−167	29			
plant taxonomies					P = 0.317	
	monocots	−202	28	shrubs and trees(dicots)		

Values are the mean of different life forms or plant taxonomic lineages, sd = standard deviation of the mean. Statistical differences in δD among types were calculated using a one-way ANOVA. *P*-values of these analyses are presented in the table.
