# Supplementary material for: Different hydrogen isotope fractionations during lipid formation in higher plants: Implications for paleohydrology reconstruction at a global scale
Source: Sci Rep. 2016 Jan 25;6:19711. doi: 10.1038/srep19711 (PMC4726312; doi:10.1038/srep19711)

**Supporting information for**

# Different hydrogen isotope fractionations during lipid formation in higher plants: Implications for paleohydrology reconstruction at a global scale

Jinzhao Liu[1,2], Weiguo Liu[1,2*], Zhisheng An[1] & Hong Yang[3*]

[1] State Key Laboratory of Loess and Quaternary Geology, IEE, CAS, Xi'an 710075, China

[2] School of Human Settlements and Civil Engineering, Xi'an Jiaotong University, Xi'an 710049, China

[3] Laboratory for Terrestrial Environments, College of Arts and Sciences, Bryant University, Smithfield, RI 02917, USA

**[*]Corresponding author**

Weiguo Liu

Institute of Earth Environment, Chinese Academy of Sciences

Feihui Road, 10#, Xi'an High-Tech Zone

710075

Tel: +862988323495

E-mail: liuwg@loess.llqg.ac.cn

Hong Yang

Tel.: +14012326223

E-mail address: hyang@bryant.edu

## Supporting information legends

*Appendix A-The relationship between segmented leaves and whole leaf.*

**Table S1** Plant leaf wax $\delta D_{n\text{-alkane}}$ values sampled from Northwestern China.

**Table S2** Table S2 Data sources, distribution, taxonomy, and references of plant leaf wax $\delta D_{n\text{-alkane}}$ values compiled from the Northern Hemisphere and used in this study.

**Table S3** Measured δD values of leaf water and leaf wax along the leaf blade in *Rhleum palmatum L.* (dicot) and *Hierochloe glabra* (monocot) (‰, VSMOW).

**Table S4** The measured and calculated hydrogen isotope composition of leaf water ($\delta D_{\text{leaf water}}$), leaf wax ($\delta D_{C29\ n\text{-alkane}}$) and corresponding $\varepsilon_{\text{wax-lw}}$ values from segmented leaves and entire leaf (‰).

**Table S5** The δD values of leaf water and corresponding leaf wax *n*-alkane in Xi'an and Lantian, China (‰).

**Fig. S1.** The size of the leaf blade of Rhleum palmatum L. (dicot) and Hierochloe glabra (monocot). The two species were adjacent to grow on the Chinese Loess Plateau where they received full sun and natural rainfall, without human disturbance.

**Fig. S2** Leaf wax $\delta D_{n\text{-alkane}}$ values between woods (shrubs and trees) and herbaceous plants from Lantian and Xi'an (Liu et al., 2015) and from Heshui County (site 3). Apart from Lantian and Xi'an, no significant difference in leaf wax $\delta D_{n\text{-alkane}}$ values between woods and herbaceous plants in Heshui County was observed.

**Fig. S3** Leaf wax $\delta D_{n\text{-alkane}}$ and $\varepsilon_{\text{wax-p}}$ values in gymnosperms varied along the latitude.

**Fig. S4** Leaf wax $\delta D_{n\text{-alkane}}$ values between eudicots and monocots in herbaceous plants collected from the North Hemisphere. One-way ANOVA test showed that the average $\delta D_{n\text{-alkane}}$ values of leaf wax differed significantly between monocotyledonous and dicotyledonous herbaceous plants ($P < 0.001$).

**Fig. S5**. Showing leaf segmented sections where dicotyledonous blade in *Rheum palmatum L.* was cut into sections from base to tip along the main veins and edges of blade, whereas monocotyledonous blades in *Hierochloe galbra* from base to tip. The $\varepsilon_{\text{wax-lw}}$ values between leaf wax and leaf water in every segmented sections varied along the leaf blade and corresponding leaf water δD values in brackets (‰). The results indicated the $\varepsilon_{\text{wax-lw}}$ values inside leaf blade were constant, but differed significantly between dicotyledonous and monocotyledonous species.

**Fig. S6** Leaf wax $\delta D_{n\text{-alkane}}$ values between monocotyledonous and dicotyledonous species collected from different sampling sites in this study. Note: site 1 (Lantian: 34°14′N, 109°7′E); site 2 (Xi'an： 34°20′N, 108°95′E); site 3 (Heshui County：36°01'N, 108°06'N); site 4 (Luochuan：35°49′N, 109°30′E); site 5 (Yuxian：37°05′N, 113°02′E); site 6 (Ruicheng：34°44′N, 110°25′E); site 7 (Ertuokeqi：39°05′N, 107°58′E); site 8 (Yanan：36°35′N, 109°29′E); site 9 (Huining, Baishui, Neimeng, Pengyang, Jitantai, Yanchi, Yijun, Yingchuan, Lanzhou etc.); site 10 (Axel Heiberg Island：79°54′N, 89°01′W); site 11 (Denali：63°43′N, 148°54′W); site 12 (Helsinki：60°10′N, 24°56′E); site 13 (Stockholm：59°20′N, 18°02′E); site 14 (Gunma-Japan： 36°23′N, 139°37′E); site 15 (Tainland：15°52′N, 100°59′E); site 16 (Tokyo-Japan：35°41′N, 139°30′E); site 17 (Blood pond：42°08′N, 71°96′W); site 18 (Stiffkey：52°58′N, 0°55′E).

*Appendix A-The relationship between segmented leaves and whole leaf.*

In order to explore the relationship between segmented leaves and corresponding entire leaf, we collected leaf samples selected monocotyledonous and dicotyledonous species on 19 May 2014 in Binxian county (35 ° 14' N, 108 ° 9' E), Shannxi Province, China. We randomly separated these leaves of the same species into two groups. The one group for entire leaf analysis which was treated to obtain leaf water δD values and leaf wax $\delta D_{n\text{-alkane}}$ value, the other was for segmented leaf analysis of the same species as described in the main text. The leaves of dicotyledonous species were cut into segments from base to tip and from center to edge, whereas those of monocotyledonous leaves were segmented from base to tip (Fig. 1). To link the segmented leaves to the entire leaf, we first determined quality-weighted mean leaf wax $\delta D_{n\text{-alkane}}$ and $\varepsilon_{wax\text{-}lw}$ values from segmented leaves to obtain calculated $\delta D_{n\text{-alkane}}$ and $\varepsilon_{wax\text{-}lw}$ values for each species (Table S2). These calculated $\delta D_{n\text{-alkane}}$ and $\varepsilon_{wax\text{-}lw}$ values were then compared with the measured leaf wax $\delta D_{n\text{-alkane}}$ and $\varepsilon_{wax\text{-}lw}$ values (Fig. 2). Clearly, the quality-weighted mean leaf wax $\delta D_{n\text{-alkane}}$ and $\varepsilon_{wax\text{-}lw}$ values from segmented leaves predict leaf wax $\delta D_{n\text{-alkane}}$ and $\varepsilon_{wax\text{-}lw}$ values of entire leaf.

Therefore, the relationship between leaf wax $\delta D_{n\text{-alkane}}$ and $\varepsilon_{wax\text{-}lw}$ values from segmented leaves and leaf wax $\delta D_{n\text{-alkane}}$ and $\varepsilon_{wax\text{-}lw}$ values of entire leaf can be linked by quality-weighted mean equations. In our study the calculated $\varepsilon_{wax\text{-}lw}$ values derived from the quality-weighted mean $\varepsilon_{wax\text{-}lw}$ values from segmented leaves is reasonable for the representative of the entire $\varepsilon_{wax\text{-}lw}$ values.

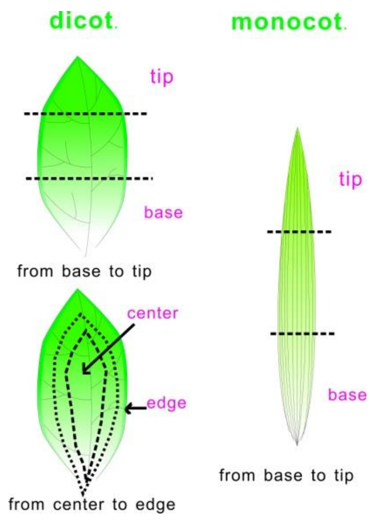


Fig. 1 Showing leaf cuts from base to tip and from center to edge in dicotyledonous species, whereas those of monocotyledonous leaves were segmented from base to tip.

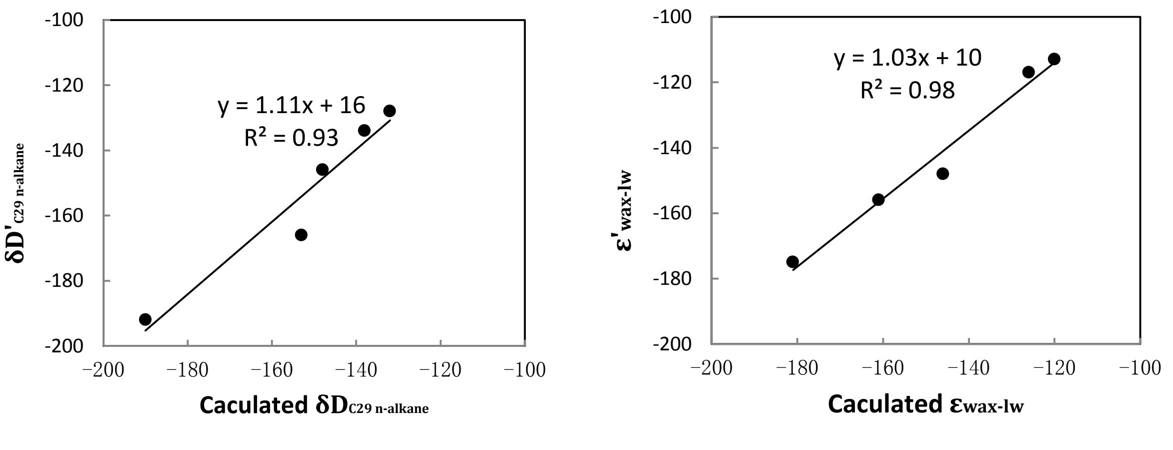


Fig. 2 The comparison between the quality-weighted mean $\delta D_{n\text{-alkane}}$ and $\varepsilon_{wax\text{-}lw}$ values from segmented leaves and measured $\delta D'_{C29\ n\text{-alkane}}$ and $\varepsilon'_{wax\text{-}lw}$ values from entire leaf.

Table S1 Plant leaf wax δ$D_{n\text{-alkane}}$ values sampled from Northwestern China.

| Sample ID | Sample date | Species | Location | Latitude | Longitude | Altitude/m | Family | Class | Plant life forms | Photosynthetic pathway | δ$D_{C29\ n\text{-alkane}}$(‰) |
|---|---|---|---|---|---|---|---|---|---|---|---|
| LH-1 | Sept.(2012) | *Bothriochloa ischaemum* | Lantian | 34°14′ | 109°7′ | 619 | Poaceae | Monocotyledoneae | grass | C4 | -207 |
| LH-12 | Sept.(2012) | *Stipa bungeana* | Lantian | 34°25′ | 109°18′ | 619 | Poaceae | Monocotyledoneae | grass | C3 | -222 |
| LH-13 | Sept.(2012) | *Agropyron cristatum* | Lantian | 34°25′ | 109°18′ | 619 | Poaceae | Monocotyledoneae | grass | C3 | -185 |
| LH-2 | Sept.(2012) | *Artemisia* | Lantian | 34°15′ | 109°8′ | 619 | Asteraceae | | grass | C3 | -224 |
| LH-3 | Sept.(2012) | *no identification* | Lantian | 34°16′ | 109°9′ | 619 | Asteraceae | Dictyledoneae | shrub | | -158 |
| LH-4 | Sept.(2012) | *Lespedeza davurica* | Lantian | 34°17′ | 109°10′ | 619 | Leguminosae | Dictyledoneae | shrub | C3 | -172 |
| LH-5 | Sept.(2012) | *Populus adenopoda* | Lantian | 34°18′ | 109°11′ | 619 | Salicaceae | Dictyledoneae | tree | C3 | -154 |
| LH-6 | Sept.(2012) | *Populus adenopoda* | Lantian | 34°19′ | 109°12′ | 619 | Salicaceae | Dictyledoneae | tree | C3 | -157 |
| LH-7 | Sept.(2012) | *Populus adenopoda* | Lantian | 34°20′ | 109°13′ | 619 | Salicaceae | Dictyledoneae | tree | C3 | -161 |
| LH-8 | Sept.(2012) | *Populus adenopoda* | Lantian | 34°21′ | 109°14′ | 619 | Salicaceae | Dictyledoneae | tree | C3 | -150 |
| LH-9 | Sept.(2012) | *no identification* | Lantian | 34°22′ | 109°15′ | 619 | | Dictyledoneae | tree | C3 | -156 |
| LH-10 | Sept.(2012) | *no identification* | Lantian | 34°23′ | 109°16′ | 619 | | Dictyledoneae | tree | C3 | -172 |
| LH-11 | Sept.(2012) | *no identification* | Lantian | 34°24′ | 109°17′ | 619 | | Dictyledoneae | tree | C3 | -176 |
| ZWY10-1-36 | | *Agrostis matsumurae* | Xi'an | 34°20′ | 108°95′ | 384 | Poaceae | Monocotyledoneae | grass | C3 | -179 |
| ZWY10-2-25 | | *Lolium perenne* | Xi'an | 34°20′ | 108°95′ | 384 | Poaceae | Monocotyledoneae | grass | C3 | -182 |
| ZWY10-1-34 | | *Buddleja alternifolia* | Xi'an | 34°20′ | 108°95′ | 384 | Buddlejaceae | Dictyledoneae | shrub | C3 | -159 |
| ZWY10-1-37-2 | | *Cirsium setosum* | Xi'an | 34°20′ | 108°95′ | 384 | Asteraceae | Dictyledoneae | herb | C3 | -173 |
| ZWY10-2-24 | | *Potentilla kleiniana* | Xi'an | 34°20′ | 108°95′ | 384 | Rosaceae | Dictyledoneae | herb | C3 | -156 |
| ZWY10-1-33 | | *Cerasus yedoensis* | Xi'an | 34°20′ | 108°95′ | 384 | Rosaceae | Dictyledoneae | tree | C3 | -173 |
| ZWY10-1-35 | | *Swida macrophylla* | Xi'an | 34°20′ | 108°95′ | 384 | Cornaceae | Dictyledoneae | tree | C3 | -169 |
| ZWY10-2-23 | | *Ligustrum compactum* | Xi'an | 34°20′ | 108°95′ | 384 | Oleaceae | Dictyledoneae | tree | C3 | -164 |
| HSP13-1 | May(2013） | *Stipa bungeana* | Heshui | 36°01' | 108°06' | 1299 | Poaceae | Monocotyledoneae | grass | C3 | -183 |
| HSP13-11 | May(2013） | *Stipa bungeana* | Heshui | 36°01' | 108°06' | 1252 | Poaceae | Monocotyledoneae | grass | C3 | -183 |
| HSP13-13 | May(2013） | *Bothriochloa ischaemum* | Heshui | 36°01' | 108°06' | 1252 | Poaceae | Monocotyledoneae | grass | C4 | -197 |
| HSP13-14 | May(2013） | *Stipa bungeana* | Heshui | 36°01' | 108°06' | 1246 | Poaceae | Monocotyledoneae | grass | C3 | -171 |
| HSP13-17 | May(2013） | *Stipa bungeana* | Heshui | 36°01' | 108°06' | 1230 | Poaceae | Monocotyledoneae | grass | C3 | -179 |
| HSP13-21 | May(2013） | *Stipa bungeana* | Heshui | 36°01' | 108°06' | 1210 | Poaceae | Monocotyledoneae | grass | C3 | -173 |
| HSP13-23 | May(2013） | *Stipa bungeana* | Heshui | 36°01' | 108°06' | 1203 | Poaceae | Monocotyledoneae | grass | C3 | -181 |
| HSP13-27 | May(2013） | *Stipa bungeana* | Heshui | 36°01' | 108°06' | 1196 | Poaceae | Monocotyledoneae | grass | C3 | -185 |
| HSP13-30 | May(3013） | *Bothriochloa ischaemum* | Heshui | 36°01' | 108°06' | 1257 | Poaceae | Monocotyledoneae | grass | C4 | -189 |
| HSP13-32 | May(3013） | *Stipa bungeana* | Heshui | 36°01' | 108°06' | 1257 | Poaceae | Monocotyledoneae | grass | C3 | -196 |
| HSP13-38 | May(3013） | *Bothriochloa ischaemum* | Heshui | 36°01' | 108°06' | 1240 | Poaceae | Monocotyledoneae | grass | C4 | -192 |
| HSP13-39 | May(3013） | *Bothriochloa ischaemum* | Heshui | 36°01' | 108°06' | 1225 | Poaceae | Monocotyledoneae | grass | C4 | -203 |
| HSP13-41 | May(3013） | *Bothriochloa ischaemum* | Heshui | 36°01' | 108°06' | 1214 | Poaceae | Monocotyledoneae | grass | C4 | -202 |
| HSP13-43 | May(3013） | *Bothriochloa ischaemum* | Heshui | 36°01' | 108°06' | 1198 | Poaceae | Monocotyledoneae | grass | C4 | -202 |
| HSP13-47 | May(3013） | *Stipa capillata* | Heshui | 36°01' | 108°06' | 1195 | Poaceae | Monocotyledoneae | grass | C3 | -198 |
| HSP13-48 | Sept.(2013) | *Phragmites australis* | Heshui | 36°01' | 108°06' | 1256 | Poaceae | Monocotyledoneae | grass | C3 | -239 |
| HSP13-5 | May(2013） | *Stipa bungeana* | Heshui | 36°01' | 108°06' | 1281 | Poaceae | Monocotyledoneae | grass | C3 | -193 |
| HSP13-50 | Sept.(2013) | *Carex* | Heshui | 36°01' | 108°06' | 1245 | Cyperaceae | Monocotyledoneae | grass | C3 | -215 |
| HSP13-54 | Sept.(2013) | *Phragmites australis* | Heshui | 36°01' | 108°06' | 1234 | Poaceae | Monocotyledoneae | grass | C3 | -231 |
| HSP13-56 | Sept.(2013) | *Bothriochloa ischaemum* | Heshui | 36°01' | 108°06' | 1219 | Poaceae | Monocotyledoneae | grass | C4 | -190 |
| HSP13-61 | Sept.(2013) | *Phragmites australis* | Heshui | 36°01' | 108°06' | 1204 | Poaceae | Monocotyledoneae | grass | C3 | -210 |
| HSP13-63 | Sept.(2013) | *Stipa bungeana* | Heshui | 36°01' | 108°06' | 1294 | Poaceae | Monocotyledoneae | grass | C3 | -197 |
| HSP13-66 | Sept.(2013) | *Phragmites australis* | Heshui | 36°01' | 108°06' | 1277 | Poaceae | Monocotyledoneae | grass | C3 | -231 |
| HSP13-68 | Sept.(2013) | *Carex* | Heshui | 36°01' | 108°06' | 1255 | Cyperaceae | Monocotyledoneae | grass | C3 | -207 |
| HSP13-70 | Sept.(2013) | *Phragmites australis* | Heshui | 36°01' | 108°06' | 1228 | Poaceae | Monocotyledoneae | grass | C3 | -194 |
| HSP13-72 | Sept.(2013) | *Bothriochloa ischaemum* | Heshui | 36°01' | 108°06' | 1228 | Poaceae | Monocotyledoneae | grass | C4 | -217 |
| HSP13-73 | Sept.(2013) | *Bothriochloa ischaemum* | Heshui | 36°01' | 108°06' | 1213 | Poaceae | Monocotyledoneae | grass | C4 | -203 |
| HSP13-76 | Sept.(2013) | *Zea mays* | Heshui | 36°01' | 108°06' | 1196 | Poaceae | Monocotyledoneae | grass | C4 | -184 |
| HSP13-8 | May(2013） | *Stipa bungeana* | Heshui | 36°01' | 108°06' | 1266 | Poaceae | Monocotyledoneae | grass | C3 | -192 |
| HSP13-12 | May(2013） | *Lespedeza cuneata* | Heshui | 36°01' | 108°06' | 1252 | Asteraceae | Dictyledoneae | herb | C3 | -166 |
| HSP13-15 | May(2013） | *Lespedeza cuneata* | Heshui | 36°01' | 108°06' | 1246 | Asteraceae | Dictyledoneae | herb | C3 | -159 |
| HSP13-18 | May(2013） | *Lespedeza cuneata* | Heshui | 36°01' | 108°06' | 1230 | Asteraceae | Dictyledoneae | herb | C3 | -158 |
| HSP13-2 | May(2013） | *Lespedeza cuneata* | Heshui | 36°01' | 108°06' | 1299 | Asteraceae | Dictyledoneae | herb | C3 | -164 |
| HSP13-22 | May(2013） | *Lespedeza cuneata* | Heshui | 36°01' | 108°06' | 1210 | Asteraceae | Dictyledoneae | herb | C3 | -152 |
| HSP13-24 | May(2013） | *Lespedeza cuneata* | Heshui | 36°01' | 108°06' | 1203 | Asteraceae | Dictyledoneae | herb | C3 | -142 |
| HSP13-28 | May(2013） | *Lespedeza cuneata* | Heshui | 36°01' | 108°06' | 1196 | Asteraceae | Dictyledoneae | herb | C3 | -143 |
| HSP13-31 | May(3013） | *Lespedeza cuneata* | Heshui | 36°01' | 108°06' | 1257 | Asteraceae | Dictyledoneae | herb | C3 | -146 |
| HSP13-34 | May(3013） | *Lespedeza cuneata* | Heshui | 36°01' | 108°06' | 1240 | Asteraceae | Dictyledoneae | herb | C3 | -143 |
| HSP13-40 | May(3013） | *Lespedeza cuneata* | Heshui | 36°01' | 108°06' | 1225 | Asteraceae | Dictyledoneae | herb | C3 | -148 |
| HSP13-52 | Sept.(2013) | *Lespedeza cuneata* | Heshui | 36°01' | 108°06' | 1245 | Asteraceae | Dictyledoneae | herb | C3 | -176 |
| HSP13-54-1 | Sept.(2013) | *Artemisia capillaris* | Heshui | 36°01' | 108°06' | 1234 | Asteraceae | Dictyledoneae | herb | c3 | -156 |

| | | | | | | | | | | | |
|---|---|---|---|---|---|---|---|---|---|---|---|
| HSP13-55 | Sept.(2013) | *Lespedeza cuneata* | Heshui | 36°01' | 108°06' | 1234 | Asteraceae | Dictyledoneae | herb | C3 | -173 |
| HSP13-57 | Sept.(2013) | *Artemisia capillaris* | Heshui | 36°01' | 108°06' | 1219 | Asteraceae | Dictyledoneae | herb | c3 | -154 |
| HSP13-58 | Sept.(2013) | *Artemisia giraldii* | Heshui | 36°01' | 108°06' | 1219 | Asteraceae | Dictyledoneae | herb | C3 | -186 |
| HSP13-59 | Sept.(2013) | *Artemisia capillaris* | Heshui | 36°01' | 108°06' | 1204 | Asteraceae | Dictyledoneae | herb | c3 | -153 |
| HSP13-6 | May(2013） | *Lespedeza cuneata* | Heshui | 36°01' | 108°06' | 1281 | Asteraceae | Dictyledoneae | herb | C3 | -149 |
| HSP13-64 | Sept.(2013) | *Lespedeza cuneata* | Heshui | 36°01' | 108°06' | 1294 | Asteraceae | Dictyledoneae | herb | C3 | -185 |
| HSP13-65 | Sept.(2013) | *Lespedeza cuneata* | Heshui | 36°01' | 108°06' | 1277 | Asteraceae | Dictyledoneae | herb | C3 | -175 |
| HSP13-67 | Sept.(2013) | *Lespedeza cuneata* | Heshui | 36°01' | 108°06' | 1255 | Asteraceae | Dictyledoneae | herb | C3 | -169 |
| HSP13-71 | Sept.(2013) | *Lespedeza cuneata* | Heshui | 36°01' | 108°06' | 1228 | Asteraceae | Dictyledoneae | herb | C3 | -181 |
| HSP13-74 | Sept.(2013) | *Lespedeza cuneata* | Heshui | 36°01' | 108°06' | 1213 | Asteraceae | Dictyledoneae | herb | C3 | -166 |
| HSP13-75 | Sept.(2013) | *Artemisia capillaris* | Heshui | 36°01' | 108°06' | 1213 | Asteraceae | Dictyledoneae | herb | c3 | -165 |
| HSP13-9 | May(2013） | *Lespedeza cuneata* | Heshui | 36°01' | 108°06' | 1266 | Asteraceae | Dictyledoneae | herb | C3 | -143 |
| HSP13-16 | May(2013） | *Ziziphus jujuba Mill. Var. spinosa* | Heshui | 36°01' | 108°06' | 1246 | Rhamnaceae | Dictyledoneae | shrub | C3 | -161 |
| HSP13-19 | May(2013） | *Ziziphus jujuba Mill. Var. spinosa* | Heshui | 36°01' | 108°06' | 1230 | Rhamnaceae | Dictyledoneae | shrub | C3 | -186 |
| HSP13-25 | May(2013） | *Ziziphus jujuba Mill. Var. spinosa* | Heshui | 36°01' | 108°06' | 1203 | Rhamnaceae | Dictyledoneae | shrub | C3 | -152 |
| HSP13-36 | May(3013） | *Ziziphus jujuba Mill. Var. spinosa* | Heshui | 36°01' | 108°06' | 1240 | Rhamnaceae | Dictyledoneae | shrub | C3 | -162 |
| HSP13-46 | May(3013） | *Artemisia* | Heshui | 36°01' | 108°06' | 1195 | | Dictyledoneae | shrub | | -161 |
| HSP13-49 | Sept.(2013) | *Ziziphus jujuba Mill. Var. spinosa* | Heshui | 36°01' | 108°06' | 1256 | Rhamnaceae | Dictyledoneae | shrub | C3 | -182 |
| HSP13-53 | Sept.(2013) | *Ziziphus jujuba Mill. Var. spinosa* | Heshui | 36°01' | 108°06' | 1234 | Rhamnaceae | Dictyledoneae | shrub | C3 | -176 |
| HSP13-60 | Sept.(2013) | *Lespedeza bicolor* | Heshui | 36°01' | 108°06' | 1204 | Leguminosae | Dictyledoneae | shrub | C3 | -162 |
| HSP13-7 | May(2013） | *Ziziphus jujuba Mill. Var. spinosa* | Heshui | 36°01' | 108°06' | 1281 | Rhamnaceae | Dictyledoneae | shrub | C3 | -194 |
| HSP13-44 | May(3013） | *Artemisia* | Heshui | 36°01' | 108°06' | 1198 | | | shrub | | -150 |
| HSP13-3 | May(2013） | *Ameniaca sibirica* | Heshui | 36°01' | 108°06' | 1299 | Rosaceae | Dictyledoneae | tree | C3 | -183 |
| HSP13-51 | Sept.(2013) | *Robinia pseudoacia* | Heshui | 36°01' | 108°06' | 1245 | Leguminosae | Dictyledoneae | tree | C3 | -133 |
| HSP13-62 | Sept.(2013) | *Ameniaca sibirica* | Heshui | 36°01' | 108°06' | 1294 | Rosaceae | Dictyledoneae | tree | C3 | -197 |
| HSP13-69 | Sept.(2013) | *Ameniaca sibirica* | Heshui | 36°01' | 108°06' | 1255 | Rosaceae | Dictyledoneae | tree | C3 | -172 |

Table S2 Data sources, distribution, taxonomy, and references of plant leaf wax $\delta D_{n\text{-alkane}}$ values compiled from the Northern Hemisphere and used in this study.

| Sample date | Species | Location | Latitude | Longitude | Altitude/m | Family | Plant taxonomy | Plant life forms | photosynthetic pathway | $\delta D_{n\text{-alkane}}(C_{29}$,‰) | $\delta D_{p(model)}$ | $\varepsilon_{wax\text{-}p}$ | Ref. |
|---|---|---|---|---|---|---|---|---|---|---|---|---|---|
| Sept.(2012) | | Lantian | 34.3 | 109°9'E | 619 | Asteraceae | Dictyledoneae | shrub | | -158 | -50 | -114 | 1 |
| Sept.(2012) | *Lespedeza davurica* | Lantian | 34.3 | 109°10'E | 619 | Leguminosae | Dictyledoneae | shrub | C3 | -172 | -50 | -128 | 1 |
| Sept.(2012) | *Populus adenopoda* | Lantian | 34.3 | 109°11'E | 619 | Salicaceae | Dictyledoneae | tree | C3 | -154 | -50 | -109 | 1 |
| Sept.(2012) | *Populus adenopoda* | Lantian | 34.3 | 109°12'E | 619 | Salicaceae | Dictyledoneae | tree | C3 | -157 | -50 | -112 | 1 |
| Sept.(2012) | *Populus adenopoda* | Lantian | 34.4 | 109°13'E | 619 | Salicaceae | Dictyledoneae | tree | C3 | -161 | -50 | -117 | 1 |
| Sept.(2012) | *Populus adenopoda* | Lantian | 34.4 | 109°14'E | 619 | Salicaceae | Dictyledoneae | tree | C3 | -150 | -50 | -105 | 1 |
| Sept.(2012) | | Lantian | 34.4 | 109°15'E | 619 | | Dictyledoneae | tree | C3 | -156 | -50 | -111 | 1 |
| Sept.(2012) | | Lantian | 34.4 | 109°16'E | 619 | | Dictyledoneae | tree | C3 | -172 | -50 | -128 | 1 |
| Sept.(2012) | | Lantian | 34.4 | 109°17'E | 619 | | Dictyledoneae | tree | C3 | -176 | -50 | -132 | 1 |
| | *Cerasus yedoensis* | Xi'an | 34.4 | 108°56'E | 384 | Rosaceae | Dictyledoneae | tree | C3 | -173 | -47 | -132 | 1 |
| | *Buddleja alternifolia* | Xi'an | 34.4 | 108°56'E | 384 | Buddlejaceae | Dictyledoneae | shrub | C3 | -159 | -47 | -118 | 1 |
| | *Swida macrophylla* | Xi'an | 34.4 | 108°56'E | 384 | Cornaceae | Dictyledoneae | tree | | -169 | -47 | -128 | 1 |
| | *Cirsium setosum* | Xi'an | 34.4 | 108°56'E | 384 | Asteraceae | Dictyledoneae | grass | | -173 | -47 | -132 | 1 |
| | *Ligustrum compactum* | Xi'an | 34.4 | 108°56'E | 384 | Oleaceae | Dictyledoneae | tree | C3 | -164 | -47 | -122 | 1 |
| | *Potentilla kleiniana* | Xi'an | 34.4 | 108°56'E | 384 | Rosaceae | Dictyledoneae | grass | | -156 | -47 | -114 | 1 |
| May(2013） | *Lespedeza cuneata* | Heshui | 36 | 108°06'E | 1252 | Asteraceae | Dictyledoneae | grass | C3 | -166 | -59 | -114 | 2 |
| May(2013） | *Lespedeza cuneata* | Heshui | 36 | 108°06'E | 1246 | Asteraceae | Dictyledoneae | grass | C3 | -159 | -59 | -106 | 2 |
| May(2013） | *Ziziphus jujuba Mill. Var. spinosa* | Heshui | 36 | 108°06'E | 1246 | Rhamnaceae | Dictyledoneae | shrub | C3 | -161 | -59 | -108 | 2 |
| May(2013） | *Lespedeza cuneata* | Heshui | 36 | 108°06'E | 1230 | Asteraceae | Dictyledoneae | grass | C3 | -158 | -59 | -105 | 2 |
| May(2013） | *Ziziphus jujuba Mill. Var. spinosa* | Heshui | 36 | 108°06'E | 1230 | Rhamnaceae | Dictyledoneae | shrub | C3 | -186 | -59 | -135 | 2 |
| May(2013） | *Lespedeza cuneata* | Heshui | 36 | 108°06'E | 1299 | Asteraceae | Dictyledoneae | herb | C3 | -164 | -59 | -111 | 2 |
| May(2013） | *Lespedeza cuneata* | Heshui | 36.1 | 108°06'E | 1210 | Asteraceae | Dictyledoneae | herb | C3 | -152 | -59 | -99 | 2 |
| May(2013） | *Lespedeza cuneata* | Heshui | 36.1 | 108°06'E | 1203 | Asteraceae | Dictyledoneae | herb | C3 | -142 | -59 | -89 | 2 |
| May(2013） | *Ziziphus jujuba Mill. Var. spinosa* | Heshui | 36.1 | 108°06'E | 1203 | Rhamnaceae | Dictyledoneae | shrub | C3 | -152 | -59 | -99 | 2 |
| May(2013） | *Lespedeza cuneata* | Heshui | 36.1 | 108°06'E | 1196 | Asteraceae | Dictyledoneae | herb | C3 | -143 | -59 | -89 | 2 |
| May(2013） | *Ameniaca sibirica* | Heshui | 36.1 | 108°06'E | 1299 | Rosaceae | Dictyledoneae | tree | C3 | -183 | -59 | -132 | 2 |
| May(3013） | *Lespedeza cuneata* | Heshui | 36.1 | 108°06'E | 1257 | Asteraceae | Dictyledoneae | herb | C3 | -146 | -59 | -92 | 2 |
| May(3013） | *Lespedeza cuneata* | Heshui | 36.1 | 108°06'E | 1240 | Asteraceae | Dictyledoneae | herb | C3 | -143 | -59 | -89 | 2 |
| May(3013） | *Ziziphus jujuba Mill. Var. spinosa* | Heshui | 36.1 | 108°06'E | 1240 | Rhamnaceae | Dictyledoneae | shrub | C3 | -162 | -59 | -109 | 2 |
| May(3013） | *Lespedeza cuneata* | Heshui | 36.2 | 108°06'E | 1225 | Asteraceae | Dictyledoneae | grass | C3 | -148 | -59 | -94 | 2 |
| May(3013） | *Artemisia* | Heshui | 36.2 | 108°06'E | 1195 | | Dictyledoneae | shrub | | -161 | -59 | -108 | 2 |
| Sept.(2013) | *Ziziphus jujuba Mill. Var. spinosa* | Heshui | 36.2 | 108°06'E | 1256 | Rhamnaceae | Dictyledoneae | shrub | C3 | -182 | -59 | -131 | 2 |
| Sept.(2013) | *Robinia pseudoacia* | Heshui | 36.2 | 108°06'E | 1245 | Leguminosae | Dictyledoneae | tree | C3 | -133 | -59 | -79 | 2 |
| Sept.(2013) | *Lespedeza cuneata* | Heshui | 36.2 | 108°06'E | 1245 | Asteraceae | Dictyledoneae | grass | C3 | -176 | -59 | -125 | 2 |
| Sept.(2013) | *Ziziphus jujuba Mill. Var. spinosa* | Heshui | 36.2 | 108°06'E | 1234 | Rhamnaceae | Dictyledoneae | shrub | C3 | -176 | -59 | -124 | 2 |
| Sept.(2013) | *Artemisia capillaris* | Heshui | 36.2 | 108°06'E | 1234 | Asteraceae | Dictyledoneae | grass | c3 | -156 | -59 | -104 | 2 |
| Sept.(2013) | *Lespedeza cuneata* | Heshui | 36.2 | 108°06'E | 1234 | Asteraceae | Dictyledoneae | herb | C3 | -173 | -59 | -121 | 2 |
| Sept.(2013) | *Artemisia capillaris* | Heshui | 36.2 | 108°06'E | 1219 | Asteraceae | Dictyledoneae | herb | c3 | -154 | -59 | -101 | 2 |
| Sept.(2013) | *Artemisia giraldii* | Heshui | 36.2 | 108°06'E | 1219 | Asteraceae | Dictyledoneae | grass | C3 | -186 | -59 | -135 | 2 |
| Sept.(2013) | *Artemisia capillaris* | Heshui | 36.2 | 108°06'E | 1204 | Asteraceae | Dictyledoneae | grass | c3 | -153 | -59 | -100 | 2 |
| May(2013） | *Lespedeza cuneata* | Heshui | 36.2 | 108°06'E | 1281 | Asteraceae | Dictyledoneae | grass | C3 | -149 | -59 | -96 | 2 |
| Sept.(2013) | *Lespedeza bicolor* | Heshui | 36.2 | 108°06'E | 1204 | Leguminosae | Dictyledoneae | shrub | C3 | -162 | -59 | -109 | 2 |
| Sept.(2013) | *Ameniaca sibirica* | Heshui | 36.2 | 108°06'E | 1294 | Rosaceae | Dictyledoneae | tree | C3 | -197 | -59 | -147 | 2 |
| Sept.(2013) | *Lespedeza cuneata* | Heshui | 36.2 | 108°06'E | 1294 | Asteraceae | Dictyledoneae | grass | C3 | -185 | -59 | -134 | 2 |
| Sept.(2013) | *Lespedeza cuneata* | Heshui | 36.2 | 108°06'E | 1277 | Asteraceae | Dictyledoneae | herb | C3 | -175 | -59 | -123 | 2 |
| Sept.(2013) | *Lespedeza cuneata* | Heshui | 36.3 | 108°06'E | 1255 | Asteraceae | Dictyledoneae | grass | C3 | -169 | -59 | -117 | 2 |
| Sept.(2013) | *Ameniaca sibirica* | Heshui | 36.3 | 108°06'E | 1255 | Rosaceae | Dictyledoneae | tree | C3 | -172 | -59 | -120 | 2 |
| May(2013） | *Ziziphus jujuba Mill. Var. spinosa* | Heshui | 36.3 | 108°06'E | 1281 | Rhamnaceae | Dictyledoneae | shrub | C3 | -194 | -59 | -143 | 2 |
| Sept.(2013) | *Lespedeza cuneata* | Heshui | 36.3 | 108°06'E | 1228 | Asteraceae | Dictyledoneae | herb | C3 | -181 | -59 | -130 | 2 |
| Sept.(2013) | *Lespedeza cuneata* | Heshui | 36.3 | 108°06'E | 1213 | Asteraceae | Dictyledoneae | herb | C3 | -166 | -59 | -114 | 2 |
| Sept.(2013) | *Artemisia capillaris* | Heshui | 36.3 | 108°06'E | 1213 | Asteraceae | Dictyledoneae | herb | c3 | -165 | -59 | -112 | 2 |
| May(2013） | *Lespedeza cuneata* | Heshui | 36.3 | 108°06'E | 1266 | Asteraceae | Dictyledoneae | herb | C3 | -143 | -59 | -89 | 2 |
| | *Corispermum hyssopifolium* | Ertuokeqi | 39 | 107°58'E | 1389 | Chenopodiaceae | Dictyledoneae | shrub | C4 | -116 | -62 | -58 | 3 |
| | *Oxytropis aciphylla* | Ertuokeqi | 39 | 107°58'E | 1389 | Labiatae | Dictyledoneae | shrub | C3 | -162 | -62 | -107 | 3 |
| | *Agropyron desertorum* | Ertuokeqi | 39 | 107°58'E | 1389 | Poaceae | Monocotyledonous | grass | C3 | -185 | -62 | -131 | 3 |
| | *Caragana stenophylla* | Ertuokeqi | 39 | 107°58'E | 1389 | Leguminosae | Dictyledoneae | shrub | C3 | -133 | -62 | -76 | 3 |
| | *Haloxylon ammodendron* | Jitantai | 39.8 | 105°45'E | 1027 | Chenopodiaceae | Dictyledoneae | shrub | C4 | -140 | -55 | -90 | 3 |
| | *Haloxylon ammodendron* | Jitantai | 39.8 | 105°45'E | 1027 | Chenopodiaceae | Dictyledoneae | shrub | C4 | -114 | -55 | -62 | 3 |
| | *Heteropappus less* | Lantian | 34.2 | 109°7'E | 619 | Asteraceae | Dictyledoneae | herb | C3 | -186 | -50 | -143 | 3 |

| | | | | | | | | | | | | | |
|---|---|---|---|---|---|---|---|---|---|---|---|---|---|
| | *Salsola collina* | Lanzhou | 36 | 103°50′E | 1627 | Chenopodiaceae | Dictyledoneae | shrub | C4 | -130 | -56 | -78 | 3 |
| | *Prinsepia* | Yanan | 36.7 | 109°29′E | 1061 | Rosaceae | Dictyledoneae | shrub | C4 | -156 | -58 | -104 | 3 |
| | *Vitex negundo* | Yanan | 36.7 | 109°29′E | 1061 | Verbenaceae | Dictyledoneae | shrub | C3 | -145 | -58 | -92 | 3 |
| | *Drancocephalum moldavica* | Yanan | 36.7 | 109°29′E | 1061 | Labiatae | Dictyledoneae | shrub | C3 | -167 | -58 | -116 | 3 |
| | *Artemisia scoparia* | Yanan | 36.7 | 109°29′E | 1061 | Chenopodiaceae | Dictyledoneae | shrub | C3 | -142 | -58 | -89 | 3 |
| | *Peganum harmala* | Yanchi | 37.5 | 107°24′E | 1351 | Oxalidaceae | Dictyledoneae | shrub | C3 | -160 | -59 | -107 | 3 |
| | *Lespedeza davurica* | Yijun | 38.5 | 106°14′E | 1114 | Leguminosae | Dictyledoneae | shrub | C3 | -136 | -54 | -87 | 3 |
| | *Oxytropis aciphylla* | Yingchuan | 38.5 | 106°13′E | 1111 | Labiatae | Dictyledoneae | shrub | C3 | -158 | -54 | -110 | 3 |
| | *Heteropappus less* | Yuxian | 37 | 113°02′E | 1202 | Asteraceae | Dictyledoneae | herb | C3 | -197 | -65 | -141 | 4 |
| | *Artemisia gmelinii* | Yuxian | 37 | 113°02′E | 1202 | Asteraceae | Dictyledoneae | herb | C3 | -166 | -65 | -108 | 4 |
| | *Artemisia giraldii* | Yuxian | 37 | 113°02′E | 1202 | Asteraceae | Dictyledoneae | herb | C3 | -182 | -65 | -125 | 4 |
| | *Potentilla discolor* | Luochuan | 35.85 | 109°30′E | 1132 | Rosaceae | Dictyledoneae | herb | C3 | -175 | -59 | -123 | 4 |
| | *Heteropappus less* | Luochuan | 35.85 | 109°30′E | 1132 | Asteraceae | Dictyledoneae | herb | C3 | -189 | -59 | -138 | 4 |
| | *Artemisia giraldii* | Luochuan | 35.85 | 109°30′E | 1132 | Asteraceae | Dictyledoneae | herb | C3 | -173 | -59 | -121 | 4 |
| | *Wikstroemia chamaedaphne* | Ruicheng | 34.8 | 110°25′E | 796 | Thymelaeaceae | Dictyledoneae | shrub | C3 | -184 | -54 | -137 | 4 |
| | *Spathodea campanulata* | Sanya | 18.2 | 109°30′E | 8 | Bignoniaceae | Dictyledoneae | tree | C3 | -140 | -36 | -108 | 4 |
| | *Artocarpus heterophyllus Lam.* | Sanya | 18.2 | 109°30′E | 8 | Moraceae | Dictyledoneae | tree | C3 | -119 | -36 | -86 | 4 |
| | *Michelia alba* | Sanya | 18.2 | 109°30′E | 8 | Magnoliaceae | Dictyledoneae | tree | C3 | -186 | -36 | | 4 |
| | *Tamarindus indica Linn.* | Sanya | 18.2 | 109°30′E | 8 | Caesalpiniaceae | Dictyledoneae | tree | C3 | -154 | -36 | -122 | 4 |
| | *Lespedeza davurica* | Yuxian | 37 | 113°02′E | 1202 | Leguminosae | Dictyledoneae | shrub | C3 | -153 | -65 | -94 | 4 |
| | *Var. spinosa(Bunge)* | Yuxian | 37 | 113°02′E | 1202 | Rhamnaceae | Dictyledoneae | Shrub | C3 | -156 | -65 | -97 | 4 |
| | *Lespedeza davurica* | Luochuan | 35.8 | 109°30′E | 1132 | Leguminosae | Dictyledoneae | shrub | C3 | -141 | -59 | -87 | 4 |
| | *Var. spinosa(Bunge)* | Ruicheng | 34.7 | 110°25′E | 796 | Rhamnaceae | Dictyledoneae | shrub | C3 | -145 | -54 | -96 | 4 |
| August(2005) | *Betula populifolia Marsh(6m)* | Blood pond | 42 | 71°58′W | 212 | Betulaceae | Dictyledoneae | tree | C3 | -182 | -62 | -128 | 6 |
| August(2005) | *Betula populifolia Marsh(4.5m)* | Blood pond | 42 | 71°58′W | 212 | Betulaceae | Dictyledoneae | tree | C3 | -176 | -62 | -122 | 6 |
| August(2005) | *Betula populifolia Marsh(3m)* | Blood pond | 42 | 71°58′W | 212 | Betulaceae | Dictyledoneae | tree | C3 | -190 | -62 | -136 | 6 |
| August(2005) | *Betula populifolia Marsh(6m)* | Blood pond | 42 | 71°58′W | 212 | Betulaceae | Dictyledoneae | tree | C3 | -178 | -62 | -124 | 6 |
| August(2005) | *Betula populifolia Marsh(4.5m)* | Blood pond | 42 | 71°58′W | 212 | Betulaceae | Dictyledoneae | tree | C3 | -177 | -62 | -123 | 6 |
| August(2005) | *Quercus velutina Lam.* | Blood pond | 42 | 71°58′W | 212 | Fagaceae | Dictyledoneae | tree | C3 | -167 | -62 | -112 | 6 |
| August(2005) | *Acer rubrum L.* | Blood pond | 42 | 71°58′W | 212 | Aceraceae | Dictyledoneae | tree | C3 | -194 | -62 | -141 | 6 |
| August(2005) | *Acer rubrum L.* | Blood pond | 42 | 71°58′W | 212 | Aceraceae | Dictyledoneae | tree | C3 | -195 | -62 | -142 | 6 |
| August(2005) | *Acer rubrum L.(6m)* | Blood pond | 42.1 | 71°58′W | 212 | Aceraceae | Dictyledoneae | tree | C3 | -206 | -62 | | 6 |
| August(2005) | *Acer rubrum L.(4.5m)* | Blood pond | 42.1 | 71°58′W | 212 | Aceraceae | Dictyledoneae | tree | C3 | -219 | -62 | | 6 |
| August(2005) | *Betula lenta L.(6m)* | Blood pond | 42.1 | 71°58′W | 212 | Betulaceae | Dictyledoneae | tree | C3 | -182 | -62 | -128 | 6 |
| August(2005) | *Betula lenta L.(4.5m)* | Blood pond | 42.1 | 71°58′W | 212 | Betulaceae | Dictyledoneae | tree | C3 | -197 | -62 | -144 | 6 |
| August(2005) | *Betula lenta L.(3m)* | Blood pond | 42.1 | 71°58′W | 212 | Betulaceae | Dictyledoneae | tree | C3 | -181 | -62 | -127 | 6 |
| August(2005) | *Betula lenta L.(6m)* | Blood pond | 42.1 | 71°58′W | 212 | Betulaceae | Dictyledoneae | tree | C3 | -168 | -62 | -113 | 6 |
| August(2005) | *Betula lenta L.(4.5m)* | Blood pond | 42.2 | 71°58′W | 212 | Betulaceae | Dictyledoneae | tree | C3 | -174 | -62 | -119 | 6 |
| August(2005) | *Betula lenta L.* | Blood pond | 42.2 | 71°58′W | 212 | Betulaceae | Dictyledoneae | tree | C3 | -174 | -62 | -119 | 6 |
| August(2005) | *Carya sp. Nutt.(6m)* | Blood pond | 42.2 | 71°58′W | 212 | Juglandaceae | Dictyledoneae | tree | C3 | -194 | -62 | -141 | 6 |
| August(2005) | *Carya sp. Nutt.(4.5m)* | Blood pond | 42.2 | 71°58′W | 212 | Juglandaceae | Dictyledoneae | tree | C3 | -188 | -62 | -134 | 6 |
| August(2005) | *Carya sp. Nutt.(3m)* | Blood pond | 42.2 | 71°58′W | 212 | Juglandaceae | Dictyledoneae | tree | C3 | -176 | -62 | -122 | 6 |
| August(2005) | *Carya sp. Nutt.* | Blood pond | 42.2 | 71°58′W | 212 | Juglandaceae | Dictyledoneae | tree | C3 | -208 | -62 | -156 | 6 |
| August(2005) | *Prunus serotina Ehrh.(6m)* | Blood pond | 42.2 | 71°58′W | 212 | Rosaceae | Dictyledoneae | tree | C3 | -184 | -62 | -130 | 6 |
| August(2005) | *Prunus serotina Ehrh.(4.5m)* | Blood pond | 42.2 | 71°58′W | 212 | Rosaceae | Dictyledoneae | tree | C3 | -173 | -62 | -118 | 6 |
| August(2005) | *Quercus rubra L.(6m)* | Blood pond | 42.2 | 71°58′W | 212 | Fagaceae | Dictyledoneae | tree | C3 | -165 | -62 | -110 | 6 |
| August(2005) | *Quercus rubra L.(3m)* | Blood pond | 42.4 | 71°58′W | 212 | Fagaceae | Dictyledoneae | tree | C3 | -173 | -62 | -118 | 6 |
| August(2005) | *Fraxinus americana L.(6m)* | Blood pond | 42.4 | 71°58′W | 212 | Oleaceae | Dictyledoneae | tree | C3 | -185 | -62 | -131 | 6 |
| August(2005) | *Fraxinus americana L.(4.5m)* | Blood pond | 42.4 | 71°58′W | 212 | Oleaceae | Dictyledoneae | tree | C3 | -182 | -62 | -128 | 6 |
| August(2005) | *Fraxinus americana L.(3m)* | Blood pond | 42.4 | 71°58′W | 212 | Oleaceae | Dictyledoneae | tree | C3 | -187 | -62 | -133 | 6 |
| August(2005) | *Nyssa sylvatica Marsh.(6m)* | Blood pond | 42.4 | 71°58′W | 212 | Cornaceae | Dictyledoneae | tree | C3 | -180 | -62 | -126 | 6 |
| August(2005) | *Nyssa sylvatica Marsh.(4.5m)* | Blood pond | 42.4 | 71°58′W | 212 | Cornaceae | Dictyledoneae | tree | C3 | -187 | -62 | -133 | 6 |
| August(2005) | *Nyssa sylvatica Marsh.(3m)* | Blood pond | 42.4 | 71°58′W | 212 | Cornaceae | Dictyledoneae | tree | C3 | -188 | -62 | -134 | 6 |
| August(2005) | *Hamamelis virginiana L.* | Blood pond | 42.4 | 71°58′W | 212 | Hamamelidaceae | Dictyledoneae | shrub | C3 | -188 | -62 | -134 | 6 |
| August(2005) | *Hamamelis virginiana L.* | Blood pond | 42.4 | 71°58′W | 212 | Hamamelidaceae | Dictyledoneae | shrub | C3 | -170 | -62 | -115 | 6 |
| August(2005) | *Hamamelis virginiana L.* | Blood pond | 42.4 | 71°58′W | 212 | Hamamelidaceae | Dictyledoneae | shrub | C3 | -168 | -62 | -113 | 6 |
| August(2005) | *Lonicera tatarica L.* | Blood pond | 42.4 | 71°58′W | 212 | Carifoliaceae | Dictyledoneae | shrub | C3 | -185 | -62 | -131 | 6 |
| August(2005) | *Rubus allegheniensis* | Blood pond | 42.4 | 71°58′W | 212 | Rosaceae | Dictyledoneae | shrub | C3 | -187 | -62 | -133 | 6 |
| August(2005) | *Viburnum acerifolium L.* | Blood pond | 42.25 | 71°58′W | 212 | Adoxaceae | Dictyledoneae | shrub | C3 | -187 | -62 | -133 | 6 |
| August(2005) | *Clethra alnifolia L.* | Blood pond | 42.25 | 71°58′W | 212 | Clethraceae | Dictyledoneae | shrub | C3 | -197 | -62 | -144 | 6 |
| August(2005) | *Rhododendron sp. L.* | Blood pond | 42.25 | 71°58′W | 212 | Ericaceae | Dictyledoneae | shrub | C3 | -190 | -62 | -136 | 6 |
| August(2005) | *Vitis aestivalis Michx.* | Blood pond | 42.25 | 71°58′W | 212 | Vitaceae | Dictyledoneae | vine | C3 | -193 | -62 | -140 | 6 |
| August(2005) | *Aster Divaricatus L.* | Blood pond | 42.25 | 71°58′W | 212 | Asteraceae | Dictyledoneae | herb | C3 | -176 | -62 | -122 | 6 |
| August(2005) | *Daucus carota L.* | Blood pond | 42.25 | 71°58′W | 212 | Apiaceae | Dictyledoneae | herb | C3 | -170 | -62 | -115 | 6 |
| August(2005) | *Plantago major L.* | Blood pond | 42.25 | 71°58′W | 212 | Plantaginaceae | Dictyledoneae | herb | C3 | -204 | -62 | -151 | 6 |

| | | | | | | | | | | | | | |
|---|---|---|---|---|---|---|---|---|---|---|---|---|---|
| August(2005) | *Trifolium pratense L.* | Blood pond | 42.25 | 71°58'W | 212 | Leguminosae | Dictyledoneae | herb | C3 | -207 | -62 | -155 | 6 |
| August(2005) | *Impatiens capensis* | Blood pond | 42.25 | 71°58'W | 212 | Balsaminaceae | Dictyledoneae | herb | C3 | -187 | -62 | -133 | 6 |
| August(2005) | *Mimulus ringens L.* | Blood pond | 42.25 | 71°58'W | 212 | Phrymaceae | Dictyledoneae | herb | C3 | -173 | -62 | -118 | 6 |
| Sept.(2006) | *Arctostaphylos pringlei* | Jacinto | 33.8 | 116°46'W | 1620 | Ericaceae | Dictyledoneae | shrub | C3 | -145 | -83 | -68 | 7 |
| Sept.(2006) | *Arctostaphylos pringlei* | Jacinto | 33.8 | 116°46'W | 1620 | Ericaceae | Dictyledoneae | shrub | C3 | -191 | -83 | -118 | 7 |
| Sept.(2006) | *Quercus chrysolepsis* | Jacinto | 33.8 | 116°46'W | 1620 | Fagaceae | Dictyledoneae | tree | C3 | -159 | -83 | -83 | 7 |
| Sept.(2006) | *Quercus chrysolepsis* | Jacinto | 33.8 | 116°46'W | 1620 | Fagaceae | Dictyledoneae | tree | C3 | -131 | -83 | -52 | 7 |
| Sept.(2006) | *Quercus chrysolepsis* | Jacinto | 33.8 | 116°46'W | 1620 | Fagaceae | Dictyledoneae | tree | C3 | -143 | -83 | -65 | 7 |
| Sept.(2006) | *Quercus kelloggi* | Jacinto | 33.9 | 116°46'W | 1620 | Fagaceae | Dictyledoneae | tree | C3 | -160 | -83 | -84 | 7 |
| Sept.(2006) | *Quercus kelloggi* | Jacinto | 33.9 | 116°46'W | 1620 | Fagaceae | Dictyledoneae | tree | C3 | -147 | -83 | -70 | 7 |
| Sept.(2006) | *Quercus kelloggi* | Jacinto | 33.9 | 116°46'W | 1620 | Fagaceae | Dictyledoneae | tree | C3 | -137 | -83 | -59 | 7 |
| Sept.(2006) | *Erigonium wrightii* | Jacinto | 33.9 | 116°46'W | 1620 | | Dictyledoneae | | C3 | -119 | -83 | -39 | 7 |
| Sept.(2006) | *Salix lasiolepsis* | Jacinto | 33.9 | 116°46'W | 1620 | | Dictyledoneae | | C3 | -145 | -83 | -68 | 7 |
| Feb.(2007) | *Coleogyne ramosissima* | Mojave | 34.8 | 115°40'W | 1500 | Rosaceae | Dictyledoneae | shrub | C3 | -192 | -83 | -119 | 7 |
| Feb.(2007) | *Epilobium canum sp. Latifolium* | Mojave | 34.8 | 115°40'W | 1500 | Onagraceae | Dictyledoneae | shrub | C3 | -207 | -83 | -135 | 7 |
| Feb.(2007) | *Ericameria cuneata var. spathulata* | Mojave | 34.8 | 115°40'W | 1500 | Asteraceae | Dictyledoneae | shrub | C3 | -143 | -83 | -65 | 7 |
| Feb.(2007) | *Larrea tridentata* | Mojave | 34.8 | 115°40'W | 1500 | Zygophyllaceae | Dictyledoneae | shrub | C3 | -144 | -83 | -67 | 7 |
| Feb.(2007) | *Rhamnus ilicifolia* | Mojave | 34.8 | 115°40'W | 1500 | Rhamnaceae | Dictyledoneae | shrub | C3 | -156 | -83 | -80 | 7 |
| Feb.(2007) | *Salix exigua* | Mojave | 34.8 | 115°40'W | 1500 | Salicaceae | Dictyledoneae | shrub | C3 | -197 | -83 | -124 | 7 |
| Feb.(2007) | *Quercus chrysolepsis* | Mojave | 34.8 | 115°40'W | 1500 | Fagaceae | Dictyledoneae | tree | C3 | -151 | -83 | -74 | 7 |
| Feb.(2007) | *Artemisia ludoviciana sp. Albula* | Mojave | 34.8 | 115°40'W | 1500 | Asteraceae | Dictyledoneae | | C3 | -127 | -83 | -48 | 7 |
| April.(2007) | *Ceanothus leucodermis* | Pasadena | 34.15 | 118°9'W | 640 | Rhamnaceae | Dictyledoneae | shrub | C3 | -156 | -70 | -92 | 7 |
| April.(2007) | *Quercus agrifolia* | Pasadena | 34.15 | 118°9'W | 640 | Fagaceae | Dictyledoneae | tree | C3 | -139 | -70 | -74 | 7 |
| April.(2007) | *Quercus agrifolia* | Pasadena | 34.15 | 118°9'W | 640 | Fagaceae | Dictyledoneae | tree | C3 | -127 | -70 | -61 | 7 |
| April.(2007) | *Quercus agrifolia* | Pasadena | 34.15 | 118°9'W | 640 | Fagaceae | Dictyledoneae | tree | C3 | -136 | -70 | -71 | 7 |
| Nov.(2007) | *Adenostoma fasciculatum* | Topanga | 34.05 | 118°35'W | 400 | Rosaceae | Dictyledoneae | shrub | C3 | -121 | -66 | -59 | 7 |
| Nov.(2007) | *Artemisia californica* | Topanga | 34.05 | 118°35'W | 400 | Asteraceae | Dictyledoneae | shrub | C3 | -125 | -66 | -63 | 7 |
| Nov.(2007) | *Ceanothus intergerrimus* | Topanga | 34.05 | 118°35'W | 400 | Rhamnaceae | Dictyledoneae | shrub | C3 | -126 | -66 | -64 | 7 |
| Nov.(2007) | *Ceanothus megacarpus* | Topanga | 34.05 | 118°35'W | 400 | Rhamnaceae | Dictyledoneae | shrub | C3 | -136 | -66 | -75 | 7 |
| Nov.(2007) | *Ceanothus megacarpus* | Topanga | 34.05 | 118°35'W | 400 | Rhamnaceae | Dictyledoneae | shrub | C3 | -130 | -66 | -69 | 7 |
| Nov.(2007) | *Ceanothus spinosus* | Topanga | 34.05 | 118°35'W | 400 | Rhamnaceae | Dictyledoneae | shrub | C3 | -128 | -66 | -66 | 7 |
| Nov.(2007) | *Heteromeies arbutifolia* | Topanga | 34.05 | 118°35'W | 400 | Rosaceae | Dictyledoneae | shrub | C3 | -132 | -66 | -71 | 7 |
| Nov.(2007) | *Lonicera subspicata* | Topanga | 34.05 | 118°35'W | 400 | Caprifoloaceae | Dictyledoneae | shrub | C3 | -132 | -66 | -71 | 7 |
| Nov.(2007) | *Phacelia cicutaria* | Topanga | 34.05 | 118°35'W | 400 | Boraginaceae | Dictyledoneae | shrub | C3 | -118 | -66 | -56 | 7 |
| Nov.(2007) | *Quercus dumosa* | Topanga | 34 | 118°35'W | 400 | Fagaceae | Dictyledoneae | shrub | C3 | -119 | -66 | -57 | 7 |
| Nov.(2007) | *Sambucus mexicana* | Topanga | 34 | 118°35'W | 400 | Adoxaceae | Dictyledoneae | shrub | C3 | -133 | -66 | -72 | 7 |
| Nov.(2007) | *Quercus agrifolia* | Topanga | 34 | 118°35'W | 400 | Fagaceae | Dictyledoneae | tree | C3 | -126 | -66 | -64 | 7 |
| Nov.(2007) | *Quercus agrifolia* | Topanga | 34 | 118°35'W | 400 | Fagaceae | Dictyledoneae | tree | C3 | -120 | -66 | -58 | 7 |
| Nov.(2007) | *Keckieiia cordifolia* | Topanga | 34 | 118°35'W | 400 | | Dictyledoneae | | C3 | -130 | -66 | -69 | 7 |
| Nov.(2007) | *Maiacothamnus fasciculatus* | Topanga | 34 | 118°35'W | 400 | | Dictyledoneae | | C3 | -102 | -66 | -39 | 7 |
| | *Salix arctica Pall.* | Axel Heiberg Island | 79.9 | 89°01'W | 357 | Salicaceae | Dictyledoneae | shrub | C3 | -251 | -197 | -67 | 8 |
| | *Rhododendron groenlandicum(Oeder)* | Denali | 63.7 | 148°54'W | 551 | Ericaceae | Dictyledoneae | shrub | C3 | -248 | -149 | -116 | 8 |
| | *Solidago vigaurea L.* | Helsinki | 60.2 | 24°56'E | 16 | Asteraceae | Dictyledoneae | herb | C3 | -188 | -86 | -112 | 8 |
| | *Antennaria dioica(L.) Gaertn.* | Helsinki | 60.2 | 24°56'E | 16 | Asteraceae | Dictyledoneae | herb | C3 | -189 | -86 | -113 | 8 |
| | *Matricaria matricarioides(Less.)Porter* | Helsinki | 60.2 | 24°56'E | 16 | Asteraceae | Dictyledoneae | herb | C3 | -156 | -86 | -77 | 8 |
| | *Lonicera tatarica L.* | Helsinki | 60.2 | 24°56'E | 16 | Caprifoliaceae | Dictyledoneae | shrub | C3 | -161 | -86 | -82 | 8 |
| | *Cotinus coggyria Scop.* | Stockholm | 59.3 | 18°02'E | 39 | Anacardiaceae | Dictyledoneae | shrub | C3 | -182 | -81 | -110 | 8 |
| | *Castanea sativa Mill.* | Stockholm | 59.3 | 18°02'E | 39 | Fagaceae | Dictyledoneae | tree | C3 | -151 | -81 | -76 | 8 |
| | *Thamnocalamus spathaceus* | Stockholm | 59.3 | 18°02'E | 39 | Poaceae | Dictyledoneae | shrub | C3 | -162 | -81 | -88 | 8 |
| | *Achillea millefolium L.* | Yarrow | 61.7 | 148°56'W | 340 | Asteraceae | Dictyledoneae | herb | C3 | -190 | -139 | -59 | 8 |
| May(1999) | *Benthamidia japonica* | Gunma-Japan | 36.35 | 139°37'E | 114 | Cornaceae | Dictyledoneae | tree | C3 | -158 | -50 | -114 | 9 |
| May(1999) | *Acer carpinifolium* | Gunma-Japan | 36.35 | 139°37'E | 114 | Sapindaceae | Dictyledoneae | tree | C3 | -134 | -50 | -88 | 9 |
| May(1999) | *Acer argutum* | Gunma-Japan | 36.35 | 139°37'E | 114 | Aceraceae | Dictyledoneae | tree | C3 | -119 | -50 | -73 | 9 |
| Oct.(1999) | *Benthamidia japonica* | Gunma-Japan | 36.35 | 139°37'E | 114 | Cornaceae | Dictyledoneae | tree | C3 | -170 | -50 | -126 | 9 |
| Oct.(1999) | *Prunus jamasakura* | Gunma-Japan | 36.35 | 139°37'E | 114 | Rosaceae | Dictyledoneae | shrub | C3 | -183 | -50 | -140 | 9 |
| Oct.(1999) | *Acer carpinifolium* | Gunma-Japan | 36.35 | 139°37'E | 114 | Sapindaceae | Dictyledoneae | tree | C3 | -161 | -50 | -117 | 9 |
| Oct.(1999) | *Acer argutum* | Gunma-Japan | 36.35 | 139°37'E | 114 | Aceraceae | Dictyledoneae | tree | C3 | -123 | -50 | -77 | 9 |
| May(2000) | *Taraxacum officinale* | Gunma-Japan | 36.35 | 139°37'E | 114 | Asteraceae | Dictyledoneae | herb | C3 | -155 | -50 | -111 | 9 |
| May(2000) | *Plantago asiatica* | Gunma-Japan | 36.35 | 139°37'E | 114 | Plantaginaceae | Dictyledoneae | herb | C3 | -178 | -50 | -135 | 9 |
| Oct.(2000) | *Artemisia princeps* | Gunma-Japan | 36.35 | 139°37'E | 114 | Asteraceae | Dictyledoneae | herb | C3 | -157 | -50 | -113 | 9 |
| Oct.(2000) | *Acer palmatum* | Gunma-Japan | 36.35 | 139°37'E | 114 | Sapindaceae | Dictyledoneae | tree | C3 | -171 | -50 | -127 | 9 |
| Oct.(2000) | *Quercus mongolica* | Gunma-Japan | 36.35 | 139°37'E | 114 | Fagaceae | Dictyledoneae | shrub | C3 | -188 | -50 | -145 | 9 |
| Oct.(2000) | *Quercus dentata* | Gunma-Japan | 36.35 | 139°37'E | 114 | Fagaceae | Dictyledoneae | tree | C3 | -193 | -50 | -151 | 9 |
| Sept.(2000) | *Albizia julibrissin* | Ogasawara-Japan | 27 | 142°11'E | 47 | Fabaceae | Dictyledoneae | tree | C3 | -124 | -36 | -91 | 9 |
| Oct.(1997) | *Manihot utilissima* | Thailand | 15.8 | 100°59'E | 114 | Euphorbiaceae | Dictyledoneae | shrub | C3 | -134 | -40 | -98 | 9 |

| | | | | | | | | | | | | | |
|---|---|---|---|---|---|---|---|---|---|---|---|---|---|
| July(1998) | *Quercus acutissima* | Tokyo-Japan | 35.75 | 139°30′E | 37 | Fagaceae | Dictyledoneae | tree | C3 | -145 | -48 | -102 | 9 |
| Oct.(1998) | *Camellia sasanqua* | Tokyo-Japan | 35.75 | 139°30′E | 37 | Theaceae | Dictyledoneae | tree | C3 | -167 | -48 | -125 | 9 |
| Aug-Sept(2002) | *Alnus incana* | Lago di Massachiucoli | 43.8 | 18°49′E | 18 | Betulaceae | Dictyledoneae | tree | C3 | -174 | -42 | -138 | 10 |
| Aug-Sept(2002) | *Betula pubescens* | Tunturilampi(FIN) | 67.4 | 27°10′E | 293 | Betulaceae | Dictyledoneae | tree | C3 | -185 | -99 | -95 | 10 |
| Aug-Sept(2002) | *Betula pubescens* | Near village of Luosto(FIN) | 67.2 | 26°51′E | 300 | Betulaceae | Dictyledoneae | tree | C3 | -167 | -99 | -75 | 10 |
| Aug-Sept(2002) | *Betula pubescens* | Kiuvajarvi(FIN) | 61.9 | 24°16′E | 149 | Betulaceae | Dictyledoneae | tree | C3 | -189 | -91 | -108 | 10 |
| Aug-Sept(2002) | *Betula pubescens* | Paajarvi(FIN) | 61.55 | 25°5′E | 151 | Betulaceae | Dictyledoneae | tree | C3 | -160 | -91 | -76 | 10 |
| Aug-Sept(2002) | *Betula pubescens* | Holzmaar(GER) | 50 | 6°52′E | 436 | Betulaceae | Dictyledoneae | tree | C3 | -169 | -60 | -116 | 10 |
| Aug-Sept(2002) | *Carpinusbetulus* | Lago di MEZZANO | 42.5 | 11°46′E | 466 | Betulaceae | Dictyledoneae | tree | C3 | -153 | -43 | -115 | 10 |
| Aug-Sept(2002) | *Fagus sylvatica* | Lago Grande di Monticchio(ITA) | 40.8 | 15°36′E | 674 | Fagaceae | Dictyledoneae | tree | C3 | -149 | -43 | -111 | 10 |
| Aug-Sept(2002) | *Myrtus* | Syrjanalunen(FIN) | 61.1 | 25°8′E | 156 | Mytaceae | Dictyledoneae | shrub | C3 | -231 | -91 | -154 | 10 |
| Aug-Sept(2002) | *Petraea* | Lago di MEZZANO | 42.6 | 11°46′E | 466 | Fagaceae | Dictyledoneae | tree | C3 | -161 | -42 | -124 | 10 |
| Aug-Sept(2002) | *Quercus* | Lago di MEZZANO | 42.6 | 11°46′E | 466 | Fagaceae | Dictyledoneae | tree | C3 | -157 | -42 | -120 | 10 |
| Aug-Sept(2002) | *Quercus cerris* | Lago di Massachiucoli | 43.8 | 18°49′E | 18 | Fagaceae | Dictyledoneae | tree | C3 | -174 | -43 | -137 | 10 |
| Aug-Sept(2002) | *Quercus robur* | Near village og Castigliano(ITA) | 42.6 | 10°55′E | 11 | Fagaceae | Dictyledoneae | tree | C3 | -174 | -37 | -142 | 10 |
| Aug-Sept(2002) | *Quercus variabilis* | Lago di Massachiucoli | 43.8 | 18°49′E | 18 | Fagaceae | Dictyledoneae | tree | C3 | -181 | -43 | -144 | 10 |
| May(2004) | *Acer pseudoplatanus* | Eisenach | 51 | 10°27′E | 440 | Sapindaceae | Dictyledoneae | tree | C3 | -169 | -64 | -112 | 11 |
| May(2004) | *Acer pseudoplatanus* | Eisenach | 51 | 10°27′E | 440 | Sapindaceae | Dictyledoneae | tree | C3 | -159 | -64 | -101 | 11 |
| June(2004) | *Acer pseudoplatanus* | Eisenach | 51 | 10°27′E | 440 | Sapindaceae | Dictyledoneae | tree | C3 | -181 | -64 | -125 | 11 |
| July(2004) | *Acer pseudoplatanus* | Eisenach | 51 | 10°27′E | 440 | Sapindaceae | Dictyledoneae | tree | C3 | -183 | -64 | -127 | 11 |
| August(2004) | *Acer pseudoplatanus* | Eisenach | 51 | 10°27′E | 440 | Sapindaceae | Dictyledoneae | tree | C3 | -175 | -64 | -119 | 11 |
| Sept.(2004) | *Acer pseudoplatanus* | Eisenach | 51 | 10°27′E | 440 | Sapindaceae | Dictyledoneae | tree | C3 | -169 | -64 | -112 | 11 |
| Oct.(2004) | *Acer pseudoplatanus* | Eisenach | 51 | 10°27′E | 440 | Sapindaceae | Dictyledoneae | tree | C3 | -187 | -64 | -131 | 11 |
| Oct.(2004) | *Acer pseudoplatanus* | Eisenach | 51 | 10°27′E | 440 | Sapindaceae | Dictyledoneae | tree | C3 | -176 | -64 | -120 | 11 |
| Nov.(2004) | *Acer pseudoplatanus* | Eisenach | 51 | 10°27′E | 440 | Sapindaceae | Dictyledoneae | tree | C3 | -192 | -64 | -137 | 11 |
| Nov.(2004) | *Acer pseudoplatanus* | Eisenach | 51 | 10°27′E | 440 | Sapindaceae | Dictyledoneae | tree | C3 | -190 | -64 | -135 | 11 |
| June(2011) | *Salicornia Europaea* | Stiffkey | 52.98 | 0°55′E | 1 | Amaranthaceae | Dictyledoneae | succulent | C3 | -132 | -58 | -79 | 13 |
| June(2011) | *Limonium vulgare* | Stiffkey | 52.98 | 0°55′E | 1 | Plumbaginaceae | Dictyledoneae | herb | C3 | -113 | -58 | -58 | 13 |
| June(2011) | *Atriplex portulacoides* | Stiffkey | 52.98 | 0°55′E | 1 | Amaranthaceae | Dictyledoneae | shrub | c3 | -127 | -58 | -73 | 13 |
| June(2011) | *Suaeda vera* | Stiffkey | 52.98 | 0°55′E | 1 | Amaranthaceae | Dictyledoneae | succulent | C3 | -119 | -58 | -65 | 13 |
| June(2011) | *Atriplex portulacoides* | Stiffkey | 52.98 | 0°55′E | 1 | Amaranthaceae | Dictyledoneae | shrub | C3 | -140 | -58 | -87 | 13 |
| June(2011) | *Suaeda vera* | Stiffkey | 52.98 | 0°55′E | 1 | Amaranthaceae | Dictyledoneae | succulent | C3 | -116 | -58 | -62 | 13 |
| June(2011) | *Limonium vulgare* | Stiffkey | 52.98 | 0°55′E | 1 | Plumbaginaceae | Dictyledoneae | herb | C3 | -114 | -58 | -59 | 13 |
| June(2011) | *Atriplex portulacoides* | Stiffkey | 52.98 | 0°55′E | 1 | Amaranthaceae | Dictyledoneae | shrub | C3 | -132 | -58 | -79 | 13 |
| March(2012) | *Atriplex portulacoides* | Stiffkey | 52.98 | 0°55′E | 1 | Amaranthaceae | Dictyledoneae | succulent | C3 | -139 | -58 | -86 | 13 |
| March(2012) | *Limonium vulgare* | Stiffkey | 52.98 | 0°55′E | 1 | Plumbaginaceae | Dictyledoneae | herb | C3 | -124 | -58 | -70 | 13 |
| March(2012) | *Suaeda vera* | Stiffkey | 52.98 | 0°55′E | 1 | Amaranthaceae | Dictyledoneae | succulent | C3 | -98 | -58 | -42 | 13 |
| May(2012) | *Atriplex portulacoides* | Stiffkey | 52.98 | 0°55′E | 1 | Amaranthaceae | Dictyledoneae | succulent | C3 | -150 | -58 | -98 | 13 |
| May(2012) | *Limonium vulgare* | Stiffkey | 52.98 | 0°55′E | 1 | Plumbaginaceae | Dictyledoneae | herb | C3 | -122 | -58 | -68 | 13 |
| May(2012) | *Salicornia Europaea* | Stiffkey | 52.98 | 0°55′E | 1 | Amaranthaceae | Dictyledoneae | succulent | C3 | -132 | -58 | -79 | 13 |
| May(2012) | *Suaeda vera* | Stiffkey | 53 | 0°55′E | 1 | Amaranthaceae | Dictyledoneae | succulent | C3 | -127 | -58 | -73 | 13 |
| August(2012) | *Atriplex portulacoides* | Stiffkey | 53 | 0°55′E | 1 | Amaranthaceae | Dictyledoneae | succulent | C3 | -153 | -58 | -101 | 13 |
| August(2012) | *Limonium vulgare* | Stiffkey | 53 | 0°55′E | 1 | Plumbaginaceae | Dictyledoneae | herb | C3 | -115 | -58 | -61 | 13 |
| August(2012) | *Salicornia Europaea* | Stiffkey | 53 | 0°55′E | 1 | Amaranthaceae | Dictyledoneae | succulent | C3 | -131 | -58 | -77 | 13 |
| August(2012) | *Suaeda vera* | Stiffkey | 53 | 0°55′E | 1 | Amaranthaceae | Dictyledoneae | succulent | C3 | -129 | -58 | -75 | 13 |
| Sept.(2012) | *Atriplex portulacoides* | Stiffkey | 53 | 0°55′E | 1 | Amaranthaceae | Dictyledoneae | succulent | C3 | -166 | -58 | -115 | 13 |
| Sept.(2012) | *Limonium vulgare* | Stiffkey | 53 | 0°55′E | 1 | Plumbaginaceae | Dictyledoneae | herb | C3 | -125 | -58 | -71 | 13 |
| Sept.(2012) | *Salicornia Europaea* | Stiffkey | 53 | 0°55′E | 1 | Amaranthaceae | Dictyledoneae | succulent | C3 | -127 | -58 | -73 | 13 |
| Sept.(2012) | *Suaeda vera* | Stiffkey | 53 | 0°55′E | 1 | Amaranthaceae | Dictyledoneae | succulent | C3 | -141 | -58 | -88 | 13 |
| April(2010) | *Populus angustifolia* | Utah | 40.6 | 111°43′W | 1892 | Salicaceae | Dictyledoneae | tree | C3 | -269 | -105 | | 14 |
| May(2010) | *Populus angustifolia* | Utah | 40.6 | 111°43′W | 1892 | Salicaceae | Dictyledoneae | tree | C3 | -270 | -105 | | 14 |
| May(2010) | *Populus angustifolia* | Utah | 40.6 | 111°43′W | 1892 | Salicaceae | Dictyledoneae | tree | C3 | -261 | -105 | | 14 |
| May(2010) | *Populus angustifolia* | Utah | 40.6 | 111°43′W | 1892 | Salicaceae | Dictyledoneae | tree | C3 | -246 | -105 | -158 | 14 |
| May(2010) | *Populus angustifolia* | Utah | 40.6 | 111°43′W | 1892 | Salicaceae | Dictyledoneae | tree | C3 | -227 | -105 | -136 | 14 |
| May(2010) | *Populus angustifolia* | Utah | 40.6 | 111°43′W | 1892 | Salicaceae | Dictyledoneae | tree | C3 | -211 | -105 | -118 | 14 |
| June(2010) | *Populus angustifolia* | Utah | 40.6 | 111°43′W | 1892 | Salicaceae | Dictyledoneae | tree | C3 | -210 | -105 | -117 | 14 |
| June(2010) | *Populus angustifolia* | Utah | 40.6 | 111°43′W | 1892 | Salicaceae | Dictyledoneae | tree | C3 | -223 | -105 | -132 | 14 |
| June(2010) | *Populus angustifolia* | Utah | 40.6 | 111°43′W | 1892 | Salicaceae | Dictyledoneae | tree | C3 | -224 | -105 | -133 | 14 |
| June(2010) | *Populus angustifolia* | Utah | 40.6 | 111°43′W | 1892 | Salicaceae | Dictyledoneae | tree | C3 | -216 | -105 | -124 | 14 |
| July(2010) | *Populus angustifolia* | Utah | 40.6 | 111°43′W | 1892 | Salicaceae | Dictyledoneae | tree | C3 | -218 | -105 | -126 | 14 |
| August(2010) | *Populus angustifolia* | Utah | 40.6 | 111°43′W | 1892 | Salicaceae | Dictyledoneae | tree | C3 | -217 | -105 | -125 | 14 |
| August(2010) | *Populus angustifolia* | Utah | 40.6 | 111°43′W | 1892 | Salicaceae | Dictyledoneae | tree | C3 | -222 | -105 | -131 | 14 |
| Sept.(2010) | *Populus angustifolia* | Utah | 40.6 | 111°43′W | 1892 | Salicaceae | Dictyledoneae | tree | C3 | -219 | -105 | -127 | 14 |
| Oct.(2010) | *Populus angustifolia* | Utah | 40.6 | 111°43′W | 1892 | Salicaceae | Dictyledoneae | tree | C3 | -217 | -105 | -125 | 14 |

| | | | | | | | | | | | | | |
|---|---|---|---|---|---|---|---|---|---|---|---|---|---|
| August(2006) | *Platanus occidentalis* | Hollis | 42.8 | 71°35′W | 424 | Platanaceae | Dictyledoneae | tree | C3 | -178 | -68 | -118 | 15 |
| August(2006) | *Acer rubrum* | Hollis | 42.8 | 71°35′W | 424 | Aceraceae | Dictyledoneae | tree | C3 | -181 | -68 | -121 | 15 |
| August(2006) | *Acer rubrum* | Framingham | 42.3 | 71°28′W | 269.2 | Aceraceae | Dictyledoneae | tree | C3 | -177 | -64 | -121 | 15 |
| August(2006) | *Acer rubrum* | Douglas | 42 | 71°47′W | 685.5 | Aceraceae | Dictyledoneae | tree | C3 | -175 | -69 | -114 | 15 |
| August(2006) | *Acer rubrum* | Coventry | 41.8 | 72°17′W | 301 | Aceraceae | Dictyledoneae | tree | C3 | -168 | -62 | -113 | 15 |
| August(2006) | *Platanus occidentalis* | Coventry | 41.8 | 72°17′W | 301 | Platanaceae | Dictyledoneae | tree | C3 | -186 | -62 | -132 | 15 |
| August(2006) | *Acer rubrum* | Burlington | 41.8 | 72°58′W | 1044.5 | Aceraceae | Dictyledoneae | tree | C3 | -180 | -73 | -115 | 15 |
| August(2006) | *Acer rubrum* | Perkasie | 40.4 | 75°17′W | 544 | Aceraceae | Dictyledoneae | tree | C3 | -163 | -60 | -110 | 15 |
| August(2006) | *Acer rubrum* | Alexandria | 38.7 | 77°7′W | 97 | Aceraceae | Dictyledoneae | tree | C3 | -154 | -48 | -111 | 15 |
| August(2006) | *Platanus occidentalis* | Alexandria | 38.7 | 77°7′W | 97 | Platanaceae | Dictyledoneae | tree | C3 | -165 | -48 | -123 | 15 |
| August(2006) | *Acer rubrum* | King George | 38.3 | 77°9′W | 102.75 | Aceraceae | Dictyledoneae | tree | C3 | -152 | -46 | -111 | 15 |
| August(2006) | *Acer rubrum* | St Stephens Church | 37.8 | 77°7′W | 108.25 | Aceraceae | Dictyledoneae | tree | C3 | -140 | -45 | -99 | 15 |
| August(2006) | *Acer rubrum* | Chesterfield | 37.2 | 77°35′W | 292.25 | Aceraceae | Dictyledoneae | tree | C3 | -158 | -47 | -116 | 15 |
| August(2006) | *Acer rubrum* | Lawrenceville | 36.8 | 77°55′W | 357.6 | Aceraceae | Dictyledoneae | tree | C3 | -157 | -46 | -116 | 15 |
| August(2006) | *Acer rubrum* | Raleigh | 35.99 | 78°37′W | 199.5 | Aceraceae | Dictyledoneae | tree | C3 | -153 | -41 | -117 | 15 |
| August(2006) | *Acer rubrum* | Lillington | 35.5 | 78°55′W | 390 | Aceraceae | Dictyledoneae | tree | C3 | -142 | -42 | -104 | 15 |
| August(2006) | *Acer rubrum* | Dillon | 34.2 | 79°16′W | 93 | Aceraceae | Dictyledoneae | tree | C3 | -144 | -35 | -113 | 15 |
| August(2006) | *Platanus occidentalis* | Olanta | 33.9 | 79°59′W | 197.17 | Platanaceae | Dictyledoneae | tree | C3 | -140 | -35 | -109 | 15 |
| August(2006) | *Acer rubrum* | Santee | 33.5 | 80°29′W | 219.67 | Aceraceae | Dictyledoneae | tree | C3 | -136 | -35 | -105 | 15 |
| August(2006) | *Acer rubrum* | Millen | 32.5 | 81°58′W | 197.17 | Aceraceae | Dictyledoneae | tree | C3 | -123 | -32 | -94 | 15 |
| Sept.(2012) | *Bothriochloa ischaemum* | Lantian | 34.3 | 109°7′E | 619 | Poaceae | Monocotyledoneae | grass | C4 | -207 | -50 | -165 | 1 |
| Sept.(2012) | *Stipa bungeana* | Lantian | 34.45 | 109°18′E | 619 | Poaceae | Monocotyledoneae | grass | C3 | -222 | -50 | -181 | 1 |
| Sept.(2012) | *Agropyron cristatum* | Lantian | 34.45 | 109°18′E | 619 | Poaceae | Monocotyledoneae | grass | | -185 | -50 | -142 | 1 |
| | *Agrostis matsumurae* | Xi'an | 34.4 | 108°56′E | 384 | Poaceae | Monocotyledoneae | grass | | -179 | -47 | -139 | 1 |
| | *Lolium perenne* | Xi'an | 34.4 | 108°56′E | 384 | Poaceae | Monocotyledoneae | grass | | -182 | -47 | -142 | 1 |
| May(2013） | *Stipa bungeana* | Heshui | 36 | 108°06′E | 1299 | Poaceae | Monocotyledoneae | grass | C3 | -183 | -59 | -131 | 2 |
| May(2013） | *Stipa bungeana* | Heshui | 36 | 108°06′E | 1252 | Poaceae | Monocotyledoneae | grass | C3 | -183 | -59 | -132 | 2 |
| May(2013） | *Bothriochloa ischaemum* | Heshui | 36 | 108°06′E | 1252 | Poaceae | Monocotyledoneae | grass | C4 | -197 | -59 | -147 | 2 |
| May(2013） | *Stipa bungeana* | Heshui | 36 | 108°06′E | 1246 | Poaceae | Monocotyledoneae | grass | C3 | -171 | -59 | -119 | 2 |
| May(2013） | *Stipa bungeana* | Heshui | 36 | 108°06′E | 1230 | Poaceae | Monocotyledoneae | grass | C3 | -179 | -59 | -128 | 2 |
| May(2013） | *Stipa bungeana* | Heshui | 36 | 108°06′E | 1210 | Poaceae | Monocotyledoneae | grass | C3 | -173 | -59 | -121 | 2 |
| May(2013） | *Stipa bungeana* | Heshui | 36 | 108°06′E | 1203 | Poaceae | Monocotyledoneae | grass | C3 | -181 | -59 | -129 | 2 |
| May(2013） | *Stipa bungeana* | Heshui | 36 | 108°06′E | 1196 | Poaceae | Monocotyledoneae | grass | C3 | -185 | -59 | -134 | 2 |
| May(3013） | *Bothriochloa ischaemum* | Heshui | 36 | 108°06′E | 1257 | Poaceae | Monocotyledoneae | grass | C4 | -189 | -59 | -138 | 2 |
| May(3013） | *Stipa bungeana* | Heshui | 36 | 108°06′E | 1257 | Poaceae | Monocotyledoneae | grass | C3 | -196 | -59 | -146 | 2 |
| May(3013） | *Bothriochloa ischaemum* | Heshui | 36.1 | 108°06′E | 1240 | Poaceae | Monocotyledoneae | grass | C4 | -192 | -59 | -141 | 2 |
| May(3013） | *Bothriochloa ischaemum* | Heshui | 36.1 | 108°06′E | 1225 | Poaceae | Monocotyledoneae | grass | C4 | -203 | -59 | -153 | 2 |
| May(3013） | *Bothriochloa ischaemum* | Heshui | 36.1 | 108°06′E | 1214 | Poaceae | Monocotyledoneae | grass | C4 | -202 | -59 | -152 | 2 |
| May(3013） | *Bothriochloa ischaemum* | Heshui | 36.1 | 108°06′E | 1198 | Poaceae | Monocotyledoneae | grass | C4 | -202 | -59 | -152 | 2 |
| May(3013） | *Stipa capillata* | Heshui | 36.1 | 108°06′E | 1195 | Poaceae | Monocotyledoneae | grass | C3 | -198 | -59 | -148 | 2 |
| Sept.(2013) | *Phragmites australis* | Heshui | 36.1 | 108°06′E | 1256 | Poaceae | Monocotyledoneae | grass | C3 | -239 | -59 | -191 | 2 |
| May(2013） | *Stipa bungeana* | Heshui | 36.1 | 108°06′E | 1281 | Poaceae | Monocotyledoneae | grass | C3 | -193 | -59 | -142 | 2 |
| Sept.(2013) | *Carex* | Heshui | 36.2 | 108°06′E | 1245 | Cyperaceae | Monocotyledoneae | grass | C3 | -215 | -59 | -165 | 2 |
| Sept.(2013) | *Phragmites australis* | Heshui | 36.2 | 108°06′E | 1234 | Poaceae | Monocotyledoneae | grass | C3 | -231 | -59 | -183 | 2 |
| Sept.(2013) | *Bothriochloa ischaemum* | Heshui | 36.2 | 108°06′E | 1219 | Poaceae | Monocotyledoneae | grass | C4 | -190 | -59 | -139 | 2 |
| Sept.(2013) | *Phragmites australis* | Heshui | 36.2 | 108°06′E | 1204 | Poaceae | Monocotyledoneae | grass | C3 | -210 | -59 | -161 | 2 |
| Sept.(2013) | *Stipa bungeana* | Heshui | 36.2 | 108°06′E | 1294 | Poaceae | Monocotyledoneae | grass | C3 | -197 | -59 | -147 | 2 |
| Sept.(2013) | *Phragmites australis* | Heshui | 36.2 | 108°06′E | 1277 | Poaceae | Monocotyledoneae | grass | C3 | -231 | -59 | -183 | 2 |
| Sept.(2013) | *Carex* | Heshui | 36.2 | 108°06′E | 1255 | Cyperaceae | Monocotyledoneae | grass | C3 | -207 | -59 | -157 | 2 |
| Sept.(2013) | *Phragmites australis* | Heshui | 36.2 | 108°06′E | 1228 | Poaceae | Monocotyledoneae | grass | C3 | -194 | -59 | -144 | 2 |
| Sept.(2013) | *Bothriochloa ischaemum* | Heshui | 36.2 | 108°06′E | 1228 | Poaceae | Monocotyledoneae | grass | C4 | -217 | -59 | -168 | 2 |
| Sept.(2013) | *Bothriochloa ischaemum* | Heshui | 36.2 | 108°06′E | 1213 | Poaceae | Monocotyledoneae | grass | C4 | -203 | -59 | -153 | 2 |
| Sept.(2013) | *Zea mays* | Heshui | 36.2 | 108°06′E | 1196 | Poaceae | Monocotyledoneae | grass | C4 | -184 | -59 | -133 | 2 |
| May(2013） | *Stipa bungeana* | Heshui | 36.2 | 108°06′E | 1266 | Poaceae | Monocotyledoneae | grass | C3 | -192 | -59 | -141 | 2 |
| | *Bothriochloa ischaemum* | Baishui | 35.2 | 109°35′E | 762 | Poaceae | Monocotyledoneae | grass | C4 | -170 | -53 | -124 | 3 |
| | *Cleistogenes squarrosa* | Ertuokeqi | 39 | 107°58′E | 1389 | Poaceae | Monocotyledoneae | grass | C4 | -161 | -62 | -106 | 3 |
| | *Stipa bungeana* | Ertuokeqi | 39 | 107°58′E | 1389 | Poaceae | Monocotyledoneae | grass | C3 | -167 | -62 | -112 | 3 |
| | *Achnatherum splendens* | Ertuokeqi | 39 | 107°58′E | 1389 | Poaceae | Monocotyledoneae | grass | C3 | -182 | -62 | -128 | 3 |
| | *Asparagus officinalis* | Ertuokeqi | 39 | 107°58′E | 1389 | Lilaceae | Monocotyledoneae | shrub | C3 | -164 | -62 | -109 | 3 |
| | *Stipa glareosa* | Ertuokeqi | 39 | 107°58′E | 1389 | Poaceae | Monocotyledoneae | grass | C3 | -159 | -62 | -103 | 3 |
| | *Stipa bungeana* | Huining | 35.6 | 105°03′E | 1921 | Poaceae | Monocotyledoneae | grass | C3 | -173 | -62 | -118 | 3 |
| | *Pennisetum flaccidum* | Lanzhou | 36 | 103°50′E | 1627 | Poaceae | Monocotyledoneae | grass | C4 | -195 | -56 | -147 | 3 |
| | *Bothriochloa ischaemum* | Luochuan | 35.5 | 109°30′E | 1132 | Poaceae | Monocotyledoneae | grass | C4 | -157 | -59 | -104 | 3 |
| | *Stipa grandis* | Neimeng | 40.7 | 111°45′E | 1437 | Poaceae | Monocotyledoneae | grass | C3 | -205 | -71 | -144 | 3 |
| | *Cleistogenes squarrosa* | Pengyang | 35.8 | 106°38′E | 1486 | Poaceae | Monocotyledoneae | grass | C4 | -178 | -59 | -126 | 3 |

| | | | | | | | | | | | | | |
|---|---|---|---|---|---|---|---|---|---|---|---|---|---|
| | *Pennisetum flaccidum* | Yanan | 36.6 | 109°29'E | 1061 | Poaceae | Monocotyledoneae | grass | C4 | -117 | -58 | | 3 |
| | *Borthriochloa ischaemum* | Yanan | 36.6 | 109°29'E | 1061 | Poaceae | Monocotyledoneae | grass | C4 | -152 | -58 | -100 | 3 |
| | *Stipa bungeana* | Yanan | 36.6 | 109°29'E | 1061 | Poaceae | Monocotyledoneae | grass | C3 | -154 | -58 | -102 | 3 |
| | *Stipa grandis* | Yanan | 36.6 | 109°29'E | 1061 | Poaceae | Monocotyledoneae | grass | C3 | -146 | -58 | -93 | 3 |
| | *Cleistogenes Keng* | Yuxian | 37 | 113°02'E | 1202 | Poaceae | Monocotyledoneae | grass | C4 | -204 | -65 | -149 | 4 |
| | *Stipa bungeana* | Yuxian | 37 | 113°02'E | 1202 | Poaceae | Monocotyledoneae | grass | C3 | -218 | -65 | -164 | 4 |
| | *Bothriochloa ischaemum* | Luochuan | 35.7 | 109°30'E | 1132 | Poaceae | Monocotyledoneae | grass | C4 | -160 | -59 | -107 | 4 |
| | *Cleistogenes Keng* | Luochuan | 35.7 | 109°30'E | 1132 | Poaceae | Monocotyledoneae | grass | C4 | -207 | -59 | -157 | 4 |
| | *Themeda triandra* | Luochuan | 35.7 | 109°30'E | 1132 | Poaceae | Monocotyledoneae | grass | C4 | -192 | -59 | -141 | 4 |
| | *Stipa bungeana* | Luochuan | 35.7 | 109°30'E | 1132 | Poaceae | Monocotyledoneae | grass | C3 | -168 | -59 | -116 | 4 |
| | *Stipa bungeana* | Ruicheng | 34.6 | 110°25'E | 796 | Poaceae | Monocotyledoneae | grass | C3 | -179 | -54 | -132 | 4 |
| | *Cleistogenes Keng* | Ruicheng | 34.4 | 110°25'E | 796 | Poaceae | Monocotyledoneae | grass | C4 | -179 | -54 | -132 | 4 |
| | *Bromus inermis* | Boulder | 40 | 105°16'W | 1468 | Poaceae | Monocotyledoneae | grass | C3 | -273 | -91 | -200 | 5 |
| | *Andropogon gerardii* | Boulder | 40 | 105°16'W | 1468 | Poaceae | Monocotyledoneae | grass | C4 | -238 | -91 | -162 | 5 |
| | *Schizachyrium scoparium* | Boulder | 40 | 105°16'W | 1468 | Poaceae | Monocotyledoneae | grass | C4 | -253 | -91 | -178 | 5 |
| | *Koeleria pyramidata* | Cottonwood | 43.9 | 101°52'W | 1549 | Poaceae | Monocotyledoneae | grass | C3 | -261 | -97 | -182 | 5 |
| | *Bromus inermis* | Cottonwood | 43.9 | 101°52'W | 1549 | Poaceae | Monocotyledoneae | grass | C3 | -238 | -97 | -156 | 5 |
| | *Buchloe dactyloides* | Cottonwood | 43.9 | 101°52'W | 1549 | Poaceae | Monocotyledoneae | grass | C4 | -219 | -97 | -135 | 5 |
| | *Schizachyrium scoparium* | Cottonwood | 43.9 | 101°52'W | 1549 | Poaceae | Monocotyledoneae | grass | C4 | -233 | -97 | -151 | 5 |
| | *Aristida longiseta* | Cottonwood | 43.9 | 101°52'W | 1549 | Poaceae | Monocotyledoneae | grass | C4 | -196 | -97 | -110 | 5 |
| | *Bouteloua curtipendula* | Cottonwood | 43.9 | 101°52'W | 1549 | Poaceae | Monocotyledoneae | grass | C4 | -213 | -97 | -128 | 5 |
| | *Agropyron smithii* | Cottonwood | 43.9 | 101°52'W | 1549 | Poaceae | Monocotyledoneae | grass | C3 | -244 | -97 | -163 | 5 |
| | *Andropogon gerardii* | Hays | 38.8 | 99°20'W | 3093 | Poaceae | Monocotyledoneae | grass | C4 | -213 | -99 | -127 | 5 |
| | *Sorghastrum nutans* | Hays | 38.8 | 99°20'W | 3093 | Poaceae | Monocotyledoneae | grass | C4 | -196 | -99 | -108 | 5 |
| | *Panicum virgatum* | Hays | 38.8 | 99°20'W | 3093 | Poaceae | Monocotyledoneae | grass | C4 | -212 | -99 | -125 | 5 |
| | *Agropyron smithii* | Hays | 38.8 | 99°20'W | 3093 | Poaceae | Monocotyledoneae | grass | C3 | -211 | -99 | -124 | 5 |
| | *Stipa viridula* | Mabdan | 46.7 | 100°55'W | 2623 | Poaceae | Monocotyledoneae | grass | C3 | -239 | -120 | -135 | 5 |
| | *Stipa comata* | Mabdan | 46.7 | 100°55'W | 2623 | Poaceae | Monocotyledoneae | grass | C3 | -222 | -120 | -116 | 5 |
| | *Schizachyrium scoparium* | Mabdan | 46.7 | 100°55'W | 2623 | Poaceae | Monocotyledoneae | grass | C4 | -228 | -120 | -123 | 5 |
| | *Calamovilfa longifolia* | Mabdan | 46.7 | 100°55'W | 2623 | Poaceae | Monocotyledoneae | grass | c4 | -216 | -120 | -109 | 5 |
| | *Bouteloua gracilis* | Mabdan | 46.7 | 100°55'W | 2623 | Poaceae | Monocotyledoneae | grass | C4 | -220 | -120 | -114 | 5 |
| | *Agropyron smithii* | Mabdan | 46.7 | 100°55'W | 2623 | Poaceae | Monocotyledoneae | grass | C3 | -251 | -120 | -149 | 5 |
| | *Elymus canadensis* | Manhattan | 39.2 | 96°35'W | 4131 | Poaceae | Monocotyledoneae | grass | C3 | -234 | -109 | -140 | 5 |
| | *Poa pratensis* | Manhattan | 39.2 | 96°35'W | 4131 | Poaceae | Monocotyledoneae | grass | C3 | -220 | -109 | -125 | 5 |
| | *Andropogon gerardii* | Manhattan | 39.2 | 96°35'W | 4131 | Poaceae | Monocotyledoneae | grass | C4 | -205 | -109 | -108 | 5 |
| | *Schizachyrium scoparium* | Manhattan | 39.2 | 96°35'W | 4131 | Poaceae | Monocotyledoneae | grass | C4 | -196 | -109 | -98 | 5 |
| | *Sorghastrum nutans* | Manhattan | 39.2 | 96°35'W | 4131 | Poaceae | Monocotyledoneae | grass | C4 | -197 | -109 | -99 | 5 |
| | *Stipa comata* | Nunn | 40.7 | 104°47'W | 1307 | Poaceae | Monocotyledoneae | grass | C3 | -210 | -90 | -132 | 5 |
| | *Bouteloua gracilis* | Nunn | 40.7 | 104°47'W | 1307 | Poaceae | Monocotyledoneae | grass | C4 | -189 | -90 | -109 | 5 |
| | *Agropyron smithii* | Nunn | 40.7 | 104°47'W | 1307 | Poaceae | Monocotyledoneae | grass | C3 | -214 | -90 | -136 | 5 |
| | *Stipa comata* | Socorro Country | 34 | 106°53'W | 1234 | Poaceae | Monocotyledoneae | grass | C3 | -167 | -71 | -103 | 5 |
| | *Bouteloua* | Socorro Country | 34 | 106°53'W | 1234 | Poaceae | Monocotyledoneae | grass | C4 | -136 | -71 | | 5 |
| | *Oryzopsis hymenoides* | Socorro Country | 34 | 106°53'W | 1234 | Poaceae | Monocotyledoneae | grass | C3 | -187 | -71 | -125 | 5 |
| | *Bouteloua gracilis* | Socorro Country | 34 | 106°53'W | 1234 | Poaceae | Monocotyledoneae | grass | C4 | -190 | -71 | -128 | 5 |
| | *Stipa viridula* | Woodworth | 47 | 99°18'W | 2243 | Poaceae | Monocotyledoneae | grass | C3 | -232 | -113 | -134 | 5 |
| | *Poa pratensis* | Woodworth | 47 | 99°18'W | 2243 | Poaceae | Monocotyledoneae | grass | C3 | -247 | -113 | -151 | 5 |
| | *Stipa comata* | Woodworth | 47 | 99°18'W | 2243 | Poaceae | Monocotyledoneae | grass | C3 | -231 | -113 | -133 | 5 |
| | *Bromus inermis* | Woodworth | 47 | 99°18'W | 2243 | Poaceae | Monocotyledoneae | grass | C3 | -275 | -113 | -183 | 5 |
| | *Andropogon gerardii* | Woodworth | 47 | 99°18'W | 2243 | Poaceae | Monocotyledoneae | grass | C4 | -225 | -113 | -126 | 5 |
| | *Schizachyrium scoparium* | Woodworth | 47 | 99°18'W | 2243 | Poaceae | Monocotyledoneae | grass | C4 | -223 | -113 | -124 | 5 |
| August(2005) | *Smilax rotundifolia L.* | Blood pond | 42 | 71°58'W | 212 | Smilacaceae | Monocotyledoneae | vine | C3 | -202 | -62 | -149 | 6 |
| August(2005) | *Typha latifolia L.* | Blood pond | 42 | 71°58'W | 212 | Typhaceae | Monocotyledoneae | herb | C3 | -195 | -62 | -142 | 6 |
| August(2005) | *Agropyron sp.* | Blood pond | 42 | 71°58'W | 212 | Poaceae | Monocotyledoneae | grass | C3 | -231 | -62 | -180 | 6 |
| August(2005) | *Dactylis glomerata L.* | Blood pond | 42 | 71°58'W | 212 | Poaceae | Monocotyledoneae | grass | C3 | -218 | -62 | -166 | 6 |
| August(2005) | *Juncus tenuis Willd.* | Blood pond | 42 | 71°58'W | 212 | Juncaceae | Monocotyledoneae | grass | C3 | -217 | -62 | -165 | 6 |
| August(2005) | *Phleum pratense L.* | Blood pond | 42 | 71°58'W | 212 | Poaceae | Monocotyledoneae | grass | C3 | -226 | -62 | -175 | 6 |
| August(2005) | *Poaceae* | Blood pond | 42 | 71°58'W | 212 | Poaceae | Monocotyledoneae | grass | C3 | -232 | -62 | -181 | 6 |
| August(2005) | *Alopecurus sp. L.* | Blood pond | 42 | 71°58'W | 212 | Poaceae | Monocotyledoneae | grass | C3 | -186 | -62 | -132 | 6 |
| August(2005) | *Lemna sp. L.* | Blood pond | 42 | 71°58'W | 212 | Araceae | Monocotyledoneae | grass | C3 | -200 | -62 | -147 | 6 |
| | *Puccinellia angustata(R. Br.)* | Axel Heiberg Island | 79.9 | 89°01'W | 357 | Poaceae | Monocotyledoneae | grass | C3 | -301 | -197 | -130 | 8 |
| | *Alopecurus alpinus J. E.Simith* | Axel Heiberg Island | 79.9 | 89°01'W | 357 | Poaceae | Monocotyledoneae | grass | C3 | -298 | -197 | -126 | 8 |
| | *Poa trivialis L.* | Denali | 63.68 | 148°54'W | 551 | Poaceae | Monocotyledoneae | grass | C3 | -294 | -149 | -170 | 8 |
| | *Hierochloe hirta(Schrank) Borbas* | Helsinki | 60.2 | 24°56'E | 16 | Poaceae | Monocotyledoneae | grass | C3 | -213 | -86 | -139 | 8 |
| | *Poa pratensis L.* | Helsinki | 60.2 | 24°56'E | 16 | Poaceae | Monocotyledoneae | grass | C3 | -244 | -86 | -173 | 8 |
| | *Triticum aestivum L.* | Stockholm | 59.35 | 18°02'E | 39 | Poaceae | Monocotyledoneae | grass | C3 | -171 | -81 | -98 | 8 |

| | | | | | | | | | | | | | |
|---|---|---|---|---|---|---|---|---|---|---|---|---|---|
| | *Phragmites australis(Cav.) Trin. Ex Steud.* | Stockholm | 59.35 | 18°02′E | 39 | Poaceae | Monocotyledoneae | herb | C3 | -199 | -81 | -128 | 8 |
| | *Brachypodium sylvaticum(Huds.) Beauv.* | Stockholm | 59.35 | 18°02′E | 39 | Poaceae | Monocotyledoneae | grass | C3 | -224 | -81 | -156 | 8 |
| | *Arundo donax L.* | Stockholm | 59.35 | 18°02′E | 39 | Poaceae | Monocotyledoneae | grass | C3 | -197 | -81 | -126 | 8 |
| Oct.(1999) | *Phragmites communis* | Gunma-Japan | 36.4 | 139°37′E | 114 | Poaceae | Monocotyledoneae | grass | C3 | -206 | -50 | -164 | 9 |
| Oct.(2000) | *Miscanthus sinensis* | Gunma-Japan | 36.4 | 139°37′E | 114 | Poaceae | Monocotyledoneae | grass | C4 | -186 | -50 | -143 | 9 |
| Oct.(2000) | *Vallisneria asiatica* | Gunma-Japan | 36.4 | 139°37′E | 114 | Hydrocharitaceae | Monocotyledoneae | grass | C3 | -167 | -50 | -123 | 9 |
| Oct.(2000) | *Potamogeton perfoliatus* | Gunma-Japan | 36.4 | 139°37′E | 114 | Potamogetonaceae | Monocotyledoneae | grass | C3 | -202 | -50 | -160 | 9 |
| Oct.(2000) | *Hydrilla verticillata* | Gunma-Japan | 36.4 | 139°37′E | 114 | Hydrocharitaceae | Monocotyledoneae | grass | C3 | -190 | -50 | -147 | 9 |
| March(2000) | *Saccharum officinarum* | Okinawa-Japan | 27 | 142°11′E | 47 | Poaceae | Monocotyledoneae | grass | C4 | -188 | -36 | -158 | 9 |
| Oct.(1997) | *Saccharum officinarum* | Tainland | 15.7 | 100°59′E | 114 | Poaceae | Monocotyledoneae | grass | C4 | -173 | -40 | -139 | 9 |
| Oct.(1997) | *Sorghum bicolor* | Tainland | 15.7 | 100°59′E | 114 | Poaceae | Monocotyledoneae | grass | C4 | -171 | -40 | -136 | 9 |
| Oct.(1997) | *Ananas comsus* | Tainland | 15.7 | 100°59′E | 114 | Bromeliaceae | Monocotyledoneae | grass | CAM | -190 | -40 | -156 | 9 |
| July(1998) | *Zea mays* | Tokyo-Japan | 35.6 | 139°30′E | 37 | Poaceae | Monocotyledoneae | grass | C4 | -153 | -48 | -110 | 9 |
| Sept.(1998) | *Zoysia japonica* | Tokyo-Japan | 35.6 | 139°30′E | 37 | Poaceae | Monocotyledoneae | grass | C4 | -176 | -48 | -134 | 9 |
| Nov.(1998) | *Colocasia esculenta* | Tokyo-Japan | 35.6 | 139°30′E | 37 | Araceae | Monocotyledoneae | grass | CAM | -196 | -48 | -155 | 9 |
| Dec.(1998) | *Lycoris radiata* | Tokyo-Japan | 35.6 | 139°30′E | 37 | Amaryllidoideae | Monocotyledoneae | grass | CAM | -188 | -48 | -147 | 9 |
| Sept.(1999) | *Miscanthus sinensis* | Tokyo-Japan | 35.6 | 139°30′E | 37 | Poaceae | Monocotyledoneae | grass | C4 | -173 | -48 | -131 | 9 |
| April(2005) | *Hordeum vulgare L.* | Switzerland | 47.2 | 7°44′E | 452 | Poaceae | Monocotyledoneae | grass | C3 | -190 | -56 | -142 | 12 |
| April(2005) | *Hordeum vulgare L.* | Switzerland | 47.2 | 7°44′E | 452 | Poaceae | Monocotyledoneae | grass | C3 | -195 | -56 | -147 | 12 |
| April(2005) | *Hordeum vulgare L.* | Switzerland | 47.2 | 7°44′E | 452 | Poaceae | Monocotyledoneae | grass | C3 | -207 | -56 | -160 | 12 |
| April(2005) | *Hordeum vulgare L.* | Switzerland | 47.2 | 7°44′E | 452 | Poaceae | Monocotyledoneae | grass | C3 | -204 | -56 | -157 | 12 |
| April(2005) | *Hordeum vulgare L.* | Switzerland | 47.2 | 7°44′E | 452 | Poaceae | Monocotyledoneae | grass | C3 | -212 | -56 | -165 | 12 |
| May(2005) | *Hordeum vulgare L.* | Switzerland | 47.2 | 7°44′E | 452 | Poaceae | Monocotyledoneae | grass | C3 | -199 | -56 | -151 | 12 |
| May(2005) | *Hordeum vulgare L.* | Switzerland | 47.2 | 7°44′E | 452 | Poaceae | Monocotyledoneae | grass | C3 | -197 | -56 | -149 | 12 |
| May(2005) | *Hordeum vulgare L.* | Switzerland | 47.2 | 7°44′E | 452 | Poaceae | Monocotyledoneae | grass | C3 | -182 | -56 | -133 | 12 |
| May(2005) | *Hordeum vulgare L.* | Switzerland | 47.2 | 7°44′E | 452 | Poaceae | Monocotyledoneae | grass | C3 | -190 | -56 | -142 | 12 |
| May(2005) | *Hordeum vulgare L.* | Switzerland | 47.2 | 7°44′E | 452 | Poaceae | Monocotyledoneae | grass | C3 | -203 | -56 | -156 | 12 |
| May(2005) | *Hordeum vulgare L.* | Switzerland | 47.3 | 7°44′E | 452 | Poaceae | Monocotyledoneae | grass | C3 | -203 | -56 | -156 | 12 |
| June(2005) | *Hordeum vulgare L.* | Switzerland | 47.3 | 7°44′E | 452 | Poaceae | Monocotyledoneae | grass | C3 | -196 | -56 | -148 | 12 |
| June(2005) | *Hordeum vulgare L.* | Switzerland | 47.3 | 7°44′E | 452 | Poaceae | Monocotyledoneae | grass | C3 | -191 | -56 | -143 | 12 |
| June(2005) | *Hordeum vulgare L.* | Switzerland | 47.3 | 7°44′E | 452 | Poaceae | Monocotyledoneae | grass | C3 | -192 | -56 | -144 | 12 |
| June(2005) | *Hordeum vulgare L.* | Switzerland | 47.3 | 7°44′E | 452 | Poaceae | Monocotyledoneae | grass | C3 | -193 | -56 | -145 | 12 |
| June(2005) | *Hordeum vulgare L.* | Switzerland | 47.3 | 7°44′E | 452 | Poaceae | Monocotyledoneae | grass | C3 | -194 | -56 | -146 | 12 |
| June(2005) | *Hordeum vulgare L.* | Switzerland | 47.3 | 7°44′E | 452 | Poaceae | Monocotyledoneae | grass | C3 | -197 | -56 | -149 | 12 |
| June(2005) | *Hordeum vulgare L.* | Switzerland | 47.3 | 7°44′E | 452 | Poaceae | Monocotyledoneae | grass | C3 | -200 | -56 | -153 | 12 |
| June(2011) | *Spartina anglica* | Stiffkey | 52.9 | 0°55′E | 1 | Poaceae | Monocotyledoneae | grass | C4 | -157 | -58 | -105 | 13 |
| June(2011) | *Triglochin maritima* | Stiffkey | 52.9 | 0°55′E | 1 | Juncaginaceae | Monocotyledoneae | herb | C3 | -133 | -58 | -80 | 13 |
| June(2011) | *Elytrigia atherica* | Stiffkey | 52.9 | 0°55′E | 1 | Poaceae | Monocotyledoneae | grass | C3 | -211 | -58 | -162 | 13 |
| June(2011) | *Phragmites* | Stiffkey | 52.9 | 0°55′E | 1 | Poaceae | Monocotyledoneae | reed | C3 | -179 | -58 | -128 | 13 |
| June(2011) | *Puccinella maritima* | Stiffkey | 52.9 | 0°55′E | 1 | | Monocotyledoneae | grass | C3 | -206 | -58 | -157 | 13 |
| June(2011) | *Triglochin maritima* | Stiffkey | 52.9 | 0°55′E | 1 | Juncaginaceae | Monocotyledoneae | herb | C3 | -135 | -58 | -82 | 13 |
| March(2012) | *Elytrigia atherica* | Stiffkey | 52.9 | 0°55′E | 1 | Poaceae | Monocotyledoneae | grass | C3 | -201 | -58 | -152 | 13 |
| March(2012) | *Spartina anglica* | Stiffkey | 52.9 | 0°55′E | 1 | Poaceae | Monocotyledoneae | grass | C4 | -137 | -58 | -84 | 13 |
| May(2012) | *Elytrigia atherica* | Stiffkey | 52.9 | 0°55′E | 1 | Poaceae | Monocotyledoneae | grass | C3 | -232 | -58 | -185 | 13 |
| May(2012) | *Phragmites* | Stiffkey | 52.8 | 0°55′E | 1 | Poaceae | Monocotyledoneae | reed | C3 | -178 | -58 | -127 | 13 |
| May(2012) | *Spartina anglica* | Stiffkey | 52.8 | 0°55′E | 1 | Poaceae | Monocotyledoneae | grass | C4 | -146 | -58 | -93 | 13 |
| August(2012) | *Elytrigia atherica* | Stiffkey | 52.8 | 0°55′E | 1 | Poaceae | Monocotyledoneae | grass | C3 | -235 | -58 | -188 | 13 |
| August(2012) | *Phragmites* | Stiffkey | 52.8 | 0°55′E | 1 | Poaceae | Monocotyledoneae | reed | C3 | -196 | -58 | -146 | 13 |
| August(2012) | *Spartina anglica* | Stiffkey | 52.8 | 0°55′E | 1 | Poaceae | Monocotyledoneae | grass | C4 | -163 | -58 | -111 | 13 |
| Sept.(2012) | *Elytrigia atherica* | Stiffkey | 52.8 | 0°55′E | 1 | Poaceae | Monocotyledoneae | grass | C3 | -230 | -58 | -183 | 13 |
| Sept.(2012) | *Phragmites* | Stiffkey | 52.8 | 0°55′E | 1 | Poaceae | Monocotyledoneae | reed | c3 | -206 | -58 | -157 | 13 |
| Sept.(2012) | *Spartina anglica* | Stiffkey | 52.8 | 0°55′E | 1 | Poaceae | Monocotyledoneae | grass | C4 | -163 | -58 | -111 | 13 |
| Sept.(2012) | *Artemisia* | Lantian | 34°15′N | 109°8′E | 619 | Asteraceae | | grass | | -224 | -50 | -184 | 1 |
| | *Artemisia* | Xi'an | 34°20′N | 108°56′E | 384 | | | | | -103 | -47 | -58 | 1 |
| May(2013） | *Bryophyta* | Heshui | 36°01′N | 108°06′E | 1299 | | | | | -214 | -59 | -165 | 2 |
| May(3013） | *Artemisia* | Heshui | 36°01′N | 108°06′E | 1198 | | | shrub | | -150 | -59 | -97 | 2 |
| | *Citibetica kom* | Ertuokeqi | 39°05′N | 107°58′E | 1389 | Leguminosae | | shrub | C3 | -165 | -62 | -110 | 3 |
| | *Sviciifolia hance* | Yanan | 36°35′N | 109°29′E | 1061 | Leguminosae | | shrub | C3 | -115 | -58 | -61 | 3 |
| | *no identification* | Sanya | 18°13′N | 109°30′E | 8 | | | grass | C4 | -171 | -36 | -140 | 4 |
| | *no identification* | Sanya | 18°13′N | 109°30′E | 8 | | | grass | C4 | -148 | -36 | -116 | 4 |
| | *no identification* | Sanya | 18°13′N | 109°30′E | 8 | | | grass | C3 | -155 | -36 | -123 | 4 |
| | *no identification* | Sanya | 18°13′N | 109°30′E | 8 | | | grass | C3 | -162 | -36 | -131 | 4 |
| | *no identification* | Sanya | 18°13′N | 109°30′E | 8 | | | grass | C4 | | -36 | 37 | 4 |
| | *no identification* | Sanya | 18°13′N | 109°30′E | 8 | | | grass | C4 | -175 | -36 | -144 | 4 |
| | *no identification* | Sanya | 18°13′N | 109°30′E | 8 | | | tree | c3 | -131 | -36 | -99 | 4 |

| | | | | | | | | | | | | | |
|---|---|---|---|---|---|---|---|---|---|---|---|---|---|
| | *no identification* | Sanya | 18°13′N | 109°30′E | 8 | | | tree | C3 | -144 | -36 | -112 | 4 |
| | *no identification* | Sanya | 18°13′N | 109°30′E | 8 | | | tree | C3 | -159 | -36 | -128 | 4 |
| | *no identification* | Sanya | 18°13′N | 109°30′E | 8 | | | shrub | C3 | -129 | -36 | -96 | 4 |
| | *no identification* | Sanya | 18°13′N | 109°30′E | 8 | | | tree | C3 | -134 | -36 | -102 | 4 |
| | *no identification* | Sanya | 18°13′N | 109°30′E | 8 | | | tree | C3 | -160 | -36 | -129 | 4 |
| Nov.(2007) | *Rhus laurina* | Topanga | 34°5′N | 118°35′W | 400 | | | shrub | C3 | -147 | -66 | -87 | 7 |
| | *Picea glauca(Moench) Voss* | Denali | 63°43′N | 148°54′W | 551 | Pinaceae | | tree | C3 | -229 | -149 | -94 | 8 |
| | *Equisetum pratense Ehrhart* | Sutton | 61°44′N | 148°56′W | 340 | Equisetaceae | | fern | C3 | -202 | -139 | -73 | 8 |
| Aug-Sept(2002) | *Cladonia* | Keitjoru(SWE) | 68°40′N | 21°30′E | 428 | Cladoniaceae | | grass | C3 | -207 | -109 | -110 | 10 |
| Aug-Sept(2002) | *Cladonia* | Tunturilampi(FIN) | 67°21′N | 27°10′E | 293 | Cladoniaceae | | grass | C3 | -190 | -99 | -101 | 10 |
| Aug-Sept(2002) | *Moss* | Syrjanalunen(FIN) | 61°11′N | 25°8′E | 156 | | | grass | C3 | -198 | -91 | -118 | 10 |
| Aug-Sept(2002) | *Sphagnum* | Tunturilampi(FIN) | 67°21′N | 27°10′E | 293 | Sphagnales | | grass | C3 | -235 | -99 | -151 | 10 |
| | | | | | | | | | | | | | |
| | *Ginkgo biloba L.* | Xi'an | 34.33 | 108.95 | 384 | Ginkgoaceae | Gymnosperm | tree | C3 | -133 | -47 | -90 | 1 |
| | *Metasequoia glyptostroboides* | Xi'an | 34.33 | 108.95 | 384 | Taxodiacceae | Gymnosperm | tree | C3 | -143 | -47 | -101 | 1 |
| | *Pinus tabulaeformis* | Huanglong | 34.15 | 106.82 | 1319 | Pinaceae | Gymnosperm | tree | C3 | -136 | -58 | -83 | 3 |
| | *Pinus tabulaeformis* | Huanglong | 34.15 | 106.82 | 1319 | Pinaceae | Gymnosperm | tree | C3 | -137 | -58 | -84 | 3 |
| August(2005) | *Pinus strobus L.(6m)* | Blood pond | 42.08 | -71.96 | 212 | Pinaceae | Gymnosperm | tree | C3 | -192 | -45 | -154 | 6 |
| August(2005) | *Pinus strobus L.(4.5m)* | Blood pond | 42.08 | -71.96 | 212 | Pinaceae | Gymnosperm | tree | C3 | -201 | -45 | -163 | 6 |
| August(2005) | *Pinus strobus L.(3m)* | Blood pond | 42.08 | -71.96 | 212 | Pinaceae | Gymnosperm | tree | C3 | -200 | -45 | -162 | 6 |
| Feb.(2007) | *Ephedra nevadensis* | Mojave | 34.8 | -115.67 | 1500 | Ephedraceae | Gymnosperm | shrub | C3 | -149 | -66 | -89 | 7 |
| | *Metasequoia glyptostroboides* | Uppusala | 59.93 | 17.63 | 26 | Cupressaceae | Gymnosperm | tree | C3 | -178 | -82 | -105 | 8 |
| May(1999) | *Cryptomeria japonica* | Gunma-Japan | 36.38 | 139.62 | 114 | Taxodiaceae | Gymnosperm | tree | C3 | -156 | -50 | -112 | 9 |
| Oct.(1999) | *Cryptomeria japonica* | Gunma-Japan | 36.38 | 139.62 | 114 | Taxodiaceae | Gymnosperm | tree | C3 | -168 | -50 | -124 | 9 |
| Oct.(1998) | *Chamaecyparis obtusa* | Tokyo-Japan | 35.68 | 139.5 | 37 | Cupressaceae | Gymnosperm | tree | C3 | -150 | -48 | -107 | 9 |
| Nov.(1998) | *Pinus thunbergii* | Tokyo-Japan | 35.68 | 139.5 | 37 | Pinaceae | Gymnosperm | tree | C3 | -164 | -48 | -122 | 9 |
| August(2006) | *Pinus strobus* | | 42.75 | -71.59 | 424 | Pinaceae | Gymnosperm | tree | C3 | -188 | -68 | -129 | 15 |
| July(2006) | *Juniperus virginiana* | | 42.32 | -71.46 | 269 | Cupressaceae | Gymnosperm | tree | C3 | -140 | -64 | -81 | 15 |
| August(2006) | *Pinus strobus* | | 42.32 | -71.46 | 269 | Pinaceae | Gymnosperm | tree | C3 | -176 | -64 | -120 | 15 |
| August(2006) | *Pinus strobus* | | 42.06 | -71.78 | 686 | Pinaceae | Gymnosperm | tree | C3 | -176 | -69 | -115 | 15 |
| August(2006) | *Pinus strobus* | | 41.78 | -72.28 | 301 | Pinaceae | Gymnosperm | tree | C3 | -193 | -62 | -140 | 15 |
| August(2006) | *Pinus strobus* | | 41.75 | -72.96 | 1045 | Pinaceae | Gymnosperm | tree | C3 | -171 | -73 | -106 | 15 |
| July(2006) | *Juniperus virginiana* | | 41.47 | -73.5 | 511 | Cupressaceae | Gymnosperm | tree | C3 | -140 | -64 | -81 | 15 |
| July(2006) | *Juniperus virginiana* | | 41.23 | -74.18 | 465 | Cupressaceae | Gymnosperm | tree | C3 | -144 | -62 | -87 | 15 |
| July(2006) | *Juniperus virginiana* | | 40.75 | -74.44 | 269 | Cupressaceae | Gymnosperm | tree | C3 | -131 | -57 | -78 | 15 |
| August(2006) | *Juniperus virginiana* | | 40.39 | -75.28 | 544 | Cupressaceae | Gymnosperm | tree | C3 | -135 | -60 | -80 | 15 |
| August(2006) | *Pinus strobus* | | 40.39 | -75.28 | 544 | Pinaceae | Gymnosperm | tree | C3 | -175 | -60 | -122 | 15 |
| July(2006) | *Juniperus virginiana* | | 38.76 | -77.12 | 97 | Cupressaceae | Gymnosperm | tree | C3 | -132 | -48 | -88 | 15 |
| July(2006) | *Juniperus virginiana* | | 38.34 | -77.15 | 103 | Cupressaceae | Gymnosperm | tree | C3 | -132 | -46 | -90 | 15 |
| July(2006) | *Juniperus virginiana* | | 37.81 | -77.12 | 108 | Cupressaceae | Gymnosperm | tree | C3 | -132 | -45 | -91 | 15 |
| August(2006) | *Pinus taeda* | | 37.36 | -77.59 | 292 | Pinaceae | Gymnosperm | tree | C3 | -180 | -46 | -140 | 15 |
| July(2006) | *Juniperus virginiana* | | 36.84 | -77.92 | 358 | Cupressaceae | Gymnosperm | tree | C3 | -122 | -46 | -80 | 15 |
| August(2006) | *Pinus taeda* | | 36.84 | -77.92 | 358 | Pinaceae | Gymnosperm | tree | C3 | -172 | -46 | -132 | 15 |
| July(2006) | *Juniperus virginiana* | | 36.44 | -78.37 | 367 | Cupressaceae | Gymnosperm | tree | C3 | -103 | -45 | -61 | 15 |
| August(2006) | *Pinus taeda* | | 36.44 | -78.37 | 367 | Pinaceae | Gymnosperm | tree | C3 | -175 | -45 | -136 | 15 |
| July(2006) | *Juniperus virginiana* | | 35.95 | -78.61 | 200 | Cupressaceae | Gymnosperm | tree | C3 | -135 | -41 | -98 | 15 |
| August(2006) | *Pinus taeda* | | 35.95 | -78.61 | 200 | Pinaceae | Gymnosperm | tree | C3 | -173 | -41 | -138 | 15 |
| July(2006) | *Juniperus virginiana* | | 35.46 | -78.91 | 390 | Cupressaceae | Gymnosperm | tree | C3 | -113 | -42 | -74 | 15 |
| August(2006) | *Pinus taeda* | | 35.46 | -78.91 | 390 | Pinaceae | Gymnosperm | tree | C3 | -153 | -42 | -116 | 15 |
| July(2006) | *Juniperus virginiana* | | 34.33 | -79.26 | 93 | Cupressaceae | Gymnosperm | tree | C3 | -143 | -35 | -112 | 15 |
| July(2006) | *Juniperus virginiana* | | 33.95 | -79.98 | 197 | Cupressaceae | Gymnosperm | tree | C3 | -136 | -35 | -105 | 15 |
| August(2006) | *Pinus taeda* | | 33.52 | -80.49 | 220 | Pinaceae | Gymnosperm | tree | C3 | -166 | -34 | -137 | 15 |
| July(2006) | *Juniperus virginiana* | | 33.06 | -81.09 | 123 | Cupressaceae | Gymnosperm | tree | C3 | -112 | -32 | -83 | 15 |
| July(2006) | *Juniperus virginiana* | | 32.88 | -81.96 | 197 | Cupressaceae | Gymnosperm | tree | C3 | -121 | -32 | -92 | 15 |
| July(2006) | *Juniperus virginiana* | | 30.19 | -84.37 | 96 | Cupressaceae | Gymnosperm | tree | C3 | -122 | -26 | -99 | 15 |
| July(2006) | *Juniperus virginiana* | | 30.03 | -84.39 | 13 | Cupressaceae | Gymnosperm | tree | C3 | -120 | -25 | -97 | 15 |

Refferences: 1. unpublished data; 2. publishing data; 3. Liu et al.,2006; 4. Liu and Huang,2008; 5. Smith and Freeman,2006; 6. Hou et al.,2007; 7. Feakins and Sessions,2010;

8. Yang et al.,2011; 9. Chikaraishi and Naraoka,2003; 10. Sachse et al.,2006; 11. Sachse et al.,2009; 12. Sachse et al., 2010; 13. Eley et al.,2014; 14. Tipple et al., 2013(PNAS);

15. Tipple et al.,2013(GCA)


2. Liu J.Z.,An Z.S., Liu W.G.,Wang Z.2014.Effects of soil water from the different terrain slopes seasns on leaf n-alkane δD values in modern plants. *Org. Geochem.*(in press).

3. Liu W.G.,Yang H.,Li L.,2006. Hydrogen isotopic compositions of n-alkanes from terrestrial plants correlate with their ecological life forms. *Oecologia* 150,330-338

4. Liu W.G.,Yang H.,2008. Mutiple controls for the variability of hydrogen isotopic compositions in higher plant n-alkane from modern ecosystems. *Glob. Change Biol.* 14,2166-2177

5. Smith F.A., Freeman K.H.,2006. Influence of physiology and climate on δD of leaf wax n-alkanes from C3 and C4 grasses. *Geochim. Cosmochim. Acta* 70,1172-1187

6. Hou J.Z., Andrea W.J.D., MacDonald D., Huang Y.S.,2007. Hydrogen isotopic variability in leaf waxes among terrestrial and aquatic plants around Blood Pond, Massachusetts (USA).

| |
|---|
| *Org. Geochem.* 38, 977-984 |
| 7. Feakins S.J., Sessions A.L.,2010. Controls on the D/H ratios of plant leaf waxes in an arid ecosystem. *Geochim. Cosmochim. Acta* 74, 2128-2141 |
| 8. Yang H., Liu W.G., Leng Q.,Hren M.T., Pagani M.,2011. Variation in n-alkane δD values from terrestrial plants at high latitude: Implications for paleoclimate reconstruction. |
| *Org. Geochem*. 42, 283-288 |
| 9. Chikaraishi Y., Naraoka H., 2003. Compound-specific δD-δ13C analyses of n-alkanes extracted from terrestrial and aquatic plants. *Phytochemistry* 63, 361-371 |
| 10. Sachse D., Badke J., Gleixner G.,2006. δD values of individual n-alkanes from terrestrial plants along a climatic gradient-Implications for the sedimentary biomarker record. |
| *Org. Geochem.* 37, 469-483 |
| 11. Sachse D., Kahmen A., Glexiner G.,2009. Significant seasonal varitaion in the hydrogen isotopic composition of leaf-wax lipids for two deciduous tree ecosystems (*Fagus sylvativa* |
| and *Acer pseudoplatanus*). *Org. Geochem.* 40, 732-742 |
| 12. Sachse D., Gleixner G., Wilkes H., Kahmen A.,2010. Leaf wax n-alkane δD values of field-grown barley reflect leaf water δD values at the time of leaf formation. *Geochim.* |
| *Cosmochim. Acta* 74, 6741-6750 |
| 13. Eley Y., Dawson L., Black S., Andrews J., Pedentchouk N., 2014. Understanding 2H/1H systematics of leaf wax n-alkanes in coastal plants at Stiffkey saltmarsh, Norfolk, UK. |
| Geochim. Cosmochim. Acta 128, 13-28 |
| 14. Tipple B., Berke M., Doman C.,Khachaturyan S., Ehleringer J., 2013. Leaf -wax n-alkanes record the plant-water environment at leaf flush. *Pro. Natl. Acad. Sci. USA* 110,2659-2664 |
| 15. Tipple B., Pagania M., 2013. Environmental control on eastern broadleaf forest species' leaf wax distributions and D/Hratios. *Geochim. Cosmochim. Acta* 111, 64-77 |

**Table S3** Measured δD values of leaf water and leaf wax along the leaf blade in *Rhleum palmatum L.* (dicot) and *Hierochloe glabra* (monocot) (‰, VSMOW).

| ID | Comments | Species | Latitude | Longitude | Altitude/m | Family | Plant taxonomy | Weight/g | Leaf water δD | $\delta D_{C29\ n\text{-}alkane}$ | $\varepsilon_{wax\text{-}lw}$ | Calculated $\varepsilon_{wax\text{-}lw}$ [a] | Entire leaf $\delta D_{C29\ n\text{-}alkane}$ |
|---|---|---|---|---|---|---|---|---|---|---|---|---|---|
| S-1 | tip | *Hierochloe glabra* | 35°14′36″ | 108°9′3″ | 410 | Poaceae | monocotyledonous | 0.171 | 37 | -176 | -205 | | |
| S-2 | | *Hierochloe glabra* | 35°14′36″ | 108°9′3″ | 410 | Poaceae | monocotyledonous | 0.231 | 25 | -187 | -207 | | |
| S-3 | base | *Hierochloe glabra* | 35°14′36″ | 108°9′3″ | 410 | Poaceae | monocotyledonous | 0.308 | 4 | -204 | -207 | -206 | -201 |
| S′-1 | tip | *Hierochloe glabra* | 35°14′36″ | 108°9′3″ | 410 | Poaceae | monocotyledonous | 0.215 | 35 | -177 | -205 | | |
| S′-2 | | *Hierochloe glabra* | 35°14′36″ | 108°9′3″ | 410 | Poaceae | monocotyledonous | 0.335 | 23 | -187 | -205 | | |
| S′-3 | base | *Hierochloe glabra* | 35°14′36″ | 108°9′3″ | 410 | Poaceae | monocotyledonous | 0.394 | 0 | -208 | -208 | | |
| P-1 | edge（tip） | *Rheum palmatum L.* | 35°14′36″ | 108°9′3″ | 410 | Polygonaceae | dicotyledonous | 0.184 | 9 | -180 | -187 | | |
| P-2 | | *Rheum palmatum L.* | 35°14′36″ | 108°9′3″ | 410 | Polygonaceae | dicotyledonous | 0.416 | 6 | -180 | -185 | | |
| P-3 | | *Rheum palmatum L.* | 35°14′36″ | 108°9′3″ | 410 | Polygonaceae | dicotyledonous | 0.392 | 9 | -180 | -187 | | |
| P-4 | | *Rheum palmatum L.* | 35°14′36″ | 108°9′3″ | 410 | Polygonaceae | dicotyledonous | 0.208 | 7 | -182 | -187 | | |
| P-5 | | *Rheum palmatum L.* | 35°14′36″ | 108°9′3″ | 410 | Polygonaceae | dicotyledonous | 0.309 | 8 | -184 | -191 | | |
| P-6 | edge（base） | *Rheum palmatum L.* | 35°14′36″ | 108°9′3″ | 410 | Polygonaceae | dicotyledonous | 0.208 | 7 | -183 | -189 | | |
| P-7 | | *Rheum palmatum L.* | 35°14′36″ | 108°9′3″ | 410 | Polygonaceae | dicotyledonous | 0.114 | 14 | -182 | -193 | -188 | -184 |
| P-8 | | *Rheum palmatum L.* | 35°14′36″ | 108°9′3″ | 410 | Polygonaceae | dicotyledonous | 0.121 | 5 | -188 | -192 | | |
| Q-1 | vein（tip） | *Rheum palmatum L.* | 35°14′36″ | 108°9′3″ | 410 | Polygonaceae | dicotyledonous | 0.185 | 8 | -188 | -194 | | |
| Q-2 | | *Rheum palmatum L.* | 35°14′36″ | 108°9′3″ | 410 | Polygonaceae | dicotyledonous | 0.188 | -11 | -195 | -186 | | |
| Q-3 | | *Rheum palmatum L.* | 35°14′36″ | 108°9′3″ | 410 | Polygonaceae | dicotyledonous | 0.212 | -15 | -196 | -184 | | |
| Q-4 | | *Rheum palmatum L.* | 35°14′36″ | 108°9′3″ | 410 | Polygonaceae | dicotyledonous | 0.127 | -20 | -197 | -180 | | |
| Q-5 | vein（base） | *Rheum palmatum L.* | 35°14′36″ | 108°9′3″ | 410 | Polygonaceae | dicotyledonous | 0.245 | -26 | -196 | -174 | | |

[a] Caculated $\varepsilon_{wax-lw} = \sum_{i=1}^{n} \varepsilon_i * m_i(\%)$. $\varepsilon_i$ represents fractionation factor between leaf wax $\delta D_{n\text{-alkane}}$ values and corresponding leaf water δD values at segmented i section along the leaf blade; $m_i(\%)$ represents percentage of weight at segmented i section.

Table S4 The measured and calculated hydrogen isotope composition of leaf water ($\delta D_{leaf\ water}$), leaf wax ($\delta D_{C29\ n\text{-}alkane}$) and corresponding $\varepsilon_{wax\text{-}lw}$ values from segmented leaves and entire leaf (‰).

| ID | Comments | Species | Latitude | Longitude | Altitude/m | Family | Plant taxonomy | Weight/g | $\delta D_{leaf\ water}$ | $\delta D_{C29\ n\text{-}alkane}$ | $\varepsilon_{wax\text{-}lw}$ | Caculated $\delta D_{C29\ n\text{-}alkane}$ | Caculated $\varepsilon_{wax\text{-}lw}$ | Entire leaf | | | $\Delta(\delta D_{C29\ n\text{-}alkane}-\delta D'_{C29\ n\text{-}alkane})$ | $\Delta(\varepsilon_{wax\text{-}lw}-\varepsilon'_{wax\text{-}lw})$ |
|---|---|---|---|---|---|---|---|---|---|---|---|---|---|---|---|---|---|---|
| | | | | | | | | | | | | | | $\delta D_{leaf\ water}$ | $\delta D'_{C29\ n\text{-}alkane}$ | $\varepsilon'_{wax\text{-}lw}$ | | |
| S'-1 | tip | *Carex tristachya* | 35°14′ 36″ | 108°9′ 3″ | 410 | Poaceae | Monocotyledonous | 0.086 | 2 | -177 | -178 | 0 | -181 | -20 | -192 | -175 | 2 | -6 |
| S'-2 | | *Carex tristachya* | 35°14′ 36″ | 108°9′ 3″ | 410 | Poaceae | Monocotyledonous | 0.139 | -11 | -187 | -178 | | | | | | | |
| S'-3 | base | *Carex tristachya* | 35°14′ 36″ | 108°9′ 3″ | 410 | Poaceae | Monocotyledonous | 0.136 | -20 | -202 | -186 | | | | | | | |
| | | | | | | | | | | | | | | | | | | |
| F-1 | tip | *Agropyron cristatum* | 35°14′ 36″ | 108°9′ 3″ | 371 | Poaceae | Monocotyledonous | 0.111 | 19 | -147 | -163 | -153 | -161 | -12 | -166 | -156 | 13 | -5 |
| F-2 | | *Agropyron cristatum* | 35°14′ 36″ | 108°9′ 3″ | 371 | Poaceae | Monocotyledonous | 0.196 | 17 | -169 | -183 | | | | | | | |
| F-3 | | *Agropyron cristatum* | 35°14′ 36″ | 108°9′ 3″ | 371 | Poaceae | Monocotyledonous | 0.28 | 8 | -138 | -145 | | | | | | | |
| F-4 | base | *Agropyron cristatum* | 35°14′ 36″ | 108°9′ 3″ | 371 | Poaceae | Monocotyledonous | 0.274 | 0 | -160 | -160 | | | | | | | |
| | | | | | | | | | | | | | | | | | | |
| Z-1 | edge | *Petasites japonicus* | 35°14′ 36″ | 108°9′ 3″ | 371 | Asteraceae | Dicotyledonous | 0.91 | -7 | -116 | -110 | -132 | -126 | -12 | -128 | -117 | -4 | -9 |
| Z-2 | | *Petasites japonicus* | 35°14′ 36″ | 108°9′ 3″ | 371 | Asteraceae | Dicotyledonous | 0.656 | -11 | -132 | -123 | | | | | | | |
| Z-3 | | *Petasites japonicus* | 35°14′ 36″ | 108°9′ 3″ | 371 | Asteraceae | Dicotyledonous | 0.763 | -13 | -141 | -130 | | | | | | | |
| Z-4 | center | *Petasites japonicus* | 35°14′ 36″ | 108°9′ 3″ | 371 | Asteraceae | Dicotyledonous | 0.483 | -13 | -149 | -138 | | | | | | | |
| | | | | | | | | | | | | | | | | | | |
| X-1 | tip | *Carpesium abrotanoides* | 35°14′ 36″ | 108°9′ 3″ | 371 | Asteraceae | Dicotyledonous | 0.103 | 8 | -157 | -164 | -148 | -146 | 3 | -146 | -148 | -2 | 2 |
| X-2 | | *Carpesium abrotanoides* | 35°14′ 36″ | 108°9′ 3″ | 371 | Asteraceae | Dicotyledonous | 0.127 | 2 | -149 | -151 | | | | | | | |
| X-3 | base | *Carpesium abrotanoides* | 35°14′ 36″ | 108°9′ 3″ | 371 | Asteraceae | Dicotyledonous | 0.111 | -9 | -149 | -141 | | | | | | | |
| Y-1 | edge | *Carpesium abrotanoides* | 35°14′ 36″ | 108°9′ 3″ | 371 | Asteraceae | Dicotyledonous | 0.181 | 8 | -142 | -149 | | | | | | | |
| Y-2 | | *Carpesium abrotanoides* | 35°14′ 36″ | 108°9′ 3″ | 371 | Asteraceae | Dicotyledonous | 0.096 | 7 | -144 | -150 | | | | | | | |
| Y-3 | | *Carpesium abrotanoides* | 35°14′ 36″ | 108°9′ 3″ | 371 | Asteraceae | Dicotyledonous | 0.08 | 5 | -145 | -149 | | | | | | | |
| Y-4 | center | *Carpesium abrotanoides* | 35°14′ 36″ | 108°9′ 3″ | 371 | Asteraceae | Dicotyledonous | 0.252 | -22 | -147 | -127 | | | | | | | |
| | | | | | | | | | | | | | | | | | | |
| K-1 | edge | *Populus L.* | 35°14′ 36″ | 108°9′ 3″ | 371 | Salicaceae | Dicotyledonous | 0.6 | -15 | -123 | -110 | -138 | -120 | -24 | -134 | -113 | -4 | -7 |
| K-2 | | *Populus L.* | 35°14′ 36″ | 108°9′ 3″ | 371 | Salicaceae | Dicotyledonous | 0.589 | -22 | -134 | -115 | | | | | | | |
| K-3 | center | *Populus L.* | 35°14′ 36″ | 108°9′ 3″ | 371 | Salicaceae | Dicotyledonous | 0.65 | -22 | -154 | -135 | | | | | | | |
| A-1 | tip | *Populus L.* | 35°14′ 36″ | 108°9′ 3″ | 371 | Salicaceae | Dicotyledonous | 0.432 | -19 | -124 | -107 | | | | | | | |
| A-2 | | *Populus L.* | 35°14′ 36″ | 108°9′ 3″ | 371 | Salicaceae | Dicotyledonous | 0.757 | -23 | -134 | -113 | | | | | | | |
| A-3 | base | *Populus L.* | 35°14′ 36″ | 108°9′ 3″ | 371 | Salicaceae | Dicotyledonous | 0.713 | -19 | -151 | -134 | | | | | | | |

[a] Caculated $\delta D_{C29\ n-alkane} = \sum_{i=1}^{n} \delta D_i * m_i(\%)$. $\delta D_i$ represents leaf wax $\delta D_{C29\ n-alkane}$ values at segmented i section along the leaf blade; $m_i(\%)$ represents percentage of weight at segmented i section.

[b] Caculated $\varepsilon_{wax-lw} = \sum_{i=1}^{n} \varepsilon_i * m_i(\%)$. $\varepsilon_i$ represents fractionation factor between leaf wax $\delta D_{n\text{-alkane}}$ values and corresponding leaf water δD values at segmented i section along the leaf blade; $m_i(\%)$ represents percentage of weight at segmented i section.

[c] $\Delta(\delta D_{C29\ n-alkane} - \delta D'_{C29\ n-alkane})$ = Caculated $\delta D_{C29\ n-alkane} - \delta D'_{C29\ n-alkane}$

[d] $\Delta(\varepsilon_{wax-lw} - \varepsilon'_{wax-lw})$ = Caculated $\varepsilon_{wax-lw} - \varepsilon'_{wax-lw}$

Table S5 The δD values of leaf water and corresponding leaf wax *n*-alkane in Xi'an and Lantian, China (‰).

| Sample date | Species | Location | Latitude | Longitude | Altitude/m | Plant life forms | Family | Plant taxonomy | $\delta D_{leaf\ water}$ | $\delta D_{n\text{-}alkane}$ |
|---|---|---|---|---|---|---|---|---|---|---|
| Nov.(2011) | *Buddleja alternifolia Maxim.* | Xi'an | 34°20′N | 108°56′E | 384 | shrub | Loganiaceae | dicotyledonous | -24 | -159 |
| Nov.(2011) | *Coriaria nepalensis Wall.* | Xi'an | 34°20′N | 108°56′E | 384 | shrub | Coriariaceae | dicotyledonous | -18 | |
| Nov.(2011) | *Cirsium setosum* | Xi'an | 34°20′N | 108°56′E | 384 | herb | Asteraceae | dicotyledonous | -7 | -173 |
| Nov.(2011) | *Cherry blossom* | Xi'an | 34°20′N | 108°56′E | 384 | tree | Rosaceae | dicotyledonous | -26 | -173 |
| Nov.(2011) | *Swida macrophylla* | Xi'an | 34°20′N | 108°56′E | 384 | tree | Cornaceae | dicotyledonous | -6 | -169 |
| Nov.(2011) | *Ligustrum lucidum Ait* | Xi'an | 34°20′N | 108°56′E | 384 | tree | Oleaceae | dicotyledonous | -15 | -164 |
| Nov.(2011) | *Potentilla kleiniana* | Xi'an | 34°20′N | 108°56′E | 384 | grass | Rosaceae | dicotyledonous | -17 | -156 |
| Sept.(2012) | *Populus tomentosa(0cm)* | Lantian | 34°14′N | 109°7′E | 619 | tree | Salicaceae | dicotyledonous | -38 | -154 |
| Sept.(2012) | *Populus tomentosa(50cm)* | Lantian | 34°14′N | 109°7′E | 619 | tree | Salicaceae | dicotyledonous | -47 | -157 |
| Sept.(2012) | *Populus tomentosa(100cm)* | Lantian | 34°14′N | 109°7′E | 619 | tree | Salicaceae | dicotyledonous | -38 | -161 |
| Sept.(2012) | *Populus tomentosa(200cm)* | Lantian | 34°14′N | 109°7′E | 619 | tree | Salicaceae | dicotyledonous | -47 | -150 |
| Sept.(2012) | *unidentified(0cm)* | Lantian | 34°14′N | 109°7′E | 619 | tree | unidentifed | dicotyledonous | -34 | -156 |
| Sept.(2012) | *unidentified(100cm)* | Lantian | 34°14′N | 109°7′E | 619 | tree | unidentifed | dicotyledonous | -36 | -172 |
| Sept.(2012) | *unidentified(200cm)* | Lantian | 34°14′N | 109°7′E | 619 | tree | unidentifed | dicotyledonous | -40 | -176 |
| Sept.(2012) | *Lespedeza davurica(Laxm.) Schindl.* | Lantian | 34°14′N | 109°7′E | 619 | shrub | Leguminosae | dicotyledonous | -30 | -172 |
| Sept.(2012) | *Cirsium shansiense Petrak* | Lantian | 34°14′N | 109°7′E | 619 | herb | Asteraceae | dicotyledonous | -15 | -158 |
| Nov.(2011) | *Lolium perenne L.* | Xi'an | 34°20′N | 108°56′E | 384 | grass | Poaceae | monocotyledonous | -22 | -182 |
| Nov.(2011) | *Agrostis matsumurae* | Lantian | 34°14′N | 109°7′E | 619 | grass | Poaceae | monocotyledonous | -15 | -179 |
| Sept.(2012) | *Bothriochloa ischaemum* | Xi'an | 34°20′N | 108°56′E | 384 | grass | Poaceae | monocotyledonous | -40 | -207 |
| Sept.(2012) | *Stipa bungeana Trin.* | Lantian | 34°14′N | 109°7′E | 619 | grass | Poaceae | monocotyledonous | -23 | -222 |
| Sept.(2012) | *Agropyron cristatum(L.) Gaertn.* | Lantian | 34°14′N | 109°7′E | 619 | grass | Poaceae | monocotyledonous | -35 | -185 |

**Fig S1.** The size of the leaf blade of *Rhleum palmatum L.* (dicot) and *Hierochloe glabra* (monocot). The two species were adjacent to grow on the Chinese Loess Plateau where they received full sun and natural rainfall, without human disturbance.

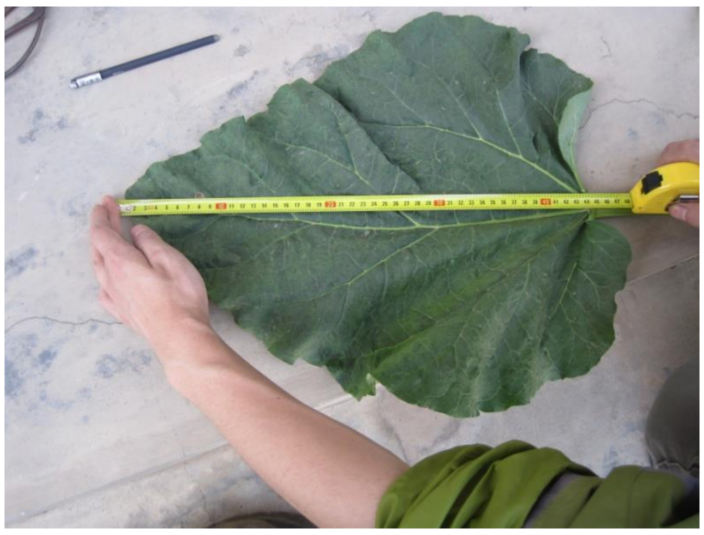

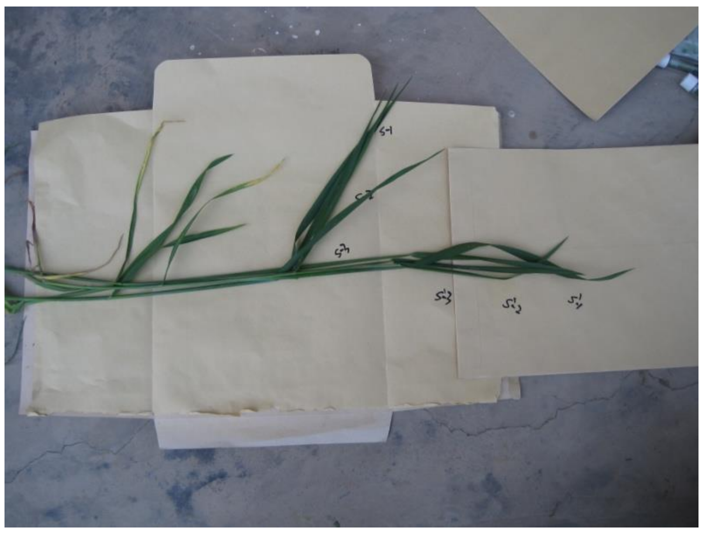

**Fig. S2** Leaf wax δ$D_{n\text{-alkane}}$ values between woods (shrubs and trees) and herbaceous plants from Lantian and Xi’an (Liu et al., 2015) and from Heshui County (site 3) (soild red circles represent herbaceous plants, black circles signify woods). Apart from Lantian and Xi’an, no significant difference in leaf wax δ$D_{n\text{-alkane}}$ values between woods and herbaceous plants in Heshui County was observed.

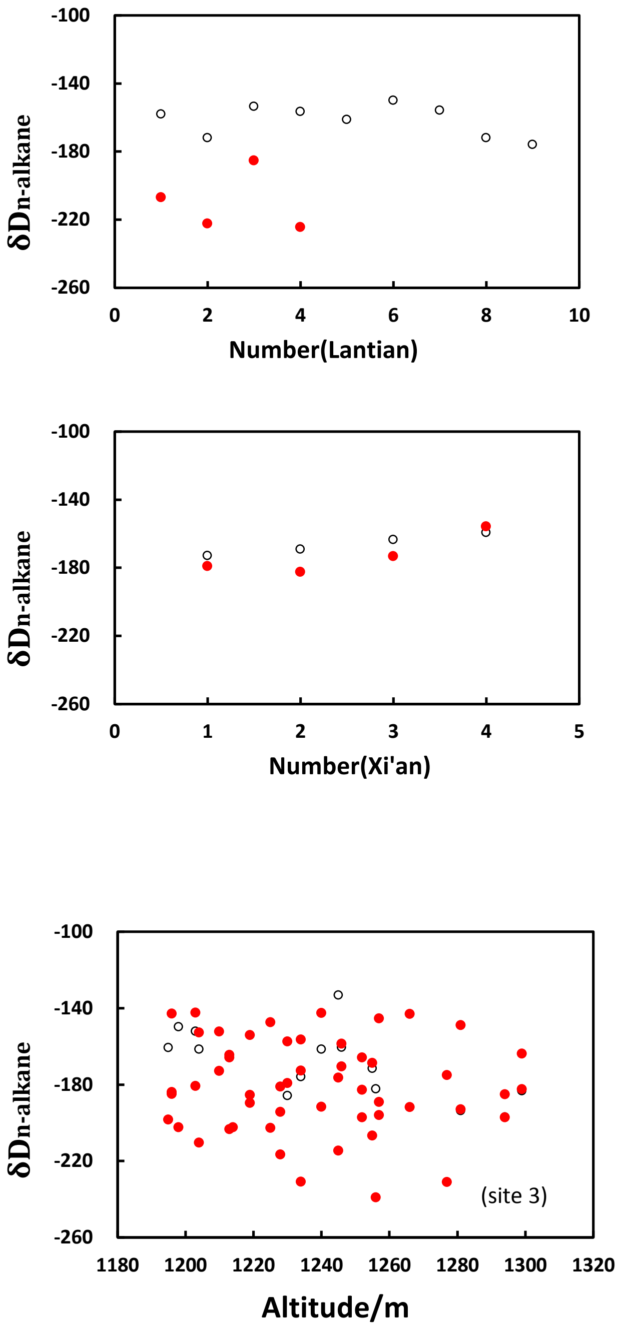

Fig. S3 Leaf wax $\delta D_{n\text{-alkane}}$ and $\varepsilon_{wax\text{-}p}$ values in gymnosperms varied along the latitude.

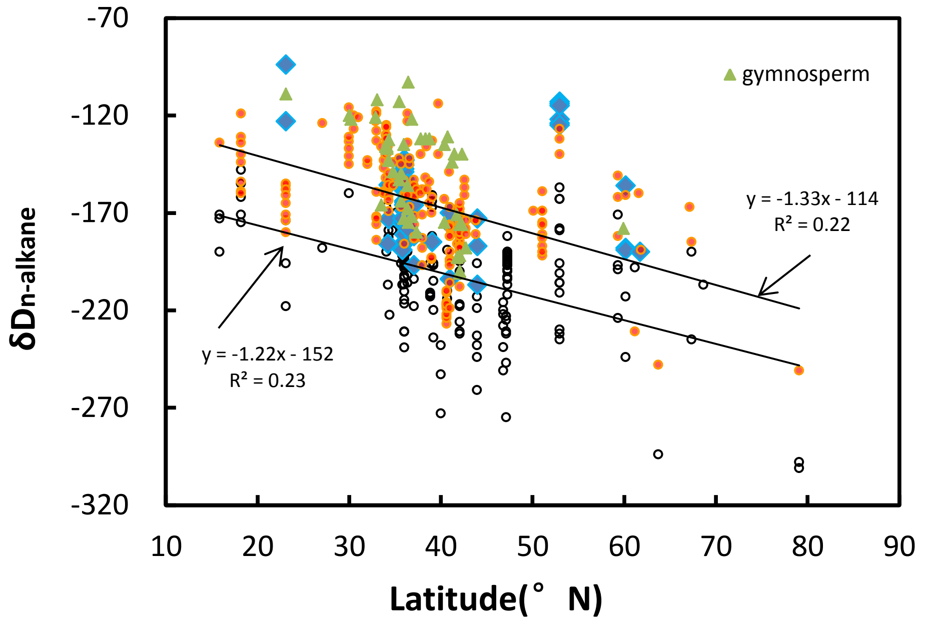


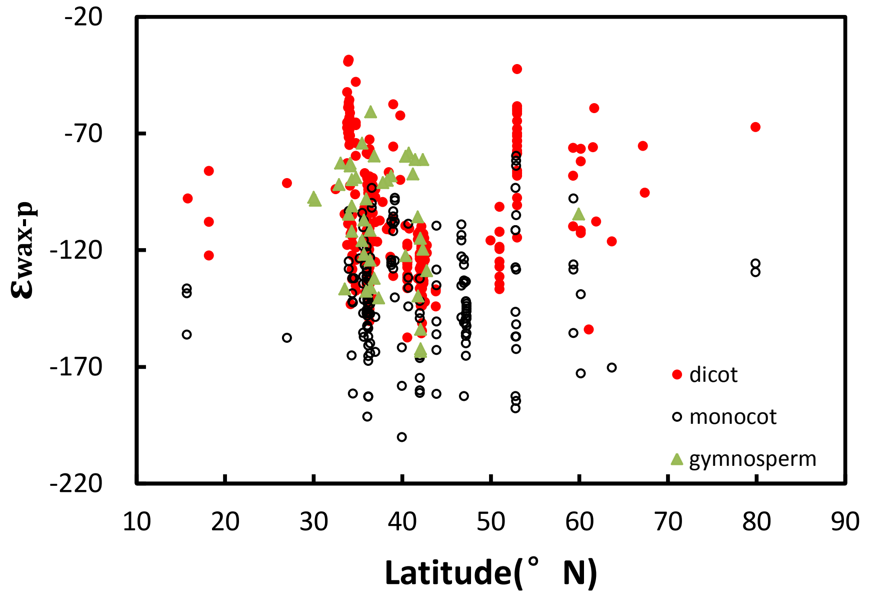

Fig. S4 Leaf wax $\delta D_{n\text{-alkane}}$ values between dicots and monocots in herbaceous plants collected from the North Hemisphere. One-way ANOVA test showed that the average $\delta D_{n\text{-alkane}}$ values of leaf wax differed significantly between monocotyledonous and dicotyledonous herbaceous plants ($P < 0.001$). (soild red circles represent herbs and forbs, black circles signify grass)

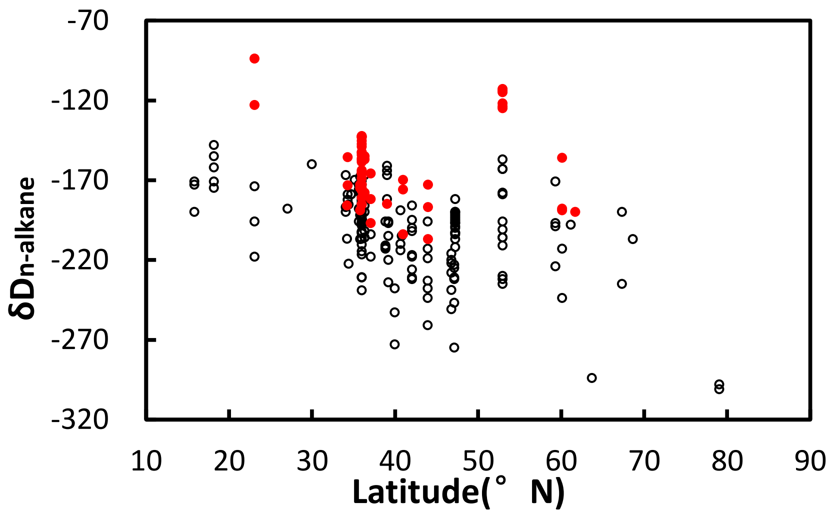

**Fig S5.** Showing leaf segmented sections where dicotyledonous balde in *Rheum palmatum L.* was cut into sections from base to tip along the main veins and edges of blade, whereas monocotyledonous baldes in *Hierochloe galbra* from base to tip. The $\varepsilon_{wax\text{-}lw}$ values between leaf wax and leaf water in every segmented sections varied along the leaf blade and corresponding leaf water δD values in brackets (‰). The results indicated the $\varepsilon_{wax\text{-}lw}$ values inside leaf blade were constant, but differed significantly between dicotyledonous and monocotyledonous species.

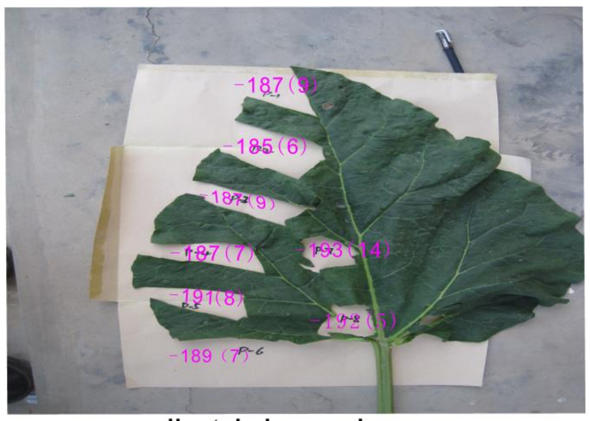


dicotyledonous leaves

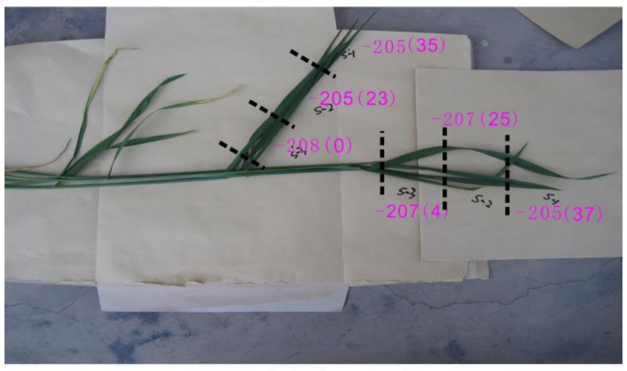


monocotyledonous leaves

**Fig S6** Leaf wax δD$_{n\text{-alkane}}$ values between monocotyledonous and dicotyledonous species complied from different sampling sites in this study. Note: site 1 (Lantian: 34°14′N, 109°7′E); site 2 (Xi’an: 34°20′N, 108°95′ E); site 3 (Heshui County: 36°01'N, 108°06'N); site 4 (Luochuan: 35°49′ N, 109°30′ E); site 5 (Yuxian: 37°05′ N, 113°02′ E); site 6 (Ruicheng: 34°44′ N, 110°25′ E); site 7(Ertuokeqi: 39°05′ N, 107°58′ E); site 8(Yanan: 36°35′ N, 109°29′ E); site 9 (Huining, Baishui, Neimeng, Pengyang, Jitantai, Yanchi, Yijun, Yingchuan, Lanzhou etc.); site 10 (Axel Heiberg Island: 79°54′ N, 89°01′ W); site 11 (Denali: 63°43′ N, 148°54′ W); site 12 (Helsinki: 60°10′ N, 24°56′ E); site 13 (Stockholm: 59°20′ N, 18°02′ E); site 14(Gunma-Japan: 36°23′ N, 139°37′ E); site 15 (Tainland: 15°52′ N, 100°59′ E); site 16 (Tokyo-Japan: 35°41′ N, 139°30′ E); site 17(Blood pond: 42°08′N, 71°96′W); site 18(Stiffkey: 52°58′N, 0°55′E).

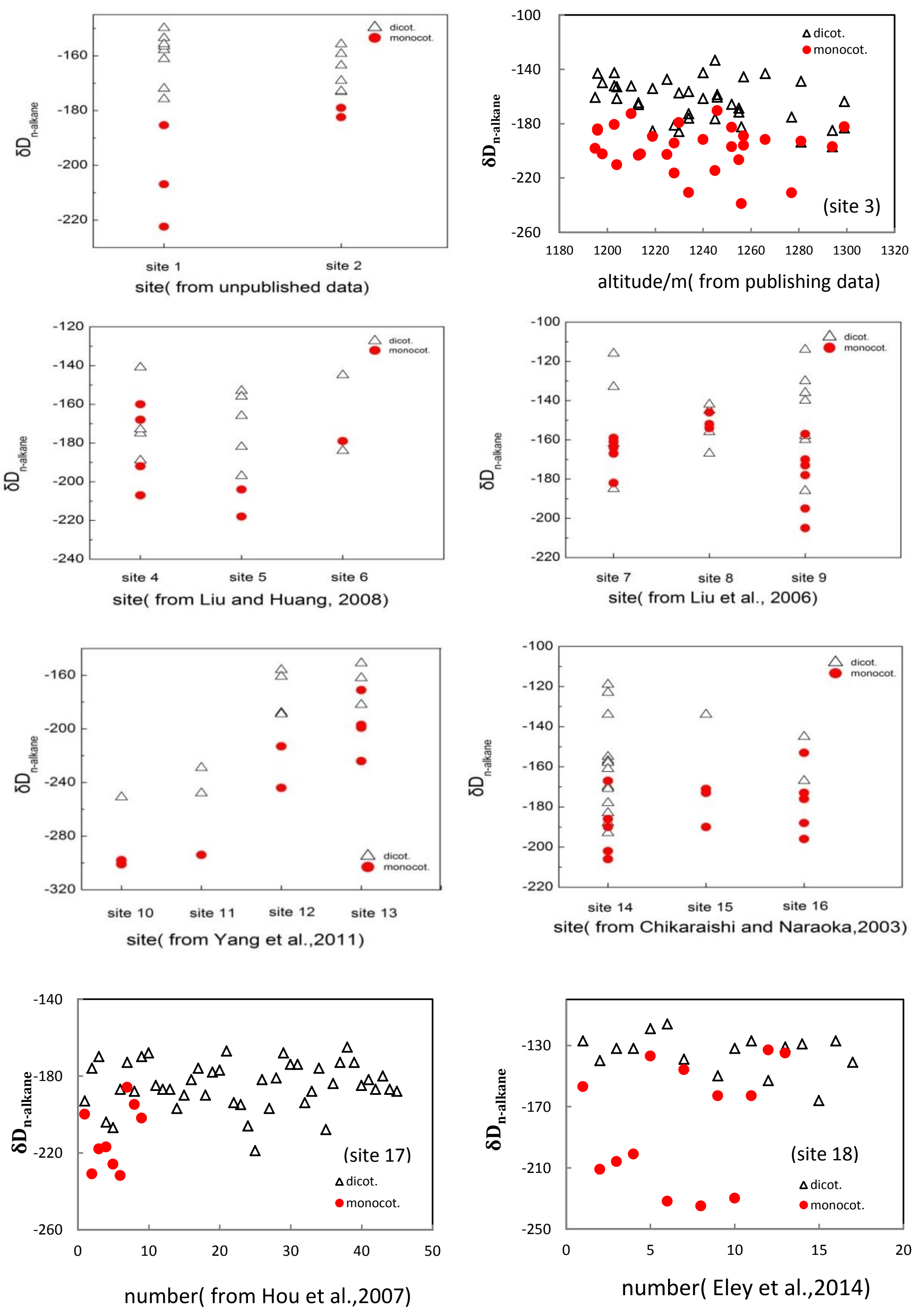

Supplement: Supplementary Information [file srep19711-s1.pdf]
